# A Systematic Review of Nutritional Lab Correlates with Chemotherapy Induced Peripheral Neuropathy

**DOI:** 10.3390/jcm11020355

**Published:** 2022-01-12

**Authors:** Cindy Tofthagen, Mary Tanay, Adam Perlman, Jason Starr, Pooja Advani, Katharine Sheffield, Tara Brigham

**Affiliations:** 1 Division of Nursing Science, Mayo Clinic, Jacksonville, FL 32224, USA; sheffield.katharine@mayo.edu; 2 Faculty of Nursing, Midwifery and Palliative Care, King’s College London, London SE1 8WA, UK; mary.tanay@kcl.ac.uk; 3 Division of General Internal Medicine, Mayo Clinic, Jacksonville, FL 32224, USA; perlman.adam@mayo.edu; 4 Division of Hematology/Oncology, Mayo Clinic, Jacksonville, FL 32224, USA; starr.jason@mayo.edu (J.S.); Advani.Pooja@mayo.edu (P.A.); 5 Mayo Clinic Libraries, Mayo Clinic, Jacksonville, FL 32224, USA; brigham.tara@mayo.edu

**Keywords:** nutritional deficiency, laboratory, neuropathy, neurotoxicity, chemotherapy, chemotherapy induced peripheral neuropathy, CIPN

## Abstract

Chemotherapy induced peripheral neuropathy (CIPN) is a dose-limiting side effect of chemotherapy for which no prevention or cure exists. Cancer and cancer treatments can adversely affect nutritional status. Nutrition may play a role in development of CIPN, yet the relationship between nutrition and CIPN is not well understood. Common laboratory values measuring various aspects of nutrition (hemoglobin/hematocrit, vitamin B12, calcium, and magnesium) may be associated with CIPN. The aim of this systematic review is to evaluate the empirical evidence surrounding the relationship between laboratory measures of nutrition and CIPN among persons with cancer who received neurotoxic chemotherapy drugs. We conducted an extensive review of the literature to identify articles that evaluated relationships between laboratory measures of nutrition and CIPN. A total of eleven articles satisfied the inclusion/exclusion criteria. Participants in the studies had breast or colorectal cancer, lymphoma or multiple myeloma and were receiving a variety of neurotoxic drugs. Hemoglobin/hematocrit, vitamin D, albumin, and magnesium were associated with CIPN. The quality of the studies ranges from fair to good. Evidence suggests that low levels of the above-mentioned tests could be associated with CIPN but additional research is needed.

## 1. Introduction

Despite advances in the treatment of cancer, chemotherapy induced peripheral neuropathy (CIPN) remains a common, often dose-limiting adverse effect of multiple chemotherapeutic agents. The incidence of development of CIPN may be as high as 85% among persons who receive neurotoxic chemotherapy drugs [1,2,3]. CIPN is difficult to prevent or treat and adversely affects physical function and quality of life [4,5]. CIPN symptoms continue to develop and progress for several months post-therapy and can persist for many years following treatment, in many cases becoming permanent [2,6]. Dose modifications and treatment interruptions in response to the development of CIPN can adversely affect treatment efficacy and cancer outcomes [7]. 

Good nutrition is essential for the functioning of the peripheral nervous system. Deficiencies in vitamin B12, vitamin B1, folate, vitamin E, and copper independently contribute to the development of peripheral neuropathy, as do excess levels of vitamin B6 [8]. Cancer and cancer treatments can adversely affect nutritional status, including both intake and absorption of nutrients. For example, surgical resection of the small intestine can reduce absorption of iron and vitamin B12, resulting in deficiencies of these critical nutrients [9]. Therefore, laboratory measures of nutrition provide better information about nutritional status than evaluation of dietary intake.

Nutrition may also play a role in development of CIPN, yet the relationship between nutrition and CIPN is not well understood [8]. Certain vitamins and minerals, such as B vitamins, vitamin D, and magnesium, are important in neurologic function and may play a role in the development of CIPN. Several routine laboratory tests measure aspects of nutrition that may provide insight into the potential relationship between nutrition and CIPN. While several known potential risk factors for the development of CIPN exist, including medical comorbidities such as diabetes and lifestyle factors such as obesity, there is a need to better understand the individual risk factors that could increase a patient’s susceptibility to the development on CIPN [7]. The aim of this systematic review is to evaluate the empirical evidence surrounding the relationship between laboratory measures of nutrition and CIPN among persons with cancer who received neurotoxic chemotherapy drugs. This information is needed to guide future research and inform clinical care.

## 2. Methods

### Search Strategy

**Figure 1:**
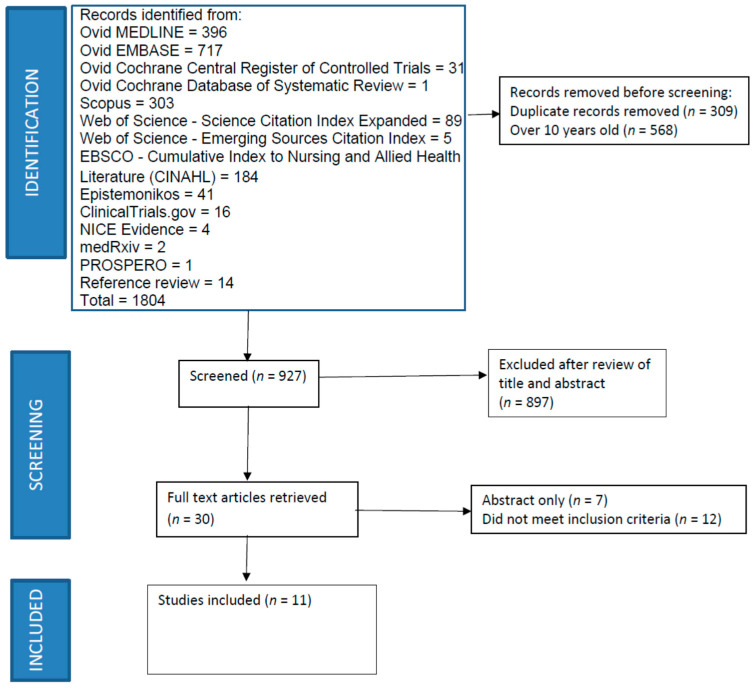
PRISMA Diagram for Identification and Review of Pertinent Articles.

We conducted a systematic review to identify articles that examine the relationship between CIPN and a nutritional laboratory measure (hemoglobin/hematocrit, serum albumin, serum vitamin B12, etc.). The full search strategies are available in the Supplementary Materials. We conducted a systematic review to identify articles that examine the relationship between CIPN and a nutritional laboratory measure. Studies were identified by a medical librarian developing and running searches in the MEDLINE (1946–present), Embase (1974–present), Cochrane Central Register of Controlled Trials (1991–present), and Cochrane Database of Systematic Review (2005–present) [all via the Ovid interface]; Scopus (1823–present); Science Citation Index Expanded (1975–present) and Emerging Sources Citation Index (2015–present) [both via the Web of Science interface] and CINAHL with Full Text (1963–present) (via the EBSCOhost interface) databases. Grey literature resources were also searched (Figure 1). There were no limits to language or publication date in the initial search strategy. Filters to remove animal, pediatric, case studies or reports were employed. Search terms included MeSH, Embase/Emtree terms, as well as keywords such as chemotherapy induced peripheral neuropathy, laboratory tests or values, and nutrition (see Supplementary Materials). All databases were searched on 4 June 2020 and all grey literature resources were searched on 5 June 2020. A draft search strategy was peer reviewed by a second information professional. Upon review of the initial search results, included citations were restricted to studies published in English from 1 June 2010 to the search date (4 or 5 June 2020). An updated search was run on 8 December 2021. Expert opinion, reports of on-going studies, studies including non-cancer populations, studies with no abstract available, only the abstract available, non-peer reviewed articles, review articles, meta-analyses, were also excluded. The authors also reviewed the reference lists of selected studies and published review articles for other potential studies.

## 3. Results

The search resulted in identification of 927 potential publications (Figure 1). The abstracts were screened for initial relevance to the topic by two authors (CT and MT). Following review of abstracts, 30 were identified for full-text review. Following independent review of those full-text articles, a total of 11 articles were identified that satisfied the followitableng inclusion/exclusion criteria: (1) described a relationship between nutritional laboratory measures and CIPN; (2) published in English after June 2010; (3) not a case studies, expert opinion, on-going study, interventional design, review article, meta-analysis, or animal study. Articles with no abstract available, only the abstract available, or those in non-cancer or pediatric populations were excluded. Each included article was abstracted and evaluated for quality and bias by two authors. 

### 3.1. Methodological Quality

Guided by the NIH Quality Assessment for observational cohort and cross-sectional studies, all authors independently carried out methodological quality assessment of studies (https://www.nhlbi.nih.gov/health-topics/study-quality-assessment-tools, accessed on 1 June 2020). Four studies were rated good, four were fair–good and another three were rated fair. Most studies did not provide any explanation about whether outcome assessors were aware of the exposure status of the participants. The observational nature of the studies increases the risk for confounding bias, specifically variables such as age, pre-existing conditions, or overall health status. 

### 3.2. Study Characteristics

Participants. Overall, a total of 1176 cancer patients participated across studies included in this review. Individual study sample ranged between 37 and 186 participants. Recruited by convenience sampling, participants in the studies were diagnosed with breast [10,11,12], colorectal [13,14,15,16], lymphoma [17], or multiple myeloma (Table 1) [18]. One study included participants with various cancer types [19]. The majority of the studies were conducted outside of the United States (*n* = 3) including Italy (*n* = 2), Japan (*n* = 1), Czech Republic (*n* = 1), Spain (*n* = 1), Netherlands (*n* = 1), Turkey (*n* = 1), and Iran (*n* = 1). Race and ethnicity were reported in two studies [11,18], with non-minorities making up approximately 80% of the sample in both studies (Table 2). An additional study reported race as either white or non-white only [10].

Study Design. Five studies included in this review used a retrospective observational design [10,13,15,17,19] while six used an observational design prospectively [11,12,14,16,18,20]. 

Comorbidities. There was inconsistent reporting of comorbid medical conditions across studies (Table 3). The most common assessed comorbidities were diabetes/hyperglycemia, which was assessed in nine of the eleven included studies and affected 6.6–21.6% of participants. Alcohol use was assessed in four of the eleven and noted in 3.8–62.3% of participants.

CIPN Measurement. Most studies used clinician-graded scales such as the Common Terminology Criteria for Adverse Events (CTCAE) (*n* = 7) and Total Neuropathy Score (*n* = 2). Three studies used validated self-report measures for CIPN including one study that used the European Organization for Research and Treatment of Cancer Quality of Life Questionnaire-CIPN twenty-item scale (EORTC-CIPN 20) [16], and one used an 8 item version [10]. Another study used the Functional Assessment of Cancer Therapy Scale/Gynecologic Oncology Group-Neurotoxicity (FACT/GOGNTx-13) [18] Another study used the Michigan Neuropathy screening instrument that combines clinician grading and patient-reported symptoms [12].

### 3.3. Results by Chemotherapy Type

#### 3.3.1. Platinum-Based Chemotherapies

Five studies examined laboratory measures associated with CIPN caused by oxaliplatin [13,14,15,16,19]. There were no studies identified that included individuals getting cisplatin, another highly neurotoxic platinum analog. 

A prospective, observational study of 130 patients from Iran with stage III colorectal cancer treated with adjuvant capecitabine and oxaliplatin (CAPEOX) or infusional 5-FU and oxaliplatin (FOLFOX) sought to evaluate risk factors for chronic neuropathy [14]. Neuropathy was graded using the CTCAE v.3 scale. Patients with a history of receiving other neurotoxic agents, pre-existing neuropathy, polymyositis, kidney, or liver dysfunction were excluded from the study. Eighty-seven (66.9%) patients received FOLFOX (oxaliplatin dose 85 mg/m^2^) and 43 (33.1%) received CAPEOX (oxaliplatin dose 130 mg/m^2^). Neuropathy was evaluated at baseline, 3 months (middle of treatment), 6 months (end of treatment), and then 3 and 6 months thereafter. Of the 130 patients, 105 (80.7%) developed neuropathy (grade 1–3) at some point during that interval, while 25 (19.2%) did not. Anemia and hypomagnesaemia were more common among the 80.7% who developed neuropathy (*p* < 0.05). Anemia was also associated with severity of neuropathy (*p* < 0.0001). Information about how anemia and hypomagnesaemia were defined in this study was not provided.

A retrospective study of 102 patients from Italy with stage II/III colorectal cancer treated with adjuvant capecitabine and oxaliplatin sought to determine the prognostic and predictive factors for the development of peripheral neuropathy from oxaliplatin [13]. Neuropathy was graded using the CTCAE v4.03 scale. Of the patients analyzed the median number of cycles received was 8 (range 3–8). The dose of oxaliplatin was 130 mg/m^2^. Approximately 18% (*n* = 18) of patients developed clinically significant (defined as grade 2 or 3) neurotoxicity (8 acute, 10 chronic). Diabetes was significantly associated with development of chronic neuropathy; however, among diabetics, glycosylated hemoglobin levels were not associated with development of neuropathy (*p* = 0.86). Cholesterol and triglycerides levels were not significantly associated with neuropathy in this study. The small (*n* = 10) sample of patients who developed chronic neuropathy was a limitation that may have contributed to these findings. It is unclear as to why fewer patients developed neuropathy in this study, since similar neuropathy measures and data collection points were used in this study as in others where CIPN occurred in 52–81% of participants [14,15].

Another retrospective, Italian study involving 169 stage III colorectal cancer patients sought to identify predictors of CIPN among patients treated with oxaliplatin [15]. Neuropathy was graded using the CTCAE version 3. Forty-eight percent (*n* = 81) of patients had no functional impairment from neuropathy (grade 0–1) while 52% (*n* = 88) developed neuropathy that interfered with function (grade 2–3). Ninety percent received 7–12 cycles of 85 mg/m^2^ of oxaliplatin. Hypoalbuminemia, defined as serum albumin level <3.5 gr/dL, was present in 80% of patients (*n* = 136). Anemia, defined as hemoglobin level <12 g/dL for women and <13 g/dL for men, was present in 55% (*n* = 93) of the sample. Hypomagnesemia, defined as serum magnesium <1.8 mEq/L, was present in 26% (*n* = 44) and hypocalcemia, defined as a serum ionized calcium <1.1 mmol/L, was present in 20% (*n* = 34). Anemia (*p* = 0.001), hypoalbuminemia (*p* = 0.01), and hypomagnesemia (*p* = 0.001) were significantly associated with grade (2–3) and duration of neuropathy. No association between calcium levels and neuropathy was present. 

A Turkish research team sought to retrospectively identify common clinical and laboratory measures associated with the development of CIPN among 186 colorectal patients treated with an oxaliplatin-based chemotherapy regimen containing either 85 mg/m^2^ or 130 mg.m^2^ [19]. CIPN was graded using the CTCAE and categorized as either grade 0–1 (*n* = 108, 58%) or grade 2–3 (*n* = 78, 42%). Prior to treatment several nutritional laboratory measures were collected (Table 4). Vitamin D and hemoglobin levels were found to be associated with grade 2–3 neuropathy using multiple logistic regression. Glucose levels were higher (*p* = 0.007) and albumin (*p* = 0.001), magnesium (*p* = 0.04), hemoglobin (*p* < 0.001) and vitamin D (*p* < 0.001) levels were lower in the group with grade 2–3 CIPN compared to the group with grade 0–1 CIPN. No differences in CIPN based on chemotherapy regimen and stage of malignancy were identified; however, grade 2–3 neuropathy was more prevalent among those with mucinous carcinoma (*p* < 0.001), with 88% of those patients having grade 2–3 CIPN.

Oxidative stress been proposed as a contributor to development of CIPN [21]. Ergothioneine is a dietary antioxidant that could potentially mitigate development of CIPN. A prospective study conducted at 11 hospitals in the Netherlands sought to explore whether ergothioneine levels in whole blood were associated with chronic oxaliplatin induced CIPN [16]. One hundred fifty-nine participants who received oxaliplatin or capecitabine had ergothioneine levels drawn prior to initiation of chemotherapy and peripheral neuropathy was assessed 6 months after completion of chemotherapy to determine relationships between ergothioneine levels prior to chemotherapy and the development of chronic CIPN using a 16-item version of the European Organization for Research and Treatment of Cancer-Quality of Life, Chemotherapy-Induced Peripheral Neuropathy 20 item (EORTC-QLQ-CIPN20). A cut score of more than 3.6 was used to categorize participants into two groups, those with CIPN and those without. Relationships between CIPN and ergothioneine levels were examined both with ergothioneine as a continuous variable as well as categorized into tertiles. Multiple analyses were performed yet no statistically significant relationships between CIPN and ergothioneine were detected. The 12 month time period between testing of ergothioneine levels and assessment of CIPN may have impacted their results. The authors suggested that future studies include multiple timepoints and include examination of dietary intake of mushrooms, as this is the primary dietary source of ergothioneine.

#### 3.3.2. Taxanes

Two studies examined laboratory measures of nutrition associated with paclitaxel therapy in breast cancer patients [10,11]. Jennaro et al. (2020) investigated the relationship between baseline deficiency of vitamin D, vitamin B12, folate, or homocysteine and CIPN in patients with breast cancer receiving weekly paclitaxel. Results regarding vitamin D deficiency are discussed in another section of this paper. No statistically significant relationships between baseline vitamin B12, folate, or homocysteine and CIPN were noted, likely due to the small amount of participants who had any of these deficiencies.

Robertson et al. (2018) also explored possible factors that predispose breast cancer patients receiving paclitaxel to neuropathy development. Among 61 participants, laboratory values were collected at baseline and four months after initiation of paclitaxel treatment. These included glycosylated hemoglobin (HbA1c), vitamin B12, vitamin B6, vitamin E, folate, lipid panel (high-density lipoprotein (HDL), low-density lipoprotein (LDL), and triglycerides), albumin, prealbumin, lactic acid dehydrogenase (LDH), blood urea nitrogen (BUN), creatinine, aspartate aminotransferase (AST), alanine aminotransferase (ALT), thyroid stimulating hormone, serum immunofixation, erythrocyte sedimentation rate (ESR) and antinuclear antibody. Multivariate regression analysis identified predictors that were significantly associated with worsening neuropathy based on Total Neuropathy Score (TNS) included low serum albumin (*p* = 0.002), paclitaxel dose (*p* = 0.001), and increased body surface area (*p* = 0.006). Higher triglycerides (*p* = 0.20) had non-significant association with increased neuropathy scores. 

A prospective study of 70 patients undergoing weekly paclitaxel (80 mg/m^2^) for breast cancer examined relationships between vitamin B1 and B6, omega 3 fatty acids, vitamin D, and CIPN [12]. This study measured the above laboratory values at three timepoints; prior to initiation of chemotherapy, 4 weeks after initiation, and at completion of the 12 planned cycles of chemotherapy. CIPN was assessed using the Michigan Neuropathy Screening Instrument, which combines self-reported symptoms and physical exam. Patients were divided into two groups for analysis based upon whether they had any neuropathy symptoms or not. The patients who developed CIPN symptoms had significantly lower vitamin D (*p* = 0.008) and higher unsaturated fatty acids (C12:0 *p* = 0.03, C14:0 *p* = 0.04, C16:1 *p* = 0.03), and total saturated fatty acids (*p* = 0.01) prior to chemotherapy. Vitamin D levels were significantly lower among the patients with CIPN at all three timepoints (*p* < 0.05). No associations were found between CIPN and vitamin B1 or B6.

#### 3.3.3. Taxanes or Platinums

A prospective, longitudinal study among 113 patients receiving either a taxane or a platinum sought to evaluate the role of micronutrients in CIPN recovery [20]. The CTCAE was used to categorize neuropathy as either not clinically relevant (grade 0–1) or clinically relevant (grade 2–3). CIPN recovery was defined as a decrease in the grade of CIPN by 1 grade or more over time. Nutritional measures including vitamin E, magnesium, prealbumin, and vitamin B12 were collected before chemotherapy, 1–3 months and 12 months following chemotherapy. CIPN was assessed at the same timepoints with the CTCAE, nerve conduction studies, as well as two versions of the Total Neuropathy Score (TNSc and TNSr). No significant differences between any of the nutritional measures and CIPN were identified during the timeframe from prior to chemo until 1–3 months afterward. At the end of the study, patients who did not recover had a decrease in vitamin E (*p* = 0.02) and prealbumin (*p* = 0.06) compared to those who recovered, suggesting that nutrition affects CIPN recovery, and that vitamin E may promote CIPN recovery. 

#### 3.3.4. Other Agents 

The search yielded two studies investigating CIPN outside of taxanes and platinum-based agents, one in multiple myeloma patients receiving bortezomib or thalidomide and another in lymphoma patients receiving vincristine. 

A cross-sectional, descriptive study sought to evaluate the relationship between vitamin D levels and development of CIPN among 111 multiple myeloma patients treated with bortezomib or thalidomide [18]. Serum collection from all patients showed a median serum vitamin D level of 32 ng/mL with a range from 11.4–85.5. Sixty-eight percent of participants had low vitamin D levels (<20.0 ng/mL) and 59% had at least mild neuropathy as measured by the FACT/GOGNTx-13. Associations between CIPN symptoms and vitamin D levels are reported under the section below with the heading of vitamin D levels. 

Finally, a retrospective study conducted in Japan sought to identify predictors of CIPN among 40 lymphoma patients receiving rituximab plus cyclophosphamide, doxorubicin, vincristine, and prednisolone therapy. Patients were grouped for comparison as having mild (grade 0–1) or severe (grade 2–3) neuropathy using the CTCAE. The highest grade of neuropathy reported during chemotherapy was used and glucose, hemoglobin, albumin, and potassium data from before chemotherapy initiation were used. The number of participants with severe CIPN was small (Table 1). Anemia was present in 85.7% of participants with grade 2–3 neuropathy, compared to 30.3% of those with grade 0–1 neuropathy (*p* < 0.01). Further, step wise regression demonstrated that pre-treatment anemia was an independent predictor of severe CIPN (OR = 19.45, 95% confidence interval = 1.52–171.12). No significant relationships between glucose, albumin, or potassium levels and CIPN were identified; however, all of the participants with low albumin or high glucose levels developed mild CIPN and 75% of the patients with abnormal potassium levels were also in the mild CIPN group. 

### 3.4. Results by Laboratory Parameter

#### 3.4.1. Hemoglobin

Four studies described a relationship between anemia and CIPN [14,15,17,19]. One study involved lymphoma patients who received a regimen of rituximab, cyclophosphamide, doxorubicin, vincristine, and prednisone (R-CHOP) [17] and three were among persons with colorectal cancer receiving oxaliplatin [14,15,19]. In colorectal cancer patients receiving oxaliplatin, Shahriari-Ahmadi and colleagues (2015) compared results of those who had neuropathy (*n* = 105) to those who did not (*n* = 25) and found anemia to be present in 81% (*n* = 85) of those with neuropathy, compared to 52% (*n* = 13) of those who did not (*p* = 0.004). Similarly, Vincenzi et al. found anemia in 56% of participants with grade 2–3 neuropathy compared to 44% of those with grade 0–1 neuropathy (*p* = 0.001). The Yildirim study reported anemia among 42% of persons with grade 0–1 CIPN compared to 63% of persons with grade 2–3 CIPN (*p* = 0.003). Anemia was defined in all studies as a hemoglobin level <12 g/dL for women and <13 g/dL for men [14,15,17,19]. Among lymphoma patients receiving the R-CHOP regimen with the neurotoxic agent being vincristine, anemia was present in 85.7% of participants with grade 2–3 neuropathy, compared to 30.3% of those with grade 0–1 neuropathy (*p* < 0.01). Further, step wise regression demonstrated that pre-treatment anemia was an independent predictor of severe CIPN (OR = 19.45, 95% confidence interval = 1.52–171.12). Findings from these studies suggest that pre-treatment anemia is a risk factor for CIPN caused by oxaliplatin and/or vincristine. No studies were included that evaluated associations between hemoglobin and CIPN among persons receiving taxanes.

#### 3.4.2. Vitamin D Levels 

Four studies described a relationship between vitamin D levels (25 hydroxy vitamin D) and CIPN [10,12,18,19], two among persons receiving paclitaxel for breast cancer, one among persons receiving oxaliplatin for colorectal, gastric, or pancreatic cancer and one among persons with multiple myeloma receiving bortezomib or thalidomide. One retrospective analysis of data collected during a prospective study included 37 Caucasian women with breast cancer who received weekly paclitaxel [10] and demonstrated that vitamin D-deficient patients experienced a greater mean CIPN increase when compared with those who have normal levels of vitamin D at baseline (vitamin D deficient = 36.39 ± 22.80 vs. normal vitamin D levels = 16.29 ± 16.30, *p* = 0.003). There was an increase in the risk of treatment interruption among those who were vitamin D deficient, although the reasons for interruption were not described, nor was it statistically significant (OR 2.98, 95% CI (0.72, 12.34), *p* = 0.16). When adjusted for clinical and treatment variables, baseline vitamin D level and CIPN were inversely associated, although this association was not particularly strong (β = −0.04, *p* = 0.02). 

A second prospective study among 70 patients receiving paclitaxel (90% female) for breast cancer demonstrated significantly lower median levels of vitamin B12 among persons who developed CIPN (25.6 vs. 38.2 nmol/L, *p* = 0.008) [12]. Further, a significant drop in vitamin B12 levels was observed during chemotherapy. 

Similar results were demonstrated in a retrospective of among persons receiving oxaliplatin for a gastrointestinal malignancy [19]. Mean baseline vitamin D levels were lower among persons who developed CIPN compared to those who did not (17.2 ± 11.4 vs. 10.2 ± 7.2, *p* < 0.001). Multivariate logistical regression demonstrated that Vitamin D was an independent predictor of grade 2–3 CIPN (*p* < 0.001). 

A prospective study by Wang and colleagues (2016) explored the relationship of vitamin D and CIPN due to bortezomib or thalidomide in multiple myeloma. Out of 109 participants, 16% were vitamin D deficient (<20.0 ng/mL) and 26% had vitamin D insufficiency (20–29.9 ng/mL). Analysis showed no relationship between vitamin D deficiency (*p* = 0.80) or insufficiency (*p* = 0.18–0.38) and occurrence of either motor or sensory neuropathy. However, in terms of CIPN severity (>grade 2), patients who were vitamin D deficient had more severe motor (*p* = 0.04) and sensory symptoms (*p* = 0.01). There was no relationship between vitamin D deficiency and pain levels.

#### 3.4.3. Magnesium Levels 

Four studies describing a relationship between magnesium levels and CIPN were identified [14,15,19,20]. All four of the studies included colorectal cancer patients receiving oxaliplatin. One of the studies also included persons with other malignancies who were receiving cisplatin, paclitaxel, or docetaxel. 

A prospective evaluation of 130 participants in Iran who were receiving oxaliplatin for colorectal cancer demonstrated an overall prevalence of hypomagnesemia of 13.8% [14]. There were significant differences (*p* = 0.05) in the prevalence of hypomagnesemia between the 105 people who developed neuropathy (*n* = 17; 16.2%) compared to the 25 people who did not (*n* = 1; 4%). 

In a retrospective study of 169 stage III colorectal cancer patients receiving oxaliplatin-based chemotherapy, pre-treatment hypomagnesemia was present in 80% of persons who developed grade 2–3 neuropathy, compared to only 20% of those who only developed grade 0–1 neuropathy (*p* = 0.0001) [15]. The overall percentage of participants with pre-treatment hypomagnesemia was 26% (*n* = 44).

Among 186 participants receiving oxaliplatin-based chemotherapy, lower magnesium levels among participants with grade 2–3 CIPN compared to grade 0–1 were reported (1.93 ± 0.4 vs. 2.06 ± 0.2, *p*= 0.04) [19]. In multiple regression, magnesium levels were not an independent predictor of CIPN. 

In a prospective study that included 113 persons receiving oxaliplatin, cisplatin, paclitaxel, or docetaxel, no significant relationships between CIPN and magnesium levels were identified. This may have been due to the heterogeneity of chemotherapy drugs included in the study. As a whole, these mixed findings suggest that magnesium levels are associated with CIPN caused by oxaliplatin but may not be associated with CIPN caused by other neurotoxic agents. 

#### 3.4.4. Albumin and Prealbumin

Four studies evaluating the relationship between hypoalbuminemia and CIPN were identified with three demonstrating a significant relationship and one that did not demonstrate a relationship between CIPN and albumin. One was in breast cancer patients receiving paclitaxel [11] two in colorectal cancer patients receiving oxaliplatin [15,19], one in gastrointestinal patients receiving oxaliplatin, and one in lymphoma patients receiving vincristine. As previously described, among women with breast cancer who received paclitaxel, lower albumin (*p* = 0.002) was significantly associated with worsening neuropathy on the revised Total Neuropathy Score reduced version (rTNS). The mean albumin level was 4.26 g/dl (range 3.4–4.8) and only 1.6% (*n* = 1) had an abnormal albumin level in that study [11]. In a study of persons with colorectal cancer receiving oxaliplatin, pre-treatment hypoalbuminemia was present in 61% of persons who developed grade 2–3 neuropathy, compared to 39% of those who only developed grade 0–1 neuropathy (*p* = 0.01) [15]. The overall percentage of participants with pre-treatment hypoalbuminemia (less than 3.5 gr/dL) was 80% (*n* = 136).

In a second study of persons with colorectal cancer receiving oxaliplatin, albumin levels were lower in the group that developed grade 2–3 neuropathy, compared to those that developed grade 0–1 (3.9 ± 0.3 vs. 4.1 ± 0.4, *p* = 0.001) [19]. In multiple regression, albumin was not found to be an independent predictor of CIPN. Sensitivity and specificity evaluation determined that with a cut-off of 4.05 serum albumin was 51.4 sensitive and 77.8 specific (area under the curve = 66, *p* = 0.002). In contrast to these findings, among 40 persons receiving vincristine, none of the patients who developed grade 2–3 CIPN had low albumin levels prior to chemotherapy, compared to 37% who developed grade 0–1 CIPN, although the difference was not statistically significant (*p* = 0.06) [17]. 

Pre-albumin was evaluated in relationship to CIPN in two included studies, one that demonstrated a decrease in pre-albumin levels during taxane- or platinum-based chemotherapy among participants who developed severe CIPN and did not recover during the study [20] and another among persons receiving taxane in which pre-albumin was not significantly associated with development of CIPN [11]. 

#### 3.4.5. Glucose/Glycosylated Hemoglobin 

Four studies [11,13,17,19] evaluated the relationship between glucose levels or glycosylated hemoglobin and CIPN, and one of those reported a statistically significant relationship [19]. In that study of patients receiving oxaliplatin, serum glucose was higher among those who developed more severe neuropathy (*p* = 0.007).

#### 3.4.6. Vitamin E

Two studies evaluated a relationship between vitamin E levels and CIPN [11,20] and one reported that patients with more severe CIPN that did not experience recovery during the study period had a decrease in vitamin E levels compared to those who improved (*p* = 0.02) [20]. 

## 4. Discussion

Current evidence supporting a relationship between laboratory evidence of nutritional deficiency and CIPN is thus far inconclusive but suggests that anemia, hypoalbuminemia, hypomagnesemia, and low vitamin D levels could potentially contribute to development of CIPN. Well-designed, prospective studies are needed to further evaluate the strength of these relationships, as well as in which types of chemotherapy each of these relationships exist. Methodology and “cut off” values for variables such as hemoglobin, albumin, vitamin D, and magnesium are not consistent across studies or in clinical practice making the interpretation of study results challenging. Further, the evidence thus far at best suggests a prognostic, and not predictive, role of anemia, hypomagnesemia, low albumin, and low vitamin D, in CIPN development. 

Five studies included evaluation of vitamin B12 yet no association between vitamin B12 levels and CIPN were reported [10,11,15,19,20]. Solomon et al. demonstrated that functional B12 deficiency, with normal B12 levels, was associated with peripheral neuropathy among patients with advanced cancer [22]. Future studies should evaluate functional B12 deficiency in relationship to CIPN.

Limitations of the current evidence surrounding nutritional deficiencies as a contributing factor in development of CIPN include small sample sizes, retrospective and observational study designs, inconsistent measurement of CIPN across studies, and lack of longitudinal data. Although we conducted an extensive literature search, it is possible that relevant studies were not identified [23]. Further, our conclusions may be influenced by publication bias, as negative studies are less likely to be published than studies reporting positive findings [24]. Our decision to limit inclusion to nutritional factors that can be measured with a laboratory test limits our ability to thoroughly assess other aspects of nutrition such as obesity. Several studies have recently demonstrated a relationship between obesity and CIPN [25,26], yet these studies were not included in this review. 

Most of the studies included in this review were conducted outside of the United States (US) and those conducted within the US had fairly low proportion of unrepresented minorities such as African American, Asians, and Hispanic groups, therefore conclusions about whether these findings are consistent across racial and ethnic groups cannot be determined. Minority populations may be socioeconomically disadvantaged and have less access to nutritious food. Further, racial, and ethnic minorities often have higher rates of comorbidities including diabetes, that predispose them to neuropathy and may lead to earlier development or development of more severe CIPN [27,28]. Conversely, a meta-analysis of Asian and Western studies of colorectal cancer patients receiving oxaliplatin-based chemotherapy concluded that Asians may be less susceptible to oxaliplatin induced neurotoxicity than Caucasians, which could be attributable to genetic polymorphisms, cultural or lifestyle differences, including nutrition [29]. Prospective studies with larger cohorts of minority populations are needed to understand the role of nutrition in development of CIPN across all races and ethnicities. 

Prospective studies that compare relationships among nutritional factors and CIPN among different neurotoxic chemotherapy drug classes are also needed. For instance, our review demonstrated that low magnesium levels are associated with CIPN among colorectal cancer patients treated with oxaliplatin but no studies evaluating this relationship among persons receiving taxanes or other neurotoxic chemotherapies were included in this review. Research is needed to identify whether low magnesium is associated with CIPN across neurotoxic drug classes. Future studies also should control for neuropathy associated co-morbidities (BMI, history of alcohol use, etc.) to better understand which factors are independently associated with CIPN so that risk modeling strategies can be developed. 

Although we did not include interventional studies in this review, data supporting the role of replacement strategies such as vitamin B12 or vitamin D replacement to prevent or ameliorate CIPN are extremely limited [4,30]. A systematic review performed in 694 patients from 5 trials (published 2010–2014) confirmed that there was no beneficial effect in terms of the incidence of grade ≥ 2 neuropathy) or chronic neurotoxicity (RR, 0.95; 95% CI, 0.69–1.32) from calcium and magnesium infusions for the prevention of oxaliplatin-induced peripheral neuropathy [4,30]. Further, current guidelines from the American Society of Clinical Oncology report that there is insufficient evidence to recommend any nutritional supplement for prevention or treatment of CIPN [4]. Given the dearth of effective therapies to prevent CIPN, ongoing optimization of clinical studies to assess the prognostic and predictive role of nutritional deficiencies is required. Well-designed clinical trials to further determine if replacement/supplementation of nutritional deficiencies will help decrease the incidence and severity of CIPN are needed. Given that the laboratory tests discussed here are often part of routine oncology care, reviewing these data prior to chemotherapy may help inform the assessment of risk of CIPN development.

## Figures and Tables

**Table 1:** Summary of included studies.

Authors	Drug	Cancer Diagnosis	Neuropathy Measure	Percentage of Sample with Neuropathy
Grim et al. [12]	Paclitaxel	Breast	Michigan Neuropathy Screening Instrument	60% ^1^
Jennaro et al. [10]	Paclitaxel	Breast	EORTC CIPN-20 ^5^	32.4%
Ottaiano et al. [13]	Oxaliplatin	Colorectal	CTCAE ^6^	17.7%
Robertson et al. [11]	Paclitaxel	Breast	TNS ^7^	42%
Saito et al. [17]	Vincristine	Lymphoma	CTCAE	17.5% ^2^
Shahriari-Ahmadi et al. [14]	Oxaliplatin	Colorectal	CTCAE	80.7%
Velasco et al. [20]	Platinum or taxane	Colorectal, gastric, lung, or breast	TNS CTCAE Nerve conduction studies	52% ^4^
Vincenzi et al. [15]	Oxaliplatin	Colorectal	CTCAE	40%
Wang et al. [18]	Thalidomide/velcade	Multiple myeloma	CTCAE FACT/GOG-NTX ^8^	59%
Winkels et al. [16]	Oxaliplatin/ capecitabine	Colorectal	CIPN20	81%
Yildirim et al. [19]	Oxaliplatin	Gastrointestinal (colorectal, gastric, or pancreatic)	CTCAE	42% ^2,3^

^1^ Listed as CIPN complications; ^2^ grade 2–3 CIPN; ^3^ 58% had grade 0–1 CIPN; ^4^ the overall percentage of people who developed neuropathy (grade > 0) was higher at 88% for those receiving platinum and 79% for those receiving paclitaxel; ^5^ European Organization for Research and Treatment of Cancer Quality of Life Questionnaire-CIPN twenty-item scale; ^6^ Common Terminology Criteria for Adverse Events; ^7^ Total Neuropathy Score; ^8^ Functional Assessment of Cancer Therapy Scale/Gynecologic Oncology Group-Neurotoxicity.

**Table 2:** Racial and ethnic minority representation.

Reference	Country	Sample Size	Frequency (Percent) of Minority Race or Ethnicity	African American/Black ^1^	Hispanic ^1^	Asian ^1^	Other ^1^
Wang et al. [18]	USA	109	21 (19.3)	9 (8.3)	5 (4.6)	3 (2.8)	4 (3.7)
Robertson et al. [11]	USA	61	12 (19.1)	7 (11.5)	2 (3.3)	3 (4.9)	0
Jennaro et al. [10]	USA	37	3 (8.1)	Race/ethnicity only described as white or non-white. No non-white participants were in the vitamin D-deficient group.			
Vincenzi et al. [15]	Italy	169	NR ^1^	Race/ethnicity not described.			
Ottaiano et al. [13]	Italy	102	NR	Race/ethnicity not described.			
Shahriari-Ahmadi et al. [14]	Iran	130	NR	Race/ethnicity not described.			
Saito et al. [17]	Japan	40	NR	Race/ethnicity not described.			
Grim et al. [12]	Czech Republic	70	NR	Race/ethnicity not described.			
Velasco et al. [20]	Spain	113	NR	Race/ethnicity not described.			
Winkels et al. [16]	Netherlands	159	NR	Race/ethnicity not described.			
Yildirim et al. [19]	Turkey	186	NR	Race/ethnicity not described.			

NR = not reported. ^1^ Reported as frequency with percentage in parentheses.

**Table 3:** Comorbid conditions reported in each study.

	Vincenzi et al. [15]	Wang et al. [18]	Jennaro et al. [10]	Robertson et al. [11]	Shahriari-Ahmadi et al. [14]	Saito et al. [17]	Ottaiano et al. [13]	Grim et al. [12]	Velasco et al. [20]	Winkels et al. [16]	Yildirim et al. [19]
Sample size (demographic data)	169	109	37	61	130	40	102	70	113	159	186
Diabetes/hyperglycemia ^2^	29 (17.2)	11 (10)	8 (21.6)	4 (6.6)	28 (21.5)	7 (17.5)	19 (18.7)	NR ^1^ (9.5)	18 (16)	NR	NR
Renal failure ^2^	15 (8.9)	NR	NR	NR	NR	5 (12.5)	NR	NR	NR	NR	NR
Hepatic dysfunction ^2^	NR	NR	NR	NR	NR	10 (25)	NR	NR	NR	NR	NR
Alcohol use ^2^	13 (7.7)	NR	19 (51.4)	38 (62.3)	5 (3.8)	NR	NR	NR	NR	NR	NR
Tobacco use ^2^	NR	NR	NR	NR	NR	NR	66 (64.7)	NR	NR	NR	NR
Malnutrition ^2^	NR	NR	NR	NR	NR	12 (30)	NR	NR	NR	NR	NR
Overweight/obese ^2^	NR	NR	NR	NR	NR	NR	36 (35.3)	NR	NR	NR	NR
Hypertension ^2^	NR	NR	NR	14 (22.9)	NR	NR	21 (20.6)	NR	NR	NR	36 (19.4)
Electrolyte imbalance ^2^	NR	NR	NR	NR	NR	4 (10)	NR	NR	NR	NR	NR
High cholesterol ^2^	NR	NR	NR	NR	NR	NR	23 (22.5)	NR	NR	NR	NR
Hyperlipidemia ^2^	NR	NR	NR	13 (21.3)	NR	NR	NR	NR	NR	NR	NR
High triglycerides ^2^	NR	NR	NR	NR	NR	NR	21 (20.6)	NR	NR	NR	NR
Hypocalcemia	34 (20)	NR	NR	NR	25 (19.3)	NR	NR	NR	NR	NR	NR
Coronary artery disease	NR	NR	NR	NR	NR	NR	NR	NR	NR	NR	12 (6.5)
Dyslipidemia	NR	NR	NR	NR	NR	NR	NR	NR	NR	NR	14 (7.5)

^1^ Total number of patients was not reported for diabetes, only percentage. NR = not reported. ^2^ Reported as frequency with percentage in parentheses.

**Table 4:** Nutritional lab correlates.

Author	Nutritional Lab Measures Evaluated	Lab Measures Associated with CIPN
Grim et al. [12]	Vitamin B1 Vitamin B6 Omega 3 fatty acids Vitamin D	Vitamin D
Jennaro et al. [10]	Vitamin D Vitamin B12 Folate Homocysteine	Vitamin D
Ottaiano et al. [13]	Triglycerides Cholesterol HbA1c	None
Robertson et al. [11]	HbA1c Vitamin B12 Vitamin B6 Vitamin E Folate Lipid panel Albumin Prealbumin	Albumin
Saito et al. [17]	Glucose Albumin Hemoglobin Potassium	Hemoglobin
Shahriari-Ahmadi et al. [14]	Calcium Magnesium	Hemoglobin Magnesium
Velasco et al. [20]	Magnesium Vitamin B12 Prealbumin Vitamin E	Vitamin E Pre-albumin
Vincenzi et al. [15]	Calcium Magnesium Albumin Hemoglobin Folate Vitamin B12	Hemoglobin Albumin Magnesium
Wang et al. [18]	Vitamin D	Vitamin D
Winkels et al. [16]	Ergothioneine	None
Yildirim et al. [19]	Blood glucose Vitamin D Hemoglobin Albumin Magnesium Triglycerides Calcium Sodium Potassium Vitamin B12 Folic acid	Blood glucose Vitamin D Hemoglobin Albumin Magnesium

## Supplementary Materials

The following are available online at https://www.mdpi.com/article/10.3390/jcm11020355/s1, Search strategies.
