# Supplementary material for: A Systematic Review of Nutritional Lab Correlates with Chemotherapy Induced Peripheral Neuropathy
Source: J Clin Med. 2022 Jan 12;11(2):355. doi: 10.3390/jcm11020355 (PMC8780075; doi:10.3390/jcm11020355)
Supplement: Supplementary file 1 [file jcm-11-00355-s001.zip › jcm-1481100-supplementary.pdf]

Title/ Topic: Relationships Between Laboratory Measures of Nutrition and Chemotherapy-Induced Peripheral Neuropathy.

Requesters/ Authors: Cindy Tofthagen, Ph.D

The first round of searching was completed on June 04 or June 05, 2020. An updated search was run on December 08, 2021.

Databases searched with number of original references and number of references after de-duplication.

Database	Number of initial hits [06/04/2020]	Number after de-duplication [06/04/2020]	Number of hits [12/08/2021]	Number after de-duplication [12/08/2021]	Number after de-duplication with 06/04/2021 citation set [12/08/2021]
1. Ovid MEDLINE	343	343	53	53	38
2. Ovid EMBASE	614	542	103	91	79
3. Ovid Cochrane Central Register of Controlled Trials (CCRCT)	26	10	5	2	2
4. Ovid Cochrane Database of Systematic Review (CDSR)	0	0	1	1	1
5. Scopus	270	236	33	31	30
6. Web of Science - Science Citation Index Expanded (SCI-EXPANDED)	77	37	12	4	3
7. Web of Science - Emerging Sources Citation Index (ESCI)	4	4	1	0	0
8. EBSCO - Cumulative Index to Nursing and Allied Health Literature (CINAHL)	164	103	20	14	14
9. Epistemonikos	17	16	24	21	4
Grey Literature<br><br>10. ClinicalTrials.gov<br>11. medRxiv	13<br><br>- *10*<br>- *0*	10<br><br>- *0*<br>- *0*	10<br><br>- *6*<br>- *2*	9<br><br>- *6*<br>- *2*	9<br><br>- *6*<br>- *2*

© Mayo Clinic Libraries, Mayo Foundation for Medical Education and Research

12. NICE Evidence<br>13. OpenGrey<br>14. PROSPERO	- *3*<br>- *0*<br>- *0*	- *0*<br>- *0*<br>- *0*	- *1*<br>- *0*<br>- *1*	- *0*<br>- *0*<br>- *1*	- *0*<br>- *0*<br>- *1*
Total	***1528***	***1301***	***262***	***226***	***180***

Complete search strategy for each database and grey literature resources for the appendix.

***1.* <u>Ovid MEDLINE(R)</u>** 1946 to Present and Epub Ahead of Print, In-Process & Other Non-Indexed Citations and Ovid MEDLINE(R) Daily – searched June 4, 2020.

Update: Ovid **<u>MEDLINE</u>**(R) 1946 to Present and Epub Ahead of Print, In-Process & Other Non-Indexed Citations and Ovid MEDLINE(R) Daily – date range searched: January 01, 2020 – current (searched on December 08, 2021).

1. exp Peripheral Nervous System Diseases/ci [Chemically Induced]

2. exp Neuralgia/ci

3. 1 or 2

4. Peripheral Nervous System Diseases/

5. ((peripheral adj2 (nerve* or nervous) adj2 (disease* or disorder*)) or "peripheral neuropath*").ti,ab,oa,kw.

6. (PNS adj2 (disease* or disorder*)).ti,ab,oa,kw.

7. 4 or 5 or 6

8. exp Antineoplastic Agents/ or Antineoplastic Combined Chemotherapy Protocols/ or Chemotherapy, Adjuvant/ or (antineoplastic or chemotherap* or chemo-therap* or ((anticancer* or anti-cancer* or anticarcino* or anti-carcino* or antitumor or anti-tumor or antitumour* or anti-tumour* or carcinostatic) adj2 (therap* or agent or agents or treatment* or management*)) or carcinochemo* or "tumor inhibitor" or "tumour inhibitor").ti,ab,oa,kw.

9. ("paclitaxel" or "abi 007" or "abi007" or "abraxane" or "albumin bound paclitaxel" or "anzatax" or "apealea" or "asotax" or "biotax" or "bms 181339" or "bms181339" or "bmy 45622" or "bmy45622" or "bristaxol" or "britaxol" or "coroxane" or "dts 301" or "dts301" or "endotag-1" or "formoxol" or "genexol" or "genexol pm" or "hunxol" or "ifaxol" or "infinnium" or "intaxel" or "mbt 0206" or "mbt0206" or "medixel" or "mitotax" or "nab paclitaxel" or "nanoparticle albumin bound paclitaxel" or "nsc 125973" or "nsc 673089" or "nsc125973" or "nsc673089" or "oas pac 100" or "oaspac100" or "oncogel" or "onxol" or "pacitaxel" or "paclitaxel nab" or "pacxel" or "padexol" or "parexel" or "paxceed" or "paxene" or "paxus" or "praxel" or "taxocris" or "taxol" or "taycovit" or "yewtaxan").ti,ab,sh.

10. ("docetaxel" or "daxotel" or "dexotel" or "docefrez" or "docetaxel accord" or "lit 976" or "lit976" or "n debenzoyl n tert butoxycarbonyl 10 deacetyltaxol" or "n tert butoxycarbonyl 10 deacetyl n

© Mayo Clinic Libraries, Mayo Foundation for Medical Education and Research

debenzoyltaxol" or "nsc 628503" or "nsc628503" or "oncodocel" or "rp 56976" or "rp56976" or "taxespira" or "taxoter" or "taxotere" or "texot").ti,ab,sh.

11. ("oxaliplatin" or "axiplatin" or "crisapla" or "croloxat" or "dacotin" or "dacplat" or "ebeoxal" or "elatofen" or "eloxatin" or "eloxatine" or "elplat" or "geneplatin" or "heloxatin" or "lipoxal" or "mbp 426" or "mbp426" or "medoxa" or "oplat" or "oxalatoplatinum" or "oxalatplatin" or "oxali" or "oxalip" or "oxaliplan" or "oxaliplatina" or "oxaliprol" or "oxaliquid" or "oxalisan" or "oxalisin" or "oxalizor" or "oxaltic" or "oxaltina" or "oxaplamyl" or "oxaviatin" or "platox" or "plaxitin" or "rectoxal" or "riboxatin" or "rp 54780" or "rp54780" or "sinoxal" or "sr 96669" or "sr96669" or "transplastin" or "velminox" or "xaliplat" or "xoplan").ti,ab,sh.

12. ("cisplatin" or "abiplatin" or "biocisplatinum" or "biocysplatinum" or "blastolem" or "briplatin" or "cddp ti" or "cis ddp" or "cis diamine dichloroplatinum" or "cis diaminechloroplatinum" or "cis diaminedichloroplatinum" or "cis diammine dichloroplatinum" or "cis diamminedichloroplatinum" or "cis dichloridiammineplatinum" or "cis dichloroadiamine platinum" or "cis dichlorodiamine platinum" or "cis dichlorodiammineplatinum" or "cis platinous diamino dichloride" or "cis platinum" or "cis platinum diamine dichloride" or "cis platinum diaminedichloride" or "cis platinum diamino dichloride" or "cis platinum diaminochloride" or "cis platinum diaminodichloride" or "cis platinum diammine dichloride" or "cis platinum diamminedichloride" or "cisplatin liposomal" or "cisplatin therapeutic implant" or "cisplatin-ebewe" or "cisplatine" or "cisplatino" or "cisplatinum" or "cisplatyl" or "citoplatino" or "cytoplatin" or "cytosplat" or "diamine dichloroplatinum" or "diaminodichloroplatinum" or "diamminedichloroplatinum" or "dichlorodiamine platinum" or "dichlorodiammineplatinum" or "docistin" or "elvecis" or "kemoplat" or "lederplatin" or "lipoplatin" or "liposomal cisplatin" or "mpi 5010" or "mpi5010" or "neoplatin" or "niyaplat" or "nk 801" or "noveldexis" or "nsc 119875" or "platamine" or "platamine rtu" or "platiblastin" or "platidiam" or "platimine" or "platinex" or "platinil" or "platinol" or "platinol aq" or "platinoxan" or "platinum diamine dichloride" or "platinum diaminedichloride" or "platinum diaminodichloride" or "platinum diamminedichloride" or "platiran" or "platistil" or "platistin" or "platosin" or "randa" or "romcis" or "sicatem" or "spi 077" or "tecnoplatin").ti,ab,sh.

13. ("bortezomib" or "ldp 341" or "ldp341" or "mg 341" or "mg341" or "mln 341" or "mln341" or "ps 341" or "ps341" or "velcade" or "vincristine" or "l 37231" or "l37231" or "vin cristine" or "vincristin" or "vincrisul" or "vinblastine" or "le 29060" or "le29060" or "leukoblastin" or "rozevin" or "vin blastine" or "vinblastin" or "vincaleucoblastin" or "vincaleucoblastine" or "vincaleukoblastine" or "vincoblastin" or "vincoblastine" or "vincoleucoblastine" or "vincoleukoblastin" or "vinleucoblastine").ti,ab,sh.

14. ("thalidomide" or "beta thalidomide" or "contergan" or "distaval" or "isomin" or "k 17" or "kedavon" or "kevadon" or "neurosedin" or "neurosedyne" or "nsc 66847" or "sedalis" or "shin naito" or "softenon" or "synovir" or "talimol" or "talizer" or "telagan" or "telargan" or "thado" or "thalidomid" or "thalidomide celgene" or "thalimodide" or "thalix" or "thalomid").ti,ab,sh.

15. or/8-14

16. 7 and 15

17. ((chemo* or antineoplas* or anti-neoplas* or anticancer* or anti-cancer* or anticarcino* or anti-carcino* or antitumor or anti-tumor or antitumour or anti-tumour or carcinochemo* or carcinostatic or "tumor inhibitor" or "tumour inhibitor") adj3 (neuropath* or neuralgia or neurotox* or mononeuropath* or mono-neuropath* or polyneuropath* or poly-neuropath*)).ti,ab,oa,kw.

18. 3 or 16 or 17

19. exp Clinical Laboratory Techniques/ or Clinical Laboratory Services/ or exp Laboratories/

© Mayo Clinic Libraries, Mayo Foundation for Medical Education and Research

20. ((laboratory adj2 (marker or markers or values or test* or technique* or service or services or diagnos* or examin* or evaluat*)) or ("lab test" or "lab tests" or "lab result" or "lab results" or "lab value" or "lab values" or deficien*)).ti,ab,oa,kw.

21. 19 or 20

22. exp Hemoglobins/ or Lipotropic Agents/ or exp Anemia/ or Methylmalonic Acid/ or Malonates/ or exp Homocysteine/ or exp Nutrients/ or Minerals/ or Vitamin A/ or Thiamine/ or Riboflavin/ or Niacinamide/ or Niacin/ or Pantothenic Acid/ or Vitamin B 6/ or Biotin/ or Folic Acid/ or Vitamin B 12/ or Ascorbic Acid/ or Vitamin D/ or Vitamin E/ or Vitamin K/ or Prothrombin Time/ or Calcium/ or Calcium, Dietary/ or Magnesium/ or Phosphorus, Dietary/ or Potassium/ or Potassium, Dietary/ or Sodium, Dietary/ or Chromium/ or Copper/ or Fluorides/ or Iodine/ or Iron/ or Iron, Dietary/ or Manganese/ or Molybdenum/ or Selenium/ or Zinc/

23. (hemoglobin or hemoglobins or haemoglobin or hemoglobine or haemoglobine or haemoglobins or hemoglobulin or hematocrit or ferrohaemoglobin or ferrohemoglobin or erythrocytes or eryhem or albumin or albumen or prealbumin or pre-albumin or total-protein or leptin or globulin or globulins or polyglobulin or poly-globulin or choline or bursine or fagine or vidine or thiamin or thiamine or anemi* or anaemi* or aneurin or riboflavin or riboflavine or "flavin mononucleotide" or "flavin-adenine dinucleotide" or niacin or niacinamide or enduramide or nicobion or nicotinamide or nicotinsaureamid or papulex or "pantothenic acid" or "calcium pantothenate" or dexol or "zinc pantothenate" or "pyridoxal phosphate" or "pyridoxal 5-phosphate" or "pyridoxal-P" or biotin or folate or "folic acid" or folacin or folvite or folvit or "pteroylglutamic acid" or cobalamin or cobalamins or cyanocobalamin or eritron or "B1" or "B2" or "B3" or "B5" or "B6" or "B7" or "B9" or "B12" or (B adj ("1" or "2" or "3" or "5" or "6" or "7" or "9" or "12")) or "methylmalonic acid" or "methyl malonate" or "methyl malonic acid" or methylmalonate or retinol or carotene or "Aquasol A" or phytomenadione or menaquinone or menadione or calcium or chloride or magnesium or phosphorus or romag or potassium or kalium or sodium or chromium or copper or fluoride or iodine or iron or "Fe" or "Fe+++" or ferro or ferrum or manganese or molybdenum or selenium or zinc).ti,ab,oa,kw.

24. 22 or 23

25. 21 and 24

26. ((("complete blood" or "red blood cell" or erythrocyte or RBC or iron or reticulocyte) adj1 (count or indices)) or "CBC" or "hemogram" or (("coronary risk" or lipid or cholesterol or prothrombin or prothrombine or protrombin) adj1 (panel or test)) or Thrombotest or "Fe Tests").ti,ab,oa,kw.

27. (exp "Nutritional and Metabolic Diseases"/ not exp Overnutrition/) or Hypercalciuria/

28. (undernutrition* or malnutrition* or avitaminosis or ((nutrition* or mineral* or metabolic or nutrient* or micronutrient* or micro-nutrient*) adj2 (deficien* or diseases or disorder*))).ti,ab,oa,kw.

29. (beriberi or pellagra or scurvy or rickets or osteomalacia or hypocalcem* or hypo-calcem* or hypercalcem* or hyper-calcem* or hypocalcaem* or hypo-calcaem* or hypercalcaem* or hyper-calcaem* or hypocalciur* or hypo-calciur* or hypercalciur* or hyper-calciur* or hypomagnesem* or hypo-magnesem* or hypermagnesem* or hyper-magnesem* or hypomagnesaem* or hypo-magnesaem* or hypermagnesaem* or hyper-magnesaem* or hypoalbuminem* or hypo-albuminem* or hyperalbuminem* or hyper-albuminem* or hypoalbuminaem* or hypo-albuminaem* or hyperalbuminaem* or hyper-albuminaem* or hypokalem* or hypo-kalem* or hyperkalem* or hyper-kalem* or hypokalaem* or hypo-kalaem* or hyperkalaem* or hyper-kalaem* or hypohomocysteinem* or hypo-homocysteinem* or hyperhomocysteinem* or hyper-homocysteinem*).ti,ab,oa,kw.

30. 25 or 26 or 27 or 28 or 29

31. 18 and 30

© Mayo Clinic Libraries, Mayo Foundation for Medical Education and Research

32. 31 not (Animals/ not (Animals/ and Humans/))

33. case reports.pt. or (case adj1 (report* or series)).ti,ab.

34. 32 not 33

35. 34 not ((exp infant/ or exp child/ or adolescent/) not exp adult/)

36. 34 not (exp adult/ not (exp infant/ or exp child/ or adolescent/))

37. 34 not 36

38. 35 or 37

39. remove duplicates from 38

40. limit 39 to ed=20200101-20301231

41. limit 39 to dt=20200101-20301231

42. 40 or 41

2. **Ovid Embase** 1974 to 2020 June 03– searched June 4, 2020.

Update: Ovid **Embase** 1974 to 2021 December 07 – date range searched: January 01, 2020 – current (searched on December 08, 2021).

1. chemotherapy-induced peripheral neuropathy/

2. peripheral neuropathy/

3. ((peripheral adj2 (nerve* or nervous) adj2 (disease* or disorder*)) or "peripheral neuropath*").ti,ab.

4. (PNS adj2 (disease* or disorder*)).ti,ab.

5. 2 or 3 or 4

6. exp chemotherapy/ or antineoplastic agent/ or (antineoplastic or chemotherap* or chemo-therap* or ((anticancer* or anti-cancer* or anticarcino* or anti-carcino* or antitumor or anti-tumor or antitumour* or anti-tumour* or carcinostatic) adj2 (therap* or agent or agents or treatment* or management*)) or carcinochemo* or "tumor inhibitor" or "tumour inhibitor").ti,ab.

7. ("paclitaxel" or "abi 007" or "abi007" or "abraxane" or "albumin bound paclitaxel" or "anzatax" or "apealea" or "asotax" or "biotax" or "bms 181339" or "bms181339" or "bmy 45622" or "bmy45622" or "bristaxol" or "britaxol" or "coroxane" or "dts 301" or "dts301" or "endotag-1" or "formoxol" or "genexol" or "genexol pm" or "hunxol" or "ifaxol" or "infinnium" or "intaxel" or "mbt 0206" or "mbt0206" or "medixel" or "mitotax" or "nab paclitaxel" or "nanoparticle albumin bound paclitaxel" or "nsc 125973" or "nsc 673089" or "nsc125973" or "nsc673089" or "oas pac 100" or "oaspac100" or "oncogel" or "onxol" or "pacitaxel" or "paclitaxel nab" or "pacxel" or "padexol" or "parexel" or "paxceed" or "paxene" or "paxus" or "praxel" or "taxocris" or "taxol" or "taycovit" or "yewtaxan").ti,ab.

© Mayo Clinic Libraries, Mayo Foundation for Medical Education and Research

8. ("docetaxel" or "daxotel" or "dexotel" or "docefrez" or "docetaxel accord" or "lit 976" or "lit976" or "n debenzoyl n tert butoxycarbonyl 10 deacetyltaxol" or "n tert butoxycarbonyl 10 deacetyl n debenzoyltaxol" or "nsc 628503" or "nsc628503" or "oncodocel" or "rp 56976" or "rp56976" or "taxespira" or "taxoter" or "taxotere" or "texot").ti,ab.

9. ("oxaliplatin" or "axiplatin" or "crisapla" or "croloxat" or "dacotin" or "dacplat" or "ebeoxal" or "elatofen" or "eloxatin" or "eloxatine" or "elplat" or "geneplatin" or "heloxatin" or "lipoxal" or "mbp 426" or "mbp426" or "medoxa" or "oplat" or "oxalatoplatinum" or "oxalatplatin" or "oxali" or "oxalip" or "oxaliplan" or "oxaliplatina" or "oxaliprol" or "oxaliquid" or "oxalisan" or "oxalisin" or "oxalizor" or "oxaltic" or "oxaltina" or "oxaplamyl" or "oxaviatin" or "platox" or "plaxitin" or "rectoxal" or "riboxatin" or "rp 54780" or "rp54780" or "sinoxal" or "sr 96669" or "sr96669" or "transplastin" or "velminox" or "xaliplat" or "xoplan").ti,ab.

10. ("cisplatin" or "abiplatin" or "biocisplatinum" or "biocysplatinum" or "blastolem" or "briplatin" or "cddp ti" or "cis ddp" or "cis diamine dichloroplatinum" or "cis diaminechloroplatinum" or "cis diaminedichloroplatinum" or "cis diammine dichloroplatinum" or "cis diamminedichloroplatinum" or "cis dichloridiammineplatinum" or "cis dichloroadiamine platinum" or "cis dichlorodiamine platinum" or "cis dichlorodiammineplatinum" or "cis platinous diamino dichloride" or "cis platinum" or "cis platinum diamine dichloride" or "cis platinum diaminedichloride" or "cis platinum diamino dichloride" or "cis platinum diaminochloride" or "cis platinum diaminodichloride" or "cis platinum diammine dichloride" or "cis platinum diamminedichloride" or "cisplatin liposomal" or "cisplatin therapeutic implant" or "cisplatin-ebewe" or "cisplatine" or "cisplatino" or "cisplatinum" or "cisplatyl" or "citoplatino" or "cytoplatin" or "cytosplat" or "diamine dichloroplatinum" or "diaminodichloroplatinum" or "diamminedichloroplatinum" or "dichlorodiamine platinum" or "dichlorodiammineplatinum" or "docistin" or "elvecis" or "kemoplat" or "lederplatin" or "lipoplatin" or "liposomal cisplatin" or "mpi 5010" or "mpi5010" or "neoplatin" or "niyaplat" or "nk 801" or "noveldexis" or "nsc 119875" or "platamine" or "platamine rtu" or "platiblastin" or "platidiam" or "platimine" or "platinex" or "platinil" or "platinol" or "platinol aq" or "platinoxan" or "platinum diamine dichloride" or "platinum diaminedichloride" or "platinum diaminodichloride" or "platinum diamminedichloride" or "platiran" or "platistil" or "platistin" or "platosin" or "randa" or "romcis" or "sicatem" or "spi 077" or "tecnoplatin").ti,ab.

11. ("bortezomib" or "ldp 341" or "ldp341" or "mg 341" or "mg341" or "mln 341" or "mln341" or "ps 341" or "ps341" or "velcade" or "vincristine" or "l 37231" or "l37231" or "vin cristine" or "vincristin" or "vincrisul" or "vinblastine" or "le 29060" or "le29060" or "leukoblastin" or "rozevin" or "vin blastine" or "vinblastin" or "vincaleucoblastin" or "vincaleucoblastine" or "vincaleukoblastine" or "vincoblastin" or "vincoblastine" or "vincoleucoblastine" or "vincoleukoblastin" or "vinleucoblastine").ti,ab.

12. ("thalidomide" or "beta thalidomide" or "contergan" or "distaval" or "isomin" or "k 17" or "kedavon" or "kevadon" or "neurosedin" or "neurosedyne" or "nsc 66847" or "sedalis" or "shin naito" or "softenon" or "synovir" or "talimol" or "talizer" or "telagan" or "telargan" or "thado" or "thalidomid" or "thalidomide celgene" or "thalimodide" or "thalix" or "thalomid").ti,ab.

13. 6 or 7 or 8 or 9 or 10 or 11 or 12

14. 5 and 13

© Mayo Clinic Libraries, Mayo Foundation for Medical Education and Research

15. ((chemo* or antineoplas* or anti-neoplas* or anticancer* or anti-cancer* or anticarcino* or anti-carcino* or antitumor or anti-tumor or antitumour or anti-tumour or carcinochemo* or carcinostatic or "tumor inhibitor" or "tumour inhibitor") adj3 (neuropath* or neuralgia or neurotox* or mononeuropath* or mono-neuropath* or polyneuropath* or poly-neuropath*)).ti,ab.

16. 1 or 14 or 15

17. exp *laboratory technique/ or *clinical laboratory service/ or exp *laboratory diagnosis/

18. ((laboratory adj2 (marker or markers or values or test* or technique* or service or services or diagnos* or examin* or evaluat*)) or ("lab test" or "lab tests" or "lab result" or "lab results" or "lab value" or "lab values" or deficien*)).ti,ab.

19. 17 or 18

20. exp *hemoglobin/ or *lipotropic agent/ or exp *anemia/ or *methylmalonic acid/ or *malonic acid derivative/ or *homocysteine/ or exp *nutrient/ or *mineral/ or exp *carotenoid/ or *thiamine/ or *riboflavin/ or *nicotinamide/ or *nicotinic acid/ or *pantothenic acid/ or *pyridoxine/ or *biotin/ or *folic acid/ or *cyanocobalamin/ or exp *ascorbic acid/ or exp *vitamin D/ or *prothrombin time/ or *calcium/ or *magnesium/ or *phosphorus/ or *potassium/ or *sodium/ or *chromium/ or *copper/ or *fluoride/ or *iodine/ or *iron/ or *manganese/ or *molybdenum/ or *selenium/ or *zinc/

21. (hemoglobin or hemoglobins or haemoglobin or hemoglobine or haemoglobine or haemoglobins or hemoglobulin or hematocrit or ferrohaemoglobin or ferrohemoglobin or erythrocytes or eryhem or albumin or albumen or prealbumin or pre-albumin or total-protein or leptin or globulin or globulins or polyglobulin or poly-globulin or choline or bursine or fagine or vidine or thiamin or thiamine or anemi* or anaemi* or aneurin or riboflavin or riboflavine or "flavin mononucleotide" or "flavin-adenine dinucleotide" or niacin or niacinamide or enduramide or nicobion or nicotinamide or nicotinsaureamid or papulex or "pantothenic acid" or "calcium pantothenate" or dexol or "zinc pantothenate" or "pyridoxal phosphate" or "pyridoxal 5-phosphate" or "pyridoxal-P" or biotin or folate or "folic acid" or folacin or folvite or folvit or "pteroylglutamic acid" or cobalamin or cobalamins or cyanocobalamin or eritron or "B1" or "B2" or "B3" or "B5" or "B6" or "B7" or "B9" or "B12" or (B adj ("1" or "2" or "3" or "5" or "6" or "7" or "9" or "12")) or "methylmalonic acid" or "methyl malonate" or "methyl malonic acid" or methylmalonate or retinol or carotene or "Aquasol A" or phytomenadione or menaquinone or menadione or calcium or chloride or magnesium or phosphorus or romag or potassium or kalium or sodium or chromium or copper or fluoride or iodine or iron or "Fe" or "Fe+++" or ferro or ferrum or manganese or molybdenum or selenium or zinc).ti,ab.

22. 20 or 21

23. 19 and 22

24. ((("complete blood" or "red blood cell" or erythrocyte or RBC or iron or reticulocyte) adj1 (count or indices)) or "CBC" or "hemogram" or (("coronary risk" or lipid or cholesterol or prothrombin or prothrombine or protrombin) adj1 (panel or test)) or Thrombotest or "Fe Tests").ti,ab.

25. exp *nutritional disorder/ or hypercalciuria/ or exp *metabolic disorder/

© Mayo Clinic Libraries, Mayo Foundation for Medical Education and Research

26. (undernutrition* or malnutrition* or avitaminosis or ((nutrition* or mineral* or metabolic or nutrient* or micronutrient* or micro-nutrient*) adj2 (deficien* or diseases or disorder*))).ti,ab.

27. (beriberi or pellagra or scurvy or rickets or osteomalacia or hypocalcem* or hypo-calcem* or hypercalcem* or hyper-calcem* or hypocalcaem* or hypo-calcaem* or hypercalcaem* or hyper-calcaem* or hypocalciur* or hypo-calciur* or hypercalciur* or hyper-calciur* or hypomagnesem* or hypo-magnesem* or hypermagnesem* or hyper-magnesem* or hypomagnesaem* or hypo-magnesaem* or hypermagnesaem* or hyper-magnesaem* or hypoalbuminem* or hypo-albuminem* or hyperalbuminem* or hyper-albuminem* or hypoalbuminaem* or hypo-albuminaem* or hyperalbuminaem* or hyper-albuminaem* or hypokalem* or hypo-kalem* or hyperkalem* or hyper-kalem* or hypokalaem* or hypo-kalaem* or hyperkalaem* or hyper-kalaem* or hypohomocysteinem* or hypo-homocysteinem* or hyperhomocysteinem* or hyper-homocysteinem*).ti,ab.

28. 23 or 24 or 25 or 26 or 27

29. 16 and 28

30. 29 not ((exp animal/ or nonhuman/) not exp human/)

31. 30 not (case adj1 (report* or series)).ti,ab.

32. 31 not (exp juvenile/ not exp adult/)

33. 31 not (exp adult/ not exp juvenile/)

34. 31 not 33

35. 32 or 34

36. remove duplicates from 35

37. limit 36 to dc=20200101-20301231

***3.* Ovid EBM Reviews - Cochrane Central Register of Controlled Trials** 1991 to May 2020 – searched June 4, 2020.

Update: Ovid EBM Reviews - **Cochrane Central Register of Controlled Trials** 1991 to November 2021 – date range searched: January 01, 2021 – current (searched on December 08, 2021).

1. ((peripheral adj2 (nerve* or nervous) adj2 (disease* or disorder*)) or "peripheral neuropath*").ti,ab.

2. (PNS adj2 (disease* or disorder*)).ti,ab.

3. 1 or 2

© Mayo Clinic Libraries, Mayo Foundation for Medical Education and Research

4. ("paclitaxel" or "abi 007" or "abi007" or "abraxane" or "albumin bound paclitaxel" or "anzatax" or "apealea" or "asotax" or "biotax" or "bms 181339" or "bms181339" or "bmy 45622" or "bmy45622" or "bristaxol" or "britaxol" or "coroxane" or "dts 301" or "dts301" or "endotag-1" or "formoxol" or "genexol" or "genexol pm" or "hunxol" or "ifaxol" or "infinnium" or "intaxel" or "mbt 0206" or "mbt0206" or "medixel" or "mitotax" or "nab paclitaxel" or "nanoparticle albumin bound paclitaxel" or "nsc 125973" or "nsc 673089" or "nsc125973" or "nsc673089" or "oas pac 100" or "oaspac100" or "oncogel" or "onxol" or "pacitaxel" or "paclitaxel nab" or "pacxel" or "padexol" or "parexel" or "paxceed" or "paxene" or "paxus" or "praxel" or "taxocris" or "taxol" or "taycovit" or "yewtaxan").ti,ab.

5. ("docetaxel" or "daxotel" or "dexotel" or "docefrez" or "docetaxel accord" or "lit 976" or "lit976" or "n debenzoyl n tert butoxycarbonyl 10 deacetyltaxol" or "n tert butoxycarbonyl 10 deacetyl n debenzoyltaxol" or "nsc 628503" or "nsc628503" or "oncodocel" or "rp 56976" or "rp56976" or "taxespira" or "taxoter" or "taxotere" or "texot").ti,ab.

6. ("oxaliplatin" or "axiplatin" or "crisapla" or "croloxat" or "dacotin" or "dacplat" or "ebeoxal" or "elatofen" or "eloxatin" or "eloxatine" or "elplat" or "geneplatin" or "heloxatin" or "lipoxal" or "mbp 426" or "mbp426" or "medoxa" or "oplat" or "oxalatoplatinum" or "oxalatplatin" or "oxali" or "oxalip" or "oxaliplan" or "oxaliplatina" or "oxaliprol" or "oxaliquid" or "oxalisan" or "oxalisin" or "oxalizor" or "oxaltic" or "oxaltina" or "oxaplamyl" or "oxaviatin" or "platox" or "plaxitin" or "rectoxal" or "riboxatin" or "rp 54780" or "rp54780" or "sinoxal" or "sr 96669" or "sr96669" or "transplastin" or "velminox" or "xaliplat" or "xoplan").ti,ab.

7. ("cisplatin" or "abiplatin" or "biocisplatinum" or "biocysplatinum" or "blastolem" or "briplatin" or "cddp ti" or "cis ddp" or "cis diamine dichloroplatinum" or "cis diaminechloroplatinum" or "cis diaminedichloroplatinum" or "cis diammine dichloroplatinum" or "cis diamminedichloroplatinum" or "cis dichloridiammineplatinum" or "cis dichloroadiamine platinum" or "cis dichlorodiamine platinum" or "cis dichlorodiammineplatinum" or "cis platinous diamino dichloride" or "cis platinum" or "cis platinum diamine dichloride" or "cis platinum diaminedichloride" or "cis platinum diamino dichloride" or "cis platinum diaminochloride" or "cis platinum diaminodichloride" or "cis platinum diammine dichloride" or "cis platinum diamminedichloride" or "cisplatin liposomal" or "cisplatin therapeutic implant" or "cisplatin-ebewe" or "cisplatine" or "cisplatino" or "cisplatinum" or "cisplatyl" or "citoplatino" or "cytoplatin" or "cytosplat" or "diamine dichloroplatinum" or "diaminodichloroplatinum" or "diamminedichloroplatinum" or "dichlorodiamine platinum" or "dichlorodiammineplatinum" or "docistin" or "elvecis" or "kemoplat" or "lederplatin" or "lipoplatin" or "liposomal cisplatin" or "mpi 5010" or "mpi5010" or "neoplatin" or "niyaplat" or "nk 801" or "noveldexis" or "nsc 119875" or "platamine" or "platamine rtu" or "platiblastin" or "platidiam" or "platimine" or "platinex" or "platinil" or "platinol" or "platinol aq" or "platinoxan" or "platinum diamine dichloride" or "platinum diaminedichloride" or "platinum diaminodichloride" or "platinum diamminedichloride" or "platiran" or "platistil" or "platistin" or "platosin" or "randa" or "romcis" or "sicatem" or "spi 077" or "tecnoplatin").ti,ab.

8. ("bortezomib" or "ldp 341" or "ldp341" or "mg 341" or "mg341" or "mln 341" or "mln341" or "ps 341" or "ps341" or "velcade" or "vincristine" or "l 37231" or "l37231" or "vin cristine" or "vincristin" or "vincrisul" or "vinblastine" or "le 29060" or "le29060" or "leukoblastin" or "rozevin" or "vin blastine" or

© Mayo Clinic Libraries, Mayo Foundation for Medical Education and Research

"vinblastin" or "vincaleucoblastin" or "vincaleucoblastine" or "vincaleukoblastine" or "vincoblastin" or "vincoblastine" or "vincoleucoblastine" or "vincoleukoblastin" or "vinleucoblastine").ti,ab.

9. ("thalidomide" or "beta thalidomide" or "contergan" or "distaval" or "isomin" or "k 17" or "kedavon" or "kevadon" or "neurosedin" or "neurosedyne" or "nsc 66847" or "sedalis" or "shin naito" or "softenon" or "synovir" or "talimol" or "talizer" or "telagan" or "telargan" or "thado" or "thalidomid" or "thalidomide celgene" or "thalimodide" or "thalix" or "thalomid").ti,ab.

10. (antineoplastic or chemotherap* or chemo-therap* or ((anticancer* or anti-cancer* or anticarcino* or anti-carcino* or antitumor or anti-tumor or antitumour* or anti-tumour* or carcinostatic) adj2 (therap* or agent or agents or treatment* or management*)) or carcinochemo* or "tumor inhibitor" or "tumour inhibitor").ti,ab.

11. 4 or 5 or 6 or 7 or 8 or 9 or 10

12. 3 and 11

13. ((chemo* or antineoplas* or anti-neoplas* or anticancer* or anti-cancer* or anticarcino* or anti-carcino* or antitumor or anti-tumor or antitumour or anti-tumour or carcinochemo* or carcinostatic or "tumor inhibitor" or "tumour inhibitor") adj3 (neuropath* or neuralgia or neurotox* or mononeuropath* or mono-neuropath* or polyneuropath* or poly-neuropath*)).ti,ab.

14. 12 or 13

15. ((laboratory adj2 (marker or markers or values or test* or technique* or service or services or diagnos* or examin* or evaluat*)) or ("lab test" or "lab tests" or "lab result" or "lab results" or "lab value" or "lab values" or deficien*)).ti,ab.

16. (hemoglobin or hemoglobins or haemoglobin or hemoglobine or haemoglobine or haemoglobins or hemoglobulin or hematocrit or ferrohaemoglobin or ferrohemoglobin or erythrocytes or eryhem or albumin or albumen or prealbumin or pre-albumin or total-protein or leptin or globulin or globulins or polyglobulin or poly-globulin or choline or bursine or fagine or vidine or thiamin or thiamine or anemi* or anaemi* or aneurin or riboflavin or riboflavine or "flavin mononucleotide" or "flavin-adenine dinucleotide" or niacin or niacinamide or enduramide or nicobion or nicotinamide or nicotinsaureamid or papulex or "pantothenic acid" or "calcium pantothenate" or dexol or "zinc pantothenate" or "pyridoxal phosphate" or "pyridoxal 5-phosphate" or "pyridoxal-P" or biotin or folate or "folic acid" or folacin or folvite or folvit or "pteroylglutamic acid" or cobalamin or cobalamins or cyanocobalamin or eritron or "B1" or "B2" or "B3" or "B5" or "B6" or "B7" or "B9" or "B12" or (B adj ("1" or "2" or "3" or "5" or "6" or "7" or "9" or "12")) or "methylmalonic acid" or "methyl malonate" or "methyl malonic acid" or methylmalonate or retinol or carotene or "Aquasol A" or phytomenadione or menaquinone or menadione or calcium or chloride or magnesium or phosphorus or romag or potassium or kalium or sodium or chromium or copper or fluoride or iodine or iron or "Fe" or "Fe+++" or ferro or ferrum or manganese or molybdenum or selenium or zinc).ti,ab.

17. 15 and 16

© Mayo Clinic Libraries, Mayo Foundation for Medical Education and Research

18. ((("complete blood" or "red blood cell" or erythrocyte or RBC or iron or reticulocyte) adj1 (count or indices)) or "CBC" or "hemogram" or (("coronary risk" or lipid or cholesterol or prothrombin or prothrombine or protrombin) adj1 (panel or test)) or Thrombotest or "Fe Tests").ti,ab.

19. (undernutrition* or malnutrition* or avitaminosis or ((nutrition* or mineral* or metabolic or nutrient* or micronutrient* or micro-nutrient*) adj2 (deficien* or diseases or disorder*))).ti,ab.

20. (beriberi or pellagra or scurvy or rickets or osteomalacia or hypocalcem* or hypo-calcem* or hypercalcem* or hyper-calcem* or hypocalcaem* or hypo-calcaem* or hypercalcaem* or hyper-calcaem* or hypocalciur* or hypo-calciur* or hypercalciur* or hyper-calciur* or hypomagnesem* or hypo-magnesem* or hypermagnesem* or hyper-magnesem* or hypomagnesaem* or hypo-magnesaem* or hypermagnesaem* or hyper-magnesaem* or hypoalbuminem* or hypo-albuminem* or hyperalbuminem* or hyper-albuminem* or hypoalbuminaem* or hypo-albuminaem* or hyperalbuminaem* or hyper-albuminaem* or hypokalem* or hypo-kalem* or hyperkalem* or hyper-kalem* or hypokalaem* or hypo-kalaem* or hyperkalaem* or hyper-kalaem* or hypohomocysteinem* or hypo-homocysteinem* or hyperhomocysteinem* or hyper-homocysteinem*).ti,ab.

21. 17 or 18 or 19 or 20

22. 14 and 21

23 limit 22 to yr="2020 -Current"

***4.* <u>Ovid EBM Reviews - Cochrane Database of Systematic Reviews</u>** 2005 to June 03, 2020 – searched June 4, 2020.

1. ((peripheral adj2 (nerve* or nervous) adj2 (disease* or disorder*)) or "peripheral neuropath*").ti,ab.

2. (PNS adj2 (disease* or disorder*)).ti,ab.

3. 1 or 2

4. ("paclitaxel" or "abi 007" or "abi007" or "abraxane" or "albumin bound paclitaxel" or "anzatax" or "apealea" or "asotax" or "biotax" or "bms 181339" or "bms181339" or "bmy 45622" or "bmy45622" or "bristaxol" or "britaxol" or "coroxane" or "dts 301" or "dts301" or "endotag-1" or "formoxol" or "genexol" or "genexol pm" or "hunxol" or "ifaxol" or "infinnium" or "intaxel" or "mbt 0206" or "mbt0206" or "medixel" or "mitotax" or "nab paclitaxel" or "nanoparticle albumin bound paclitaxel" or "nsc 125973" or "nsc 673089" or "nsc125973" or "nsc673089" or "oas pac 100" or "oaspac100" or "oncogel" or "onxol" or "pacitaxel" or "paclitaxel nab" or "pacxel" or "padexol" or "parexel" or "paxceed" or "paxene" or "paxus" or "praxel" or "taxocris" or "taxol" or "taycovit" or "yewtaxan").ti,ab.

5. ("docetaxel" or "daxotel" or "dexotel" or "docefrez" or "docetaxel accord" or "lit 976" or "lit976" or "n debenzoyl n tert butoxycarbonyl 10 deacetyltaxol" or "n tert butoxycarbonyl 10 deacetyl n debenzoyltaxol" or "nsc 628503" or "nsc628503" or "oncodocel" or "rp 56976" or "rp56976" or "taxespira" or "taxoter" or "taxotere" or "texot").ti,ab.

© Mayo Clinic Libraries, Mayo Foundation for Medical Education and Research

6. ("oxaliplatin" or "axiplatin" or "crisapla" or "croloxat" or "dacotin" or "dacplat" or "ebeoxal" or "elatofen" or "eloxatin" or "eloxatine" or "elplat" or "geneplatin" or "heloxatin" or "lipoxal" or "mbp 426" or "mbp426" or "medoxa" or "oplat" or "oxalatoplatinum" or "oxalatplatin" or "oxali" or "oxalip" or "oxaliplan" or "oxaliplatina" or "oxaliprol" or "oxaliquid" or "oxalisan" or "oxalisin" or "oxalizor" or "oxaltic" or "oxaltina" or "oxaplamyl" or "oxaviatin" or "platox" or "plaxitin" or "rectoxal" or "riboxatin" or "rp 54780" or "rp54780" or "sinoxal" or "sr 96669" or "sr96669" or "transplastin" or "velminox" or "xaliplat" or "xoplan").ti,ab.

7. ("cisplatin" or "abiplatin" or "biocisplatinum" or "biocysplatinum" or "blastolem" or "briplatin" or "cddp ti" or "cis ddp" or "cis diamine dichloroplatinum" or "cis diaminechloroplatinum" or "cis diaminedichloroplatinum" or "cis diammine dichloroplatinum" or "cis diamminedichloroplatinum" or "cis dichloridiammineplatinum" or "cis dichloroadiamine platinum" or "cis dichlorodiamine platinum" or "cis dichlorodiammineplatinum" or "cis platinous diamino dichloride" or "cis platinum" or "cis platinum diamine dichloride" or "cis platinum diaminedichloride" or "cis platinum diamino dichloride" or "cis platinum diaminochloride" or "cis platinum diaminodichloride" or "cis platinum diammine dichloride" or "cis platinum diamminedichloride" or "cisplatin liposomal" or "cisplatin therapeutic implant" or "cisplatin-ebewe" or "cisplatine" or "cisplatino" or "cisplatinum" or "cisplatyl" or "citoplatino" or "cytoplatin" or "cytosplat" or "diamine dichloroplatinum" or "diaminodichloroplatinum" or "diamminedichloroplatinum" or "dichlorodiamine platinum" or "dichlorodiammineplatinum" or "docistin" or "elvecis" or "kemoplat" or "lederplatin" or "lipoplatin" or "liposomal cisplatin" or "mpi 5010" or "mpi5010" or "neoplatin" or "niyaplat" or "nk 801" or "noveldexis" or "nsc 119875" or "platamine" or "platamine rtu" or "platiblastin" or "platidiam" or "platimine" or "platinex" or "platinil" or "platinol" or "platinol aq" or "platinoxan" or "platinum diamine dichloride" or "platinum diaminedichloride" or "platinum diaminodichloride" or "platinum diamminedichloride" or "platiran" or "platistil" or "platistin" or "platosin" or "randa" or "romcis" or "sicatem" or "spi 077" or "tecnoplatin").ti,ab.

8. ("bortezomib" or "ldp 341" or "ldp341" or "mg 341" or "mg341" or "mln 341" or "mln341" or "ps 341" or "ps341" or "velcade" or "vincristine" or "l 37231" or "l37231" or "vin cristine" or "vincristin" or "vincrisul" or "vinblastine" or "le 29060" or "le29060" or "leukoblastin" or "rozevin" or "vin blastine" or "vinblastin" or "vincaleucoblastin" or "vincaleucoblastine" or "vincaleukoblastine" or "vincoblastin" or "vincoblastine" or "vincoleucoblastine" or "vincoleukoblastin" or "vinleucoblastine").ti,ab.

9. ("thalidomide" or "beta thalidomide" or "contergan" or "distaval" or "isomin" or "k 17" or "kedavon" or "kevadon" or "neurosedin" or "neurosedyne" or "nsc 66847" or "sedalis" or "shin naito" or "softenon" or "synovir" or "talimol" or "talizer" or "telagan" or "telargan" or "thado" or "thalidomid" or "thalidomide celgene" or "thalimodide" or "thalix" or "thalomid").ti,ab.

10. (antineoplastic or chemotherap* or chemo-therap* or ((anticancer* or anti-cancer* or anticarcino* or anti-carcino* or antitumor or anti-tumor or antitumour* or anti-tumour* or carcinostatic) adj2 (therap* or agent or agents or treatment* or management*)) or carcinochemo* or "tumor inhibitor" or "tumour inhibitor").ti,ab.

11. 4 or 5 or 6 or 7 or 8 or 9 or 10

12. 3 and 11

© Mayo Clinic Libraries, Mayo Foundation for Medical Education and Research

13. ((chemo* or antineoplas* or anti-neoplas* or anticancer* or anti-cancer* or anticarcino* or anti-carcino* or antitumor or anti-tumor or antitumour or anti-tumour or carcinochemo* or carcinostatic or "tumor inhibitor" or "tumour inhibitor") adj3 (neuropath* or neuralgia or neurotox* or mononeuropath* or mono-neuropath* or polyneuropath* or poly-neuropath*)).ti,ab.

14. 12 or 13

15. ((laboratory adj2 (marker or markers or values or test* or technique* or service or services or diagnos* or examin* or evaluat*)) or ("lab test" or "lab tests" or "lab result" or "lab results" or "lab value" or "lab values" or deficien*)).ti,ab.

16. (hemoglobin or hemoglobins or haemoglobin or hemoglobine or haemoglobine or haemoglobins or hemoglobulin or hematocrit or ferrohaemoglobin or ferrohemoglobin or erythrocytes or eryhem or albumin or albumen or prealbumin or pre-albumin or total-protein or leptin or globulin or globulins or polyglobulin or poly-globulin or choline or bursine or fagine or vidine or thiamin or thiamine or anemi* or anaemi* or aneurin or riboflavin or riboflavine or "flavin mononucleotide" or "flavin-adenine dinucleotide" or niacin or niacinamide or enduramide or nicobion or nicotinamide or nicotinsaureamid or papulex or "pantothenic acid" or "calcium pantothenate" or dexol or "zinc pantothenate" or "pyridoxal phosphate" or "pyridoxal 5-phosphate" or "pyridoxal-P" or biotin or folate or "folic acid" or folacin or folvite or folvit or "pteroylglutamic acid" or cobalamin or cobalamins or cyanocobalamin or eritron or "B1" or "B2" or "B3" or "B5" or "B6" or "B7" or "B9" or "B12" or (B adj ("1" or "2" or "3" or "5" or "6" or "7" or "9" or "12")) or "methylmalonic acid" or "methyl malonate" or "methyl malonic acid" or methylmalonate or retinol or carotene or "Aquasol A" or phytomenadione or menaquinone or menadione or calcium or chloride or magnesium or phosphorus or romag or potassium or kalium or sodium or chromium or copper or fluoride or iodine or iron or "Fe" or "Fe+++" or ferro or ferrum or manganese or molybdenum or selenium or zinc).ti,ab.

17. 15 and 16

18. ((("complete blood" or "red blood cell" or erythrocyte or RBC or iron or reticulocyte) adj1 (count or indices)) or "CBC" or "hemogram" or (("coronary risk" or lipid or cholesterol or prothrombin or prothrombine or protrombin) adj1 (panel or test)) or Thrombotest or "Fe Tests").ti,ab.

19. (undernutrition* or malnutrition* or avitaminosis or ((nutrition* or mineral* or metabolic or nutrient* or micronutrient* or micro-nutrient*) adj2 (deficien* or diseases or disorder*))).ti,ab.

20. (beriberi or pellagra or scurvy or rickets or osteomalacia or hypocalcem* or hypo-calcem* or hypercalcem* or hyper-calcem* or hypocalcaem* or hypo-calcaem* or hypercalcaem* or hyper-calcaem* or hypocalciur* or hypo-calciur* or hypercalciur* or hyper-calciur* or hypomagnesem* or hypo-magnesem* or hypermagnesem* or hyper-magnesem* or hypomagnesaem* or hypo-magnesaem* or hypermagnesaem* or hyper-magnesaem* or hypoalbuminem* or hypo-albuminem* or hyperalbuminem* or hyper-albuminem* or hypoalbuminaem* or hypo-albuminaem* or hyperalbuminaem* or hyper-albuminaem* or hypokalem* or hypo-kalem* or hyperkalem* or hyper-kalem* or hypokalaem* or hypo-kalaem* or hyperkalaem* or hyper-kalaem* or hypohomocysteinem* or hypo-homocysteinem* or hyperhomocysteinem* or hyper-homocysteinem*).ti,ab.

21. 17 or 18 or 19 or 20

© Mayo Clinic Libraries, Mayo Foundation for Medical Education and Research

22. 14 and 21

Update: Ovid EBM Reviews - **Cochrane Database of Systematic Reviews** 2005 to December 02, 2021 – date range searched: January 01, 2020 – current (searched on December 08, 2021).

1 ((peripheral adj2 (nerve* or nervous) adj2 (disease* or disorder*)) or "peripheral neuropath*").ti,ab.

2 (PNS adj2 (disease* or disorder*)).ti,ab.

3 1 or 2

Filter By Specific Date Year Range, From: 2020, To: 2030

5. Scopus Date range: 1823 to June 04, 2020 – searched June 04, 2020.

((((((((TITLE-ABS-KEY ((peripheral W/2 (nerve* OR nervous) W/2 (disease* OR disorder*)) OR "peripheral neuropath*")) OR (TITLE-ABS-KEY (pns W/2 (disease* OR disorder*))))) AND ((TITLE-ABS-KEY (antineoplastic OR chemotherap* OR chemo-therap* OR ((anticancer* OR anti-cancer* OR anticarcino* OR anti-carcino* OR antitumor OR anti-tumor OR antitumour* OR anti-tumour* OR carcinostatic) W/2 (therap* OR agent OR agents OR treatment* OR management*)) OR carcinochemo* OR "tumor inhibitor" OR "tumour inhibitor")) OR (TITLE-ABS-KEY ("paclitaxel" OR "abi 007" OR "abi007" OR "abraxane" OR "albumin bound paclitaxel" OR "anzatax" OR "apealea" OR "asotax" OR "biotax" OR "bms 181339" OR "bms181339" OR "bmy 45622" OR "bmy45622" OR "bristaxol" OR "britaxol" OR "coroxane" OR "dts 301" OR "dts301" OR "endotag-1" OR "formoxol" OR "genexol" OR "genexol pm" OR "hunxol" OR "ifaxol" OR "infinnium" OR "intaxel" OR "mbt 0206" OR "mbt0206" OR "medixel" OR "mitotax" OR "nab paclitaxel" OR "nanoparticle albumin bound paclitaxel" OR "nsc 125973" OR "nsc 673089" OR "nsc125973" OR "nsc673089" OR "oas pac 100" OR "oaspac100" OR "oncogel" OR "onxol" OR "pacitaxel" OR "paclitaxel nab" OR "pacxel" OR "padexol" OR "parexel" OR "paxceed" OR "paxene" OR "paxus" OR "praxel" OR "taxocris" OR "taxol" OR "taycovit" OR "yewtaxan")) OR (TITLE-ABS-KEY ("docetaxel" OR "daxotel" OR "dexotel" OR "docefrez" OR "docetaxel accord" OR "lit 976" OR "lit976" OR "n debenzoyl n tert butoxycarbonyl 10 deacetyltaxol" OR "n tert butoxycarbonyl 10 deacetyl n debenzoyltaxol" OR "nsc 628503" OR "nsc628503" OR "oncodocel" OR "rp 56976" OR "rp56976" OR "taxespira" OR "taxoter" OR "taxotere" OR "texot")) OR (TITLE-ABS-KEY ("oxaliplatin" OR "axiplatin" OR "crisapla" OR "croloxat" OR "dacotin" OR "dacplat" OR "ebeoxal" OR "elatofen" OR "eloxatin" OR "eloxatine" OR "elplat" OR "geneplatin" OR "heloxatin" OR "lipoxal" OR "mbp 426" OR "mbp426" OR "medoxa" OR "oplat" OR "oxalatoplatinum" OR "oxalatplatin" OR "oxali" OR "oxalip" OR "oxaliplan" OR "oxaliplatina" OR "oxaliprol" OR "oxaliquid" OR "oxalisan" OR "oxalisin" OR "oxalizor" OR "oxaltic" OR "oxaltina" OR "oxaplamyl" OR "oxaviatin" OR "platox" OR "plaxitin" OR "rectoxal" OR "riboxatin" OR "rp 54780" OR "rp54780" OR "sinoxal" OR "sr 96669" OR "sr96669" OR "transplastin" OR "velminox" OR "xaliplat" OR "xoplan")) OR (TITLE-ABS-KEY ("cisplatin" OR "abiplatin" OR "biocisplatinum" OR

© Mayo Clinic Libraries, Mayo Foundation for Medical Education and Research

"biocysplatinum" OR "blastolem" OR "briplatin" OR "cddp ti" OR "cis ddp" OR "cis diamine dichloroplatinum" OR "cis diaminechloroplatinum" OR "cis diaminedichloroplatinum" OR "cis diammine dichloroplatinum" OR "cis diamminedichloroplatinum" OR "cis dichloridiammineplatinum" OR "cis dichloroadiamine platinum" OR "cis dichlorodiamine platinum" OR "cis dichlorodiammineplatinum" OR "cis platinous diamino dichloride" OR "cis platinum" OR "cis platinum diamine dichloride" OR "cis platinum diaminedichloride" OR "cis platinum diamino dichloride" OR "cis platinum diaminochloride" OR "cis platinum diaminodichloride" OR "cis platinum diammine dichloride" OR "cis platinum diamminedichloride" OR "cisplatin liposomal" OR "cisplatin therapeutic implant" OR "cisplatin-ebewe" OR "cisplatine" OR "cisplatino" OR "cisplatinum" OR "cisplatyl" OR "citoplatino" OR "cytoplatin" OR "cytosplat" OR "diamine dichloroplatinum" OR "diaminodichloroplatinum" OR "diamminedichloroplatinum" OR "dichlorodiamine platinum" OR "dichlorodiammineplatinum" OR "docistin" OR "elvecis" OR "kemoplat" OR "lederplatin" OR "lipoplatin" OR "liposomal cisplatin" OR "mpi 5010" OR "mpi5010" OR "neoplatin" OR "niyaplat" OR "nk 801" OR "noveldexis" OR "nsc 119875" OR "platamine" OR "platamine rtu" OR "platiblastin" OR "platidiam" OR "platimine" OR "platinex" OR "platinil" OR "platinol" OR "platinol aq" OR "platinoxan" OR "platinum diamine dichloride" OR "platinum diaminedichloride" OR "platinum diaminodichloride" OR "platinum diamminedichloride" OR "platiran" OR "platistil" OR "platistin" OR "platosin" OR "randa" OR "romcis" OR "sicatem" OR "spi 077" OR "tecnoplatin")) OR (TITLE-ABS-KEY ("bortezomib" OR "ldp 341" OR "ldp341" OR "mg 341" OR "mg341" OR "mln 341" OR "mln341" OR "ps 341" OR "ps341" OR "velcade" OR "vincristine" OR "l 37231" OR "l37231" OR "vin cristine" OR "vincristin" OR "vincrisul" OR "vinblastine" OR "le 29060" OR "le29060" OR "leukoblastin" OR "rozevin" OR "vin blastine" OR "vinblastin" OR "vincaleucoblastin" OR "vincaleucoblastine" OR "vincaleukoblastine" OR "vincoblastin" OR "vincoblastine" OR "vincoleucoblastine" OR "vincoleukoblastin" OR "vinleucoblastine")) OR (TITLE-ABS-KEY ("thalidomide" OR "beta thalidomide" OR "contergan" OR "distaval" OR "isomin" OR "k 17" OR "kedavon" OR "kevadon" OR "neurosedin" OR "neurosedyne" OR "nsc 66847" OR "sedalis" OR "shin naito" OR "softenon" OR "synovir" OR "talimol" OR "talizer" OR "telagan" OR "telargan" OR "thado" OR "thalidomid" OR "thalidomide celgene" OR "thalimodide" OR "thalix" OR "thalomid")))) OR (TITLE-ABS-KEY ((chemo* OR antineoplas* OR anti-neoplas* OR anticancer* OR anti-cancer* OR anticarcino* OR anti-carcino* OR antitumor OR anti-tumor OR antitumour OR anti-tumour OR carcinochemo* OR carcinostatic OR "tumor inhibitor" OR "tumour inhibitor") W/3 (neuropath* OR neuralgia OR neurotox* OR mononeuropath* OR mono-neuropath* OR polyneuropath* OR poly-neuropath*)))) AND (((TITLE-ABS-KEY ((laboratory W/2 (marker OR markers OR values OR test* OR technique* OR service OR services OR diagnos* OR examin* OR evaluat*)) OR ("lab test" OR "lab tests" OR "lab result" OR "lab results" OR "lab value" OR "lab values" OR deficien*))) AND (TITLE-ABS-KEY (hemoglobin OR hemoglobins OR haemoglobin OR hemoglobine OR haemoglobine OR haemoglobins OR hemoglobulin OR hematocrit OR ferrohaemoglobin OR ferrohemoglobin OR erythrocytes OR eryhem OR albumin OR albumen OR prealbumin OR pre-albumin OR total-protein OR leptin OR globulin OR globulins OR polyglobulin OR poly-globulin OR choline OR bursine OR fagine OR vidine OR thiamin OR thiamine OR anemi* OR anaemi* OR aneurin OR riboflavin OR riboflavine OR "flavin mononucleotide" OR "flavin-adenine dinucleotide" OR niacin OR niacinamide OR enduramide OR nicobion OR nicotinamide OR nicotinsaureamid OR papulex OR "pantothenic acid" OR "calcium pantothenate" OR dexol OR "zinc pantothenate" OR "pyridoxal phosphate" OR "pyridoxal 5-phosphate" OR "pyridoxal-P" OR biotin OR folate OR "folic acid" OR folacin OR folvite OR folvit OR "pteroylglutamic acid" OR cobalamin OR cobalamins OR

© Mayo Clinic Libraries, Mayo Foundation for Medical Education and Research

cyanocobalamin OR eritron OR "B1" OR "B2" OR "B3" OR "B5" OR "B6" OR "B7" OR "B9" OR "B12" OR (b W/1 ("1" OR "2" OR "3" OR "5" OR "6" OR "7" OR "9" OR "12")) OR "methylmalonic acid" OR "methyl malonate" OR "methyl malonic acid" OR methylmalonate OR retinol OR carotene OR "Aquasol A" OR phytomenadione OR menaquinone OR menadione OR calcium OR chloride OR magnesium OR phosphorus OR romag OR potassium OR kalium OR sodium OR chromium OR copper OR fluoride OR iodine OR iron OR "Fe" OR "Fe+++" OR ferro OR ferrum OR manganese OR molybdenum OR selenium OR zinc))) OR (TITLE-ABS-KEY ((("complete blood" OR "red blood cell" OR erythrocyte OR rbc OR iron OR reticulocyte) W/1 (count OR indices)) OR "CBC" OR "hemogram" OR (("coronary risk" OR lipid OR cholesterol OR prothrombin OR prothrombine OR protrombin) W/1 (panel OR test)) OR thrombotest OR "Fe Tests")) OR (TITLE-ABS-KEY (undernutrition* OR malnutrition* OR avitaminosis OR ((nutrition* OR mineral* OR metabolic OR nutrient* OR micronutrient* OR micro-nutrient*) W/2 (deficien* OR diseases OR disorder*)))) OR (TITLE-ABS-KEY (beriberi OR pellagra OR scurvy OR rickets OR osteomalacia OR hypocalcem* OR hypo-calcem* OR hypercalcem* OR hyper-calcem* OR hypocalcaem* OR hypo-calcaem* OR hypercalcaem* OR hyper-calcaem* OR hypocalciur* OR hypo-calciur* OR hypercalciur* OR hyper-calciur* OR hypomagnesem* OR hypo-magnesem* OR hypermagnesem* OR hyper-magnesem* OR hypomagnesaem* OR hypo-magnesaem* OR hypermagnesaem* OR hyper-magnesaem* OR hypoalbuminem* OR hypo-albuminem* OR hyperalbuminem* OR hyper-albuminem* OR hypoalbuminaem* OR hypo-albuminaem* OR hyperalbuminaem* OR hyper-albuminaem* OR hypokalem* OR hypo-kalem* OR hyperkalem* OR hyper-kalem* OR hypokalaem* OR hypo-kalaem* OR hyperkalaem* OR hyper-kalaem* OR hypohomocysteinem* OR hypo-homocysteinem* OR hyperhomocysteinem* OR hyper-homocysteinem*)))) AND NOT ((PMID (0*) OR PMID (1*) OR PMID (2*) OR PMID (3*) OR PMID (4*) OR PMID (5*) OR PMID (6*) OR PMID (7*) OR PMID (8*) OR PMID (9*)))) AND NOT (TITLE-ABS (case W/1 (report* OR series)))) AND NOT (TITLE-ABS (infan* OR newborn* OR new-born* OR perinat* OR neonat* OR baby OR baby* OR babies OR toddler* OR minors OR minors* OR boy OR boys OR boyfriend OR boyhood OR girl* OR kid OR kids OR child OR child* OR children* OR schoolchild* OR schoolchild OR adolescen* OR juvenil* OR youth* OR teen* OR under*age* OR pubescen* OR pediatric* OR paediatric* OR peadiatric* OR prematur* OR preterm*OR school child OR "school child*" OR school OR school*))) or ((((((((TITLE-ABS-KEY ((peripheral W/2 (nerve* OR nervous) W/2 (disease* OR disorder*)) OR "peripheral neuropath*")) OR (TITLE-ABS-KEY (pns W/2 (disease* OR disorder*))))) AND ((TITLE-ABS-KEY (antineoplastic OR chemotherap* OR chemo-therap* OR ((anticancer* OR anti-cancer* OR anticarcino* OR anti-carcino* OR antitumor OR anti-tumor OR antitumour* OR anti-tumour* OR carcinostatic) W/2 (therap* OR agent OR agents OR treatment* OR management*)) OR carcinochemo* OR "tumor inhibitor" OR "tumour inhibitor")) OR (TITLE-ABS-KEY ("paclitaxel" OR "abi 007" OR "abi007" OR "abraxane" OR "albumin bound paclitaxel" OR "anzatax" OR "apealea" OR "asotax" OR "biotax" OR "bms 181339" OR "bms181339" OR "bmy 45622" OR "bmy45622" OR "bristaxol" OR "britaxol" OR "coroxane" OR "dts 301" OR "dts301" OR "endotag-1" OR "formoxol" OR "genexol" OR "genexol pm" OR "hunxol" OR "ifaxol" OR "infinnium" OR "intaxel" OR "mbt 0206" OR "mbt0206" OR "medixel" OR "mitotax" OR "nab paclitaxel" OR "nanoparticle albumin bound paclitaxel" OR "nsc 125973" OR "nsc 673089" OR "nsc125973" OR "nsc673089" OR "oas pac 100" OR "oaspac100" OR "oncogel" OR "onxol" OR "pacitaxel" OR "paclitaxel nab" OR "pacxel" OR "padexol" OR "parexel" OR "paxceed" OR "paxene" OR "paxus" OR "praxel" OR "taxocris" OR "taxol" OR "taycovit" OR "yewtaxan")) OR (TITLE-ABS-KEY ("docetaxel" OR "daxotel" OR "dexotel" OR

© Mayo Clinic Libraries, Mayo Foundation for Medical Education and Research

"docefrez" OR "docetaxel accord" OR "lit 976" OR "lit976" OR "n debenzoyl n tert butoxycarbonyl 10 deacetyltaxol" OR "n tert butoxycarbonyl 10 deacetyl n debenzoyltaxol" OR "nsc 628503" OR "nsc628503" OR "oncodocel" OR "rp 56976" OR "rp56976" OR "taxespira" OR "taxoter" OR "taxotere" OR "texot")) OR (TITLE-ABS-KEY ("oxaliplatin" OR "axiplatin" OR "crisapla" OR "croloxat" OR "dacotin" OR "dacplat" OR "ebeoxal" OR "elatofen" OR "eloxatin" OR "eloxatine" OR "elplat" OR "geneplatin" OR "heloxatin" OR "lipoxal" OR "mbp 426" OR "mbp426" OR "medoxa" OR "oplat" OR "oxalatoplatinum" OR "oxalatplatin" OR "oxali" OR "oxalip" OR "oxaliplan" OR "oxaliplatina" OR "oxaliprol" OR "oxaliquid" OR "oxalisan" OR "oxalisin" OR "oxalizor" OR "oxaltic" OR "oxaltina" OR "oxaplamyl" OR "oxaviatin" OR "platox" OR "plaxitin" OR "rectoxal" OR "riboxatin" OR "rp 54780" OR "rp54780" OR "sinoxal" OR "sr 96669" OR "sr96669" OR "transplastin" OR "velminox" OR "xaliplat" OR "xoplan")) OR (TITLE-ABS-KEY ("cisplatin" OR "abiplatin" OR "biocisplatinum" OR "biocysplatinum" OR "blastolem" OR "briplatin" OR "cddp ti" OR "cis ddp" OR "cis diamine dichloroplatinum" OR "cis diaminechloroplatinum" OR "cis diaminedichloroplatinum" OR "cis diammine dichloroplatinum" OR "cis diamminedichloroplatinum" OR "cis dichloridiammineplatinum" OR "cis dichloroadiamine platinum" OR "cis dichlorodiamine platinum" OR "cis dichlorodiammineplatinum" OR "cis platinous diamino dichloride" OR "cis platinum" OR "cis platinum diamine dichloride" OR "cis platinum diaminedichloride" OR "cis platinum diamino dichloride" OR "cis platinum diaminochloride" OR "cis platinum diaminodichloride" OR "cis platinum diammine dichloride" OR "cis platinum diamminedichloride" OR "cisplatin liposomal" OR "cisplatin therapeutic implant" OR "cisplatin-ebewe" OR "cisplatine" OR "cisplatino" OR "cisplatinum" OR "cisplatyl" OR "citoplatino" OR "cytoplatin" OR "cytosplat" OR "diamine dichloroplatinum" OR "diaminodichloroplatinum" OR "diamminedichloroplatinum" OR "dichlorodiamine platinum" OR "dichlorodiammineplatinum" OR "docistin" OR "elvecis" OR "kemoplat" OR "lederplatin" OR "lipoplatin" OR "liposomal cisplatin" OR "mpi 5010" OR "mpi5010" OR "neoplatin" OR "niyaplat" OR "nk 801" OR "noveldexis" OR "nsc 119875" OR "platamine" OR "platamine rtu" OR "platiblastin" OR "platidiam" OR "platimine" OR "platinex" OR "platinil" OR "platinol" OR "platinol aq" OR "platinoxan" OR "platinum diamine dichloride" OR "platinum diaminedichloride" OR "platinum diaminodichloride" OR "platinum diamminedichloride" OR "platiran" OR "platistil" OR "platistin" OR "platosin" OR "randa" OR "romcis" OR "sicatem" OR "spi 077" OR "tecnoplatin")) OR (TITLE-ABS-KEY ("bortezomib" OR "ldp 341" OR "ldp341" OR "mg 341" OR "mg341" OR "mln 341" OR "mln341" OR "ps 341" OR "ps341" OR "velcade" OR "vincristine" OR "l 37231" OR "l37231" OR "vin cristine" OR "vincristin" OR "vincrisul" OR "vinblastine" OR "le 29060" OR "le29060" OR "leukoblastin" OR "rozevin" OR "vin blastine" OR "vinblastin" OR "vincaleucoblastin" OR "vincaleucoblastine" OR "vincaleukoblastine" OR "vincoblastin" OR "vincoblastine" OR "vincoleucoblastine" OR "vincoleukoblastin" OR "vinleucoblastine")) OR (TITLE-ABS-KEY ("thalidomide" OR "beta thalidomide" OR "contergan" OR "distaval" OR "isomin" OR "k 17" OR "kedavon" OR "kevadon" OR "neurosedin" OR "neurosedyne" OR "nsc 66847" OR "sedalis" OR "shin naito" OR "softenon" OR "synovir" OR "talimol" OR "talizer" OR "telagan" OR "telargan" OR "thado" OR "thalidomid" OR "thalidomide celgene" OR "thalimodide" OR "thalix" OR "thalomid")))) OR (TITLE-ABS-KEY ((chemo* OR antineoplas* OR anti-neoplas* OR anticancer* OR anti-cancer* OR anticarcino* OR anti-carcino* OR antitumor OR anti-tumor OR antitumour OR anti-tumour OR carcinochemo* OR carcinostatic OR "tumor inhibitor" OR "tumour inhibitor") W/3 (neuropath* OR neuralgia OR neurotox* OR mononeuropath* OR mono-neuropath* OR polyneuropath* OR poly-neuropath*)))) AND (((TITLE-ABS-KEY ((laboratory W/2 (marker OR markers OR values OR test* OR technique* OR service OR services OR diagnos* OR examin* OR evaluat*)) OR (

© Mayo Clinic Libraries, Mayo Foundation for Medical Education and Research

"lab test" OR "lab tests" OR "lab result" OR "lab results" OR "lab value" OR "lab values" OR deficien*))) AND (TITLE-ABS-KEY (hemoglobin OR hemoglobins OR haemoglobin OR hemoglobine OR haemoglobine OR haemoglobins OR hemoglobulin OR hematocrit OR ferrohaemoglobin OR ferrohemoglobin OR erythrocytes OR eryhem OR albumin OR albumen OR prealbumin OR pre-albumin OR total-protein OR leptin OR globulin OR globulins OR polyglobulin OR poly-globulin OR choline OR bursine OR fagine OR vidine OR thiamin OR thiamine OR anemi* OR anaemi* OR aneurin OR riboflavin OR riboflavine OR "flavin mononucleotide" OR "flavin-adenine dinucleotide" OR niacin OR niacinamide OR enduramide OR nicobion OR nicotinamide OR nicotinsaureamid OR papulex OR "pantothenic acid" OR "calcium pantothenate" OR dexol OR "zinc pantothenate" OR "pyridoxal phosphate" OR "pyridoxal 5-phosphate" OR "pyridoxal-P" OR biotin OR folate OR "folic acid" OR folacin OR folvite OR folvit OR "pteroylglutamic acid" OR cobalamin OR cobalamins OR cyanocobalamin OR eritron OR "B1" OR "B2" OR "B3" OR "B5" OR "B6" OR "B7" OR "B9" OR "B12" OR (b W/1 ("1" OR "2" OR "3" OR "5" OR "6" OR "7" OR "9" OR "12")) OR "methylmalonic acid" OR "methyl malonate" OR "methyl malonic acid" OR methylmalonate OR retinol OR carotene OR "Aquasol A" OR phytomenadione OR menaquinone OR menadione OR calcium OR chloride OR magnesium OR phosphorus OR romag OR potassium OR kalium OR sodium OR chromium OR copper OR fluoride OR iodine OR iron OR "Fe" OR "Fe+++" OR ferro OR ferrum OR manganese OR molybdenum OR selenium OR zinc))) OR (TITLE-ABS-KEY ((("complete blood" OR "red blood cell" OR erythrocyte OR rbc OR iron OR reticulocyte) W/1 (count OR indices)) OR "CBC" OR "hemogram" OR (("coronary risk" OR lipid OR cholesterol OR prothrombin OR prothrombine OR protrombin) W/1 (panel OR test)) OR thrombotest OR "Fe Tests")) OR (TITLE-ABS-KEY (undernutrition* OR malnutrition* OR avitaminosis OR ((nutrition* OR mineral* OR metabolic OR nutrient* OR micronutrient* OR micro-nutrient*) W/2 (deficien* OR diseases OR disorder*)))) OR (TITLE-ABS-KEY (beriberi OR pellagra OR scurvy OR rickets OR osteomalacia OR hypocalcem* OR hypo-calcem* OR hypercalcem* OR hyper-calcem* OR hypocalcaem* OR hypo-calcaem* OR hypercalcaem* OR hyper-calcaem* OR hypocalciur* OR hypo-calciur* OR hypercalciur* OR hyper-calciur* OR hypomagnesem* OR hypo-magnesem* OR hypermagnesem* OR hyper-magnesem* OR hypomagnesaem* OR hypo-magnesaem* OR hypermagnesaem* OR hyper-magnesaem* OR hypoalbuminem* OR hypo-albuminem* OR hyperalbuminem* OR hyper-albuminem* OR hypoalbuminaem* OR hypo-albuminaem* OR hyperalbuminaem* OR hyper-albuminaem* OR hypokalem* OR hypo-kalem* OR hyperkalem* OR hyper-kalem* OR hypokalaem* OR hypo-kalaem* OR hyperkalaem* OR hyper-kalaem* OR hypohomocysteinem* OR hypo-homocysteinem* OR hyperhomocysteinem* OR hyper-homocysteinem*)))) AND NOT ((PMID (0*) OR PMID (1*) OR PMID (2*) OR PMID (3*) OR PMID (4*) OR PMID (5*) OR PMID (6*) OR PMID (7*) OR PMID (8*) OR PMID (9*)))) AND NOT (TITLE-ABS (case W/1 (report* OR series)))) AND (TITLE-ABS (adult or adults or aged or elderly)))

Update: **Scopus** Date range: 1823 to December 08, 2021 – searched December 08, 2021.

((((((((TITLE-ABS-KEY ((peripheral W/2 (nerve* OR nervous) W/2 (disease* OR disorder*)) OR "peripheral neuropath*")) OR (TITLE-ABS-KEY (pns W/2 (disease* OR disorder*)))) AND ((TITLE-ABS-KEY (antineoplastic OR chemotherap* OR chemo-therap* OR ((anticancer* OR anti-cancer* OR anticarcino* OR anti-carcino* OR antitumor OR anti-

© Mayo Clinic Libraries, Mayo Foundation for Medical Education and Research

tumor OR antitumour* OR anti-tumour* OR carcinostatic) W/2 (therap* OR agent OR agents OR treatment* OR management*)) OR carcinochemo* OR "tumor inhibitor" OR "tumour inhibitor")) OR (TITLE-ABS-KEY ("paclitaxel" OR "abi 007" OR "abi007" OR "abraxane" OR "albumin bound paclitaxel" OR "anzatax" OR "apealea" OR "asotax" OR "biotax" OR "bms 181339" OR "bms181339" OR "bmy 45622" OR "bmy45622" OR "bristaxol" OR "britaxol" OR "coroxane" OR "dts 301" OR "dts301" OR "endotag-1" OR "formoxol" OR "genexol" OR "genexol pm" OR "hunxol" OR "ifaxol" OR "infinnium" OR "intaxel" OR "mbt 0206" OR "mbt0206" OR "medixel" OR "mitotax" OR "nab paclitaxel" OR "nanoparticle albumin bound paclitaxel" OR "nsc 125973" OR "nsc 673089" OR "nsc125973" OR "nsc673089" OR "oas pac 100" OR "oaspac100" OR "oncogel" OR "onxol" OR "pacitaxel" OR "paclitaxel nab" OR "pacxel" OR "padexol" OR "parexel" OR "paxceed" OR "paxene" OR "paxus" OR "praxel" OR "taxocris" OR "taxol" OR "taycovit" OR "yewtaxan")) OR (TITLE-ABS-KEY ("docetaxel" OR "daxotel" OR "dexotel" OR "docefrez" OR "docetaxel accord" OR "lit 976" OR "lit976" OR "n debenzoyl n tert butoxycarbonyl 10 deacetyltaxol" OR "n tert butoxycarbonyl 10 deacetyl n debenzoyltaxol" OR "nsc 628503" OR "nsc628503" OR "oncodocel" OR "rp 56976" OR "rp56976" OR "taxespira" OR "taxoter" OR "taxotere" OR "texot")) OR (TITLE-ABS-KEY ("oxaliplatin" OR "axiplatin" OR "crisapla" OR "croloxat" OR "dacotin" OR "dacplat" OR "ebeoxal" OR "elatofen" OR "eloxatin" OR "eloxatine" OR "elplat" OR "geneplatin" OR "heloxatin" OR "lipoxal" OR "mbp 426" OR "mbp426" OR "medoxa" OR "oplat" OR "oxalatoplatinum" OR "oxalatplatin" OR "oxali" OR "oxalip" OR "oxaliplan" OR "oxaliplatina" OR "oxaliprol" OR "oxaliquid" OR "oxalisan" OR "oxalisin" OR "oxalizor" OR "oxaltic" OR "oxaltina" OR "oxaplamyl" OR "oxaviatin" OR "platox" OR "plaxitin" OR "rectoxal" OR "riboxatin" OR "rp 54780" OR "rp54780" OR "sinoxal" OR "sr 96669" OR "sr96669" OR "transplastin" OR "velminox" OR "xaliplat" OR "xoplan")) OR (TITLE-ABS-KEY ("cisplatin" OR "abiplatin" OR "biocisplatinum" OR "biocysplatinum" OR "blastolem" OR "briplatin" OR "cddp ti" OR "cis ddp" OR "cis diamine dichloroplatinum" OR "cis diaminechloroplatinum" OR "cis diaminedichloroplatinum" OR "cis diammine dichloroplatinum" OR "cis diamminedichloroplatinum" OR "cis dichloridiammineplatinum" OR "cis dichloroadiamine platinum" OR "cis dichlorodiamine platinum" OR "cis dichlorodiammineplatinum" OR "cis platinous diamino dichloride" OR "cis platinum" OR "cis platinum diamine dichloride" OR "cis platinum diaminedichloride" OR "cis platinum diamino dichloride" OR "cis platinum diaminochloride" OR "cis platinum diaminodichloride" OR "cis platinum diammine dichloride" OR "cis platinum diamminedichloride" OR "cisplatin liposomal" OR "cisplatin therapeutic implant" OR "cisplatin-ebewe" OR "cisplatine" OR "cisplatino" OR "cisplatinum" OR "cisplatyl" OR "citoplatino" OR "cytoplatin" OR "cytosplat" OR "diamine dichloroplatinum" OR "diaminodichloroplatinum" OR "diamminedichloroplatinum" OR "dichlorodiamine platinum" OR "dichlorodiammineplatinum" OR "docistin" OR "elvecis" OR "kemoplat" OR "lederplatin" OR "lipoplatin" OR "liposomal cisplatin" OR "mpi 5010" OR "mpi5010" OR "neoplatin" OR "niyaplat" OR "nk 801" OR "noveldexis" OR "nsc 119875" OR "platamine" OR "platamine rtu" OR "platiblastin" OR "platidiam" OR "platimine" OR "platinex" OR "platinil" OR "platinol" OR "platinol aq" OR "platinoxan" OR "platinum diamine dichloride" OR "platinum diaminedichloride" OR "platinum diaminodichloride" OR "platinum diamminedichloride" OR "platiran" OR "platistil" OR "platistin" OR "platosin" OR "randa" OR "romcis" OR "sicatem" OR "spi 077" OR "tecnoplatin")) OR (TITLE-ABS-KEY ("bortezomib" OR "ldp 341" OR "ldp341" OR "mg 341" OR "mg341" OR "mln 341" OR "mln341" OR "ps

© Mayo Clinic Libraries, Mayo Foundation for Medical Education and Research

341" OR "ps341" OR "velcade" OR "vincristine" OR "l 37231" OR "l37231" OR "vin cristine" OR "vincristin" OR "vincrisul" OR "vinblastine" OR "le 29060" OR "le29060" OR "leukoblastin" OR "rozevin" OR "vin blastine" OR "vinblastin" OR "vincaleucoblastin" OR "vincaleucoblastine" OR "vincaleukoblastine" OR "vincoblastin" OR "vincoblastine" OR "vincoleucoblastine" OR "vincoleukoblastin" OR "vinleucoblastine")) OR (TITLE-ABS-KEY ("thalidomide" OR "beta thalidomide" OR "contergan" OR "distaval" OR "isomin" OR "k 17" OR "kedavon" OR "kevadon" OR "neurosedin" OR "neurosedyne" OR "nsc 66847" OR "sedalis" OR "shin naito" OR "softenon" OR "synovir" OR "talimol" OR "talizer" OR "telagan" OR "telargan" OR "thado" OR "thalidomid" OR "thalidomide celgene" OR "thalimodide" OR "thalix" OR "thalomid")))) OR (TITLE-ABS-KEY ((chemo* OR antineoplas* OR anti-neoplas* OR anticancer* OR anti-cancer* OR anticarcino* OR anti-carcino* OR antitumor OR anti-tumor OR antitumour OR anti-tumour OR carcinochemo* OR carcinostatic OR "tumor inhibitor" OR "tumour inhibitor") W/3 (neuropath* OR neuralgia OR neurotox* OR mononeuropath* OR mono-neuropath* OR polyneuropath* OR poly-neuropath*)))) AND (((TITLE-ABS-KEY ((laboratory W/2 (marker OR markers OR values OR test* OR technique* OR service OR services OR diagnos* OR examin* OR evaluat*)) OR ("lab test" OR "lab tests" OR "lab result" OR "lab results" OR "lab value" OR "lab values" OR deficien*))) AND (TITLE-ABS-KEY (hemoglobin OR hemoglobins OR haemoglobin OR hemoglobine OR haemoglobine OR haemoglobins OR hemoglobulin OR hematocrit OR ferrohaemoglobin OR ferrohemoglobin OR erythrocytes OR eryhem OR albumin OR albumen OR prealbumin OR pre-albumin OR total-protein OR leptin OR globulin OR globulins OR polyglobulin OR poly-globulin OR choline OR bursine OR fagine OR vidine OR thiamin OR thiamine OR anemi* OR anaemi* OR aneurin OR riboflavin OR riboflavine OR "flavin mononucleotide" OR "flavin-adenine dinucleotide" OR niacin OR niacinamide OR enduramide OR nicobion OR nicotinamide OR nicotinsaureamid OR papulex OR "pantothenic acid" OR "calcium pantothenate" OR dexol OR "zinc pantothenate" OR "pyridoxal phosphate" OR "pyridoxal 5-phosphate" OR "pyridoxal-P" OR biotin OR folate OR "folic acid" OR folacin OR folvite OR folvit OR "pteroylglutamic acid" OR cobalamin OR cobalamins OR cyanocobalamin OR eritron OR "B1" OR "B2" OR "B3" OR "B5" OR "B6" OR "B7" OR "B9" OR "B12" OR (b W/1 ("1" OR "2" OR "3" OR "5" OR "6" OR "7" OR "9" OR "12")) OR "methylmalonic acid" OR "methyl malonate" OR "methyl malonic acid" OR methylmalonate OR retinol OR carotene OR "Aquasol A" OR phytomenadione OR menaquinone OR menadione OR calcium OR chloride OR magnesium OR phosphorus OR romag OR potassium OR kalium OR sodium OR chromium OR copper OR fluoride OR iodine OR iron OR "Fe" OR "Fe+++" OR ferro OR ferrum OR manganese OR molybdenum OR selenium OR zinc))) OR (TITLE-ABS-KEY ((("complete blood" OR "red blood cell" OR erythrocyte OR rbc OR iron OR reticulocyte) W/1 (count OR indices)) OR "CBC" OR "hemogram" OR (("coronary risk" OR lipid OR cholesterol OR prothrombin OR prothrombine OR protrombin) W/1 (panel OR test)) OR thrombotest OR "Fe Tests")) OR (TITLE-ABS-KEY (undernutrition* OR malnutrition* OR avitaminosis OR ((nutrition* OR mineral* OR metabolic OR nutrient* OR micronutrient* OR micro-nutrient*) W/2 (deficien* OR diseases OR disorder*)))) OR (TITLE-ABS-KEY (beriberi OR pellagra OR scurvy OR rickets OR osteomalacia OR hypocalcem* OR hypo-calcem* OR hypercalcem* OR hyper-calcem* OR hypocalcaem* OR hypo-calcaem* OR hypercalcaem* OR hyper-calcaem* OR hypocalciur* OR hypo-calciur* OR hypercalciur* OR hyper-calciur* OR hypomagnesem* OR hypo-magnesem* OR hypermagnesem* OR hyper-magnesem* OR hypomagnesaem* OR hypo-

© Mayo Clinic Libraries, Mayo Foundation for Medical Education and Research

magnesaem* OR hypermagnesaem* OR hyper-magnesaem* OR hypoalbuminem* OR hypo-albuminem* OR hyperalbuminem* OR hyper-albuminem* OR hypoalbuminaem* OR hypo-albuminaem* OR hyperalbuminaem* OR hyper-albuminaem* OR hypokalem* OR hypo-kalem* OR hyperkalem* OR hyper-kalem* OR hypokalaem* OR hypo-kalaem* OR hyperkalaem* OR hyper-kalaem* OR hypohomocysteinem* OR hypo-homocysteinem* OR hyperhomocysteinem* OR hyper-homocysteinem*)))) AND NOT ((PMID (0*) OR PMID (1*) OR PMID (2*) OR PMID (3*) OR PMID (4*) OR PMID (5*) OR PMID (6*) OR PMID (7*) OR PMID (8*) OR PMID (9*)))) AND NOT (TITLE-ABS (case W/1 (report* OR series)))) AND NOT (TITLE-ABS (infan* OR newborn* OR new-born* OR perinat* OR neonat* OR baby OR baby* OR babies OR toddler* OR minors OR minors* OR boy OR boys OR boyfriend OR boyhood OR girl* OR kid OR kids OR child OR child* OR children* OR schoolchild* OR schoolchild OR adolescen* OR juvenil* OR youth* OR teen* OR under*age* OR pubescen* OR pediatric* OR paediatric* OR peadiatric* OR prematur* OR preterm*or AND school AND child OR "school child*" OR school OR school*))) OR ((((((((TITLE-ABS-KEY ((peripheral W/2 (nerve* OR nervous) W/2 (disease* OR disorder*)) OR "peripheral neuropath*")) OR (TITLE-ABS-KEY (pns W/2 (disease* OR disorder*)))) AND ((TITLE-ABS-KEY (antineoplastic OR chemotherap* OR chemo-therap* OR ((anticancer* OR anti-cancer* OR anticarcino* OR anti-carcino* OR antitumor OR anti-tumor OR antitumour* OR anti-tumour* OR carcinostatic) W/2 (therap* OR agent OR agents OR treatment* OR management*)) OR carcinochemo* OR "tumor inhibitor" OR "tumour inhibitor")) OR (TITLE-ABS-KEY ("paclitaxel" OR "abi 007" OR "abi007" OR "abraxane" OR "albumin bound paclitaxel" OR "anzatax" OR "apealea" OR "asotax" OR "biotax" OR "bms 181339" OR "bms181339" OR "bmy 45622" OR "bmy45622" OR "bristaxol" OR "britaxol" OR "coroxane" OR "dts 301" OR "dts301" OR "endotag-1" OR "formoxol" OR "genexol" OR "genexol pm" OR "hunxol" OR "ifaxol" OR "infinnium" OR "intaxel" OR "mbt 0206" OR "mbt0206" OR "medixel" OR "mitotax" OR "nab paclitaxel" OR "nanoparticle albumin bound paclitaxel" OR "nsc 125973" OR "nsc 673089" OR "nsc125973" OR "nsc673089" OR "oas pac 100" OR "oaspac100" OR "oncogel" OR "onxol" OR "pacitaxel" OR "paclitaxel nab" OR "pacxel" OR "padexol" OR "parexel" OR "paxceed" OR "paxene" OR "paxus" OR "praxel" OR "taxocris" OR "taxol" OR "taycovit" OR "yewtaxan")) OR (TITLE-ABS-KEY ("docetaxel" OR "daxotel" OR "dexotel" OR "docefrez" OR "docetaxel accord" OR "lit 976" OR "lit976" OR "n debenzoyl n tert butoxycarbonyl 10 deacetyltaxol" OR "n tert butoxycarbonyl 10 deacetyl n debenzoyltaxol" OR "nsc 628503" OR "nsc628503" OR "oncodocel" OR "rp 56976" OR "rp56976" OR "taxespira" OR "taxoter" OR "taxotere" OR "texot")) OR (TITLE-ABS-KEY ("oxaliplatin" OR "axiplatin" OR "crisapla" OR "croloxat" OR "dacotin" OR "dacplat" OR "ebeoxal" OR "elatofen" OR "eloxatin" OR "eloxatine" OR "elplat" OR "geneplatin" OR "heloxatin" OR "lipoxal" OR "mbp 426" OR "mbp426" OR "medoxa" OR "oplat" OR "oxalatoplatinum" OR "oxalatplatin" OR "oxali" OR "oxalip" OR "oxaliplan" OR "oxaliplatina" OR "oxaliprol" OR "oxaliquid" OR "oxalisan" OR "oxalisin" OR "oxalizor" OR "oxaltic" OR "oxaltina" OR "oxaplamyl" OR "oxaviatin" OR "platox" OR "plaxitin" OR "rectoxal" OR "riboxatin" OR "rp 54780" OR "rp54780" OR "sinoxal" OR "sr 96669" OR "sr96669" OR "transplastin" OR "velminox" OR "xaliplat" OR "xoplan")) OR (TITLE-ABS-KEY ("cisplatin" OR "abiplatin" OR "biocisplatinum" OR "biocysplatinum" OR "blastolem" OR "briplatin" OR "cddp ti" OR "cis ddp" OR "cis diamine dichloroplatinum" OR "cis diaminechloroplatinum" OR "cis diaminedichloroplatinum" OR "cis diammine dichloroplatinum" OR "cis diamminedichloroplatinum"

© Mayo Clinic Libraries, Mayo Foundation for Medical Education and Research

OR "cis dichloridiammineplatinum" OR "cis dichloroadiamine platinum" OR "cis dichlorodiamine platinum" OR "cis dichlorodiammineplatinum" OR "cis platinous diamino dichloride" OR "cis platinum" OR "cis platinum diamine dichloride" OR "cis platinum diaminedichloride" OR "cis platinum diamino dichloride" OR "cis platinum diaminochloride" OR "cis platinum diaminodichloride" OR "cis platinum diammine dichloride" OR "cis platinum diamminedichloride" OR "cisplatin liposomal" OR "cisplatin therapeutic implant" OR "cisplatin-ebewe" OR "cisplatine" OR "cisplatino" OR "cisplatinum" OR "cisplatyl" OR "citoplatino" OR "cytoplatin" OR "cytosplat" OR "diamine dichloroplatinum" OR "diaminodichloroplatinum" OR "diamminedichloroplatinum" OR "dichlorodiamine platinum" OR "dichlorodiammineplatinum" OR "docistin" OR "elvecis" OR "kemoplat" OR "lederplatin" OR "lipoplatin" OR "liposomal cisplatin" OR "mpi 5010" OR "mpi5010" OR "neoplatin" OR "niyaplat" OR "nk 801" OR "noveldexis" OR "nsc 119875" OR "platamine" OR "platamine rtu" OR "platiblastin" OR "platidiam" OR "platimine" OR "platinex" OR "platinil" OR "platinol" OR "platinol aq" OR "platinoxan" OR "platinum diamine dichloride" OR "platinum diaminedichloride" OR "platinum diaminodichloride" OR "platinum diamminedichloride" OR "platiran" OR "platistil" OR "platistin" OR "platosin" OR "randa" OR "romcis" OR "sicatem" OR "spi 077" OR "tecnoplatin")) OR (TITLE-ABS-KEY ("bortezomib" OR "ldp 341" OR "ldp341" OR "mg 341" OR "mg341" OR "mln 341" OR "mln341" OR "ps 341" OR "ps341" OR "velcade" OR "vincristine" OR "l 37231" OR "l37231" OR "vin cristine" OR "vincristin" OR "vincrisul" OR "vinblastine" OR "le 29060" OR "le29060" OR "leukoblastin" OR "rozevin" OR "vin blastine" OR "vinblastin" OR "vincaleucoblastin" OR "vincaleucoblastine" OR "vincaleukoblastine" OR "vincoblastin" OR "vincoblastine" OR "vincoleucoblastine" OR "vincoleukoblastin" OR "vinleucoblastine")) OR (TITLE-ABS-KEY ("thalidomide" OR "beta thalidomide" OR "contergan" OR "distaval" OR "isomin" OR "k 17" OR "kedavon" OR "kevadon" OR "neurosedin" OR "neurosedyne" OR "nsc 66847" OR "sedalis" OR "shin naito" OR "softenon" OR "synovir" OR "talimol" OR "talizer" OR "telagan" OR "telargan" OR "thado" OR "thalidomid" OR "thalidomide celgene" OR "thalimodide" OR "thalix" OR "thalomid")))) OR (TITLE-ABS-KEY ((chemo* OR antineoplas* OR anti-neoplas* OR anticancer* OR anti-cancer* OR anticarcino* OR anti-carcino* OR antitumor OR anti-tumor OR antitumour OR anti-tumour OR carcinochemo* OR carcinostatic OR "tumor inhibitor" OR "tumour inhibitor") W/3 (neuropath* OR neuralgia OR neurotox* OR mononeuropath* OR mono-neuropath* OR polyneuropath* OR poly-neuropath*)))) AND (((TITLE-ABS-KEY ((laboratory W/2 (marker OR markers OR values OR test* OR technique* OR service OR services OR diagnos* OR examin* OR evaluat*)) OR ("lab test" OR "lab tests" OR "lab result" OR "lab results" OR "lab value" OR "lab values" OR deficien*))) AND (TITLE-ABS-KEY (hemoglobin OR hemoglobins OR haemoglobin OR hemoglobine OR haemoglobine OR haemoglobins OR hemoglobulin OR hematocrit OR ferrohaemoglobin OR ferrohemoglobin OR erythrocytes OR eryhem OR albumin OR albumen OR prealbumin OR pre-albumin OR total-protein OR leptin OR globulin OR globulins OR polyglobulin OR poly-globulin OR choline OR bursine OR fagine OR vidine OR thiamin OR thiamine OR anemi* OR anaemi* OR aneurin OR riboflavin OR riboflavine OR "flavin mononucleotide" OR "flavin-adenine dinucleotide" OR niacin OR niacinamide OR enduramide OR nicobion OR nicotinamide OR nicotinsaureamid OR papulex OR "pantothenic acid" OR "calcium pantothenate" OR dexol OR "zinc pantothenate" OR "pyridoxal phosphate" OR "pyridoxal 5-phosphate" OR "pyridoxal-P" OR biotin OR folate OR "folic acid" OR folacin OR folvite OR folvit OR "pteroylglutamic acid" OR cobalamin OR cobalamins OR cyanocobalamin

© Mayo Clinic Libraries, Mayo Foundation for Medical Education and Research

OR eritron OR "B1" OR "B2" OR "B3" OR "B5" OR "B6" OR "B7" OR "B9" OR "B12" OR (b W/1 ("1" OR "2" OR "3" OR "5" OR "6" OR "7" OR "9" OR "12")) OR "methylmalonic acid" OR "methyl malonate" OR "methyl malonic acid" OR methylmalonate OR retinol OR carotene OR "Aquasol A" OR phytomenadione OR menaquinone OR menadione OR calcium OR chloride OR magnesium OR phosphorus OR romag OR potassium OR kalium OR sodium OR chromium OR copper OR fluoride OR iodine OR iron OR "Fe" OR "Fe+++" OR ferro OR ferrum OR manganese OR molybdenum OR selenium OR zinc))) OR (TITLE-ABS-KEY ((("complete blood" OR "red blood cell" OR erythrocyte OR rbc OR iron OR reticulocyte) W/1 (count OR indices)) OR "CBC" OR "hemogram" OR (("coronary risk" OR lipid OR cholesterol OR prothrombin OR prothrombine OR protrombin) W/1 (panel OR test)) OR thrombotest OR "Fe Tests")) OR (TITLE-ABS-KEY (undernutrition* OR malnutrition* OR avitaminosis OR ((nutrition* OR mineral* OR metabolic OR nutrient* OR micronutrient* OR micro-nutrient*) W/2 (deficien* OR diseases OR disorder*)))) OR (TITLE-ABS-KEY (beriberi OR pellagra OR scurvy OR rickets OR osteomalacia OR hypocalcem* OR hypo-calcem* OR hypercalcem* OR hyper-calcem* OR hypocalcaem* OR hypo-calcaem* OR hypercalcaem* OR hyper-calcaem* OR hypocalciur* OR hypo-calciur* OR hypercalciur* OR hyper-calciur* OR hypomagnesem* OR hypo-magnesem* OR hypermagnesem* OR hyper-magnesem* OR hypomagnesaem* OR hypo-magnesaem* OR hypermagnesaem* OR hyper-magnesaem* OR hypoalbuminem* OR hypo-albuminem* OR hyperalbuminem* OR hyper-albuminem* OR hypoalbuminaem* OR hypo-albuminaem* OR hyperalbuminaem* OR hyper-albuminaem* OR hypokalem* OR hypo-kalem* OR hyperkalem* OR hyper-kalem* OR hypokalaem* OR hypo-kalaem* OR hyperkalaem* OR hyper-kalaem* OR hypohomocysteinem* OR hypo-homocysteinem* OR hyperhomocysteinem* OR hyper-homocysteinem*)))) AND NOT ((PMID (0*) OR PMID (1*) OR PMID (2*) OR PMID (3*) OR PMID (4*) OR PMID (5*) OR PMID (6*) OR PMID (7*) OR PMID (8*) OR PMID (9*)))) AND NOT (TITLE-ABS (case W/1 (report* OR series)))) AND (TITLE-ABS (adult OR adults OR aged OR elderly))) AND (LIMIT-TO (PUBYEAR , 2021) OR LIMIT-TO (PUBYEAR , 2020))

6. **Web of Science** Core Collection: Citation Indexes

- **Science Citation Index Expanded (SCI-EXPANDED)** --1975-present

Data last updated: 2020-06-03 – searched June 4, 2020.

# 27	#26 OR #24<br>Indexes=SCI-EXPANDED Timespan=All years
# 26	#22 AND #25<br>Indexes=SCI-EXPANDED Timespan=All years
# 25	TS=(adult or adults or aged or elderly)<br>Indexes=SCI-EXPANDED Timespan=All years
# 24	#22 NOT #23<br>Indexes=SCI-EXPANDED Timespan=All years
# 23	TS=(infan* OR newborn* OR new-born* OR perinat* OR neonat* OR baby OR baby* OR babies OR toddler* OR minors OR mi

© Mayo Clinic Libraries, Mayo Foundation for Medical Education and Research

	nors* OR boy OR boys OR boyfriend OR boyhood OR girl* OR kid OR kids OR child OR child* OR children* OR schoolchild* OR schoolchild OR adolescen* OR juvenil* OR youth* OR teen* OR under*age* OR pubescen* OR pediatric* OR paediatric* OR peadiatric* OR prematur* OR preterm*OR school child OR "school child*" OR school OR school*)<br>Indexes=SCI-EXPANDED Timespan=All years
# 22	#21 AND #14<br>Indexes=SCI-EXPANDED Timespan=All years
# 21	#20 OR #19 OR #18 OR #17<br>Indexes=SCI-EXPANDED Timespan=All years
# 20	TS=(beriberi or pellagra or scurvy or rickets or osteomalacia or hypocalcem* or hypo-calcem* or hypercalcem* or hyper-calcem* or hypocalcaem* or hypo-calcaem* or hypercalcaem* or hyper-calcaem* or hypocalciur* or hypo-calciur* or hypercalciur* or hyper-calciur* or hypomagnesem* or hypo-magnesem* or hypermagnesem* or hyper-magnesem* or hypomagnesaem* or hypo-magnesaem* or hypermagnesaem* or hyper-magnesaem* or hypoalbuminem* or hypo-albuminem* or hyperalbuminem* or hyper-albuminem* or hypoalbuminaem* or hypo-albuminaem* or hyperalbuminaem* or hyper-albuminaem* or hypokalem* or hypo-kalem* or hyperkalem* or hyper-kalem* or hypokalaem* or hypo-kalaem* or hyperkalaem* or hyper-kalaem* or hypohomocysteinem* or hypo-homocysteinem* or hyperhomocysteinem* or hyper-homocysteinem*)<br>Indexes=SCI-EXPANDED Timespan=All years
# 19	TS=(undernutrition* or malnutrition* or avitaminosis or ((nutrition* or mineral* or metabolic or nutrient* or micronutrient* or micro-nutrient*) NEAR/2 (deficien* or diseases or disorder*)))<br>Indexes=SCI-EXPANDED Timespan=All years
# 18	TS=((("complete blood" or "red blood cell" or erythrocyte or RBC or iron or reticulocyte) NEAR/1 (count or indices)) or "CBC" or "hemogram" or (("coronary risk" or lipid or cholesterol or prothrombin or prothrombine or protrombin) NEAR/1 (panel or test)) or Thrombotest or "Fe Tests")<br>Indexes=SCI-EXPANDED Timespan=All years
# 17	#16 AND #15<br>Indexes=SCI-EXPANDED Timespan=All years
# 16	TS=(hemoglobin or hemoglobins or haemoglobin or hemoglobine or haemoglobine or haemoglobins or hemoglobulin or hematocrit or ferrohaemoglobin or ferrohemoglobin or erythrocytes or eryhem or albumin or albumen or prealbumin or pre-albumin or total-protein or leptin or globulin or globulins or polyglobulin or poly-globulin or choline or bursine or fagine or vidine or thiamin or thiamine or anemi* or anaemi* or aneurin or riboflavin or riboflavine or "flavin mononucleotide" or "flavin-adenine dinucleotide" or niacin or niacinamide or enduramide or nicobion or nicotinamide or nicotinsaureamid or papulex or "pantothenic acid" or "calcium pantothenate" or dexol or "zinc pantothenate" or "pyridoxal phosphate" or "pyridoxal 5-phosphate" or "pyridoxal-P" or biotin or folate or "folic acid" or folacin or folvite or folvit or "pteroylglutamic acid" or cobalamin or cobalamins or cyanocobalamin or eritron or "B1" or "B2" or "B3" or "B5" or "B6" or "B7" or "B9" or "B12" or (B NEAR/1 ("1" or "2" or "3" or "5" or "6" or "7" or "9" or "12")) or "methylmalonic acid" or "methyl malonate" or "methyl malonic acid" or methylmalonate or retinol or carotene or "Aquasol A" or phytomenadione or menaquinone or menadione or calcium or chloride or magnesium or phosphorus or romag or potassium or kalium or sodium or chromium or copper or fluoride or iodine or iron or "Fe" or "Fe+++" or ferro or ferrum or manganese or molybdenum or selenium or zinc)<br>Indexes=SCI-EXPANDED Timespan=All years
# 15	TS=((laboratory NEAR/2 (marker or markers or values or test* or technique* or service or services or diagnos* or examin* or evaluat*)) or ("lab test" or "lab tests" or "lab result" or "lab results" or "lab value" or "lab values" or deficien*))

© Mayo Clinic Libraries, Mayo Foundation for Medical Education and Research

	Indexes=SCI-EXPANDED Timespan=All years
# 14	#13 OR #12<br>Indexes=SCI-EXPANDED Timespan=All years
# 13	TS=((chemo* or antineoplas* or anti-neoplas* or anticancer* or anti-cancer* or anticarcino* or anti-carcino* or antitumor or anti-tumor or antitumour or anti-tumour or carcinochemo* or carcinostatic or "tumor inhibitor" or "tumour inhibitor") NEAR/3 (neuropath* or neuralgia or neurotox* or mononeuropath* or mono-neuropath* or polyneuropath* or poly-neuropath*))<br>Indexes=SCI-EXPANDED Timespan=All years
# 12	#11 AND #3<br>Indexes=SCI-EXPANDED Timespan=All years
# 11	#10 OR #9 OR #8 OR #7 OR #6 OR #5 OR #4<br>Indexes=SCI-EXPANDED Timespan=All years
# 10	TS=(antineoplastic or chemotherap* or chemo-therap* or ((anticancer* or anti-cancer* or anticarcino* or anti-carcino* or antitumor or anti-tumor or antitumour* or anti-tumour* or carcinostatic) NEAR/2 (therap* or agent or agents or treatment* or management*)) or carcinochemo* or "tumor inhibitor" or "tumour inhibitor")<br>Indexes=SCI-EXPANDED Timespan=All years
# 9	TS=("thalidomide" or "beta thalidomide" or "contergan" or "distaval" or "isomin" or "k 17" or "kedavon" or "kevadon" or "neurosedin" or "neurosedyne" or "nsc 66847" or "sedalis" or "shin naito" or "softenon" or "synovir" or "talimol" or "talizer" or "telagan" or "telargan" or "thado" or "thalidomid" or "thalidomide celgene" or "thalimodide" or "thalix" or "thalomid")<br>Indexes=SCI-EXPANDED Timespan=All years
# 8	TS=("bortezomib" or "ldp 341" or "ldp341" or "mg 341" or "mg341" or "mln 341" or "mln341" or "ps 341" or " ps341" or "velcade" or "vincristine" or "l 37231" or "l37231" or "vin cristine" or "vincristin" or "vincrisul" or "vinblastine" or "le 29060" or "le29060" or "leukoblastin" or "rozevin" or "vin blastine" or "vinblastin" or "vincaleucoblastin" or "vincaleucoblastine" or "vincaleukoblastine" or "vincoblastin" or "vincoblastine" or "vincoleucoblastine" or "vincoleukoblastin" or "vinleucoblastine")<br>Indexes=SCI-EXPANDED Timespan=All years
# 7	TS=("cisplatin" or "abiplatin" or "biocisplatinum" or "biocysplatinum" or "blastolem" or "briplatin" or "cddp ti" or "cis ddp" or "cis diamine dichloroplatinum" or "cis diaminechloroplatinum" or "cis diaminedichloroplatinum" or "cis diammine dichloroplatinum" or "cis diamminedichloroplatinum" or "cis dichloridiammineplatinum" or "cis dichloroadiamine platinum" or "cis dichlorodiamine platinum" or "cis dichlorodiammineplatinum" or "cis platinous diamino dichloride" or "cis platinum" or "cis platinum diamine dichloride" or "cis platinum diaminedichloride" or "cis platinum diamino dichloride" or "cis platinum diaminochloride" or "cis platinum diaminodichloride" or "cis platinum diammine dichloride" or "cis platinum diamminedichloride" or "cisplatin liposomal" or "cisplatin therapeutic implant" or "cisplatin-ebewe" or "cisplatine" or "cisplatino" or "cisplatinum" or "cisplatyl" or "citoplatino" or "cytoplatin" or "cytosplat" or "diamine dichloroplatinum" or "diaminodichloroplatinum" or "diamminedichloroplatinum" or "dichlorodiamine platinum" or "dichlorodiammineplatinum" or "docistin" or "elvecis" or "kemoplat" or "lederplatin" or "lipoplatin" or "liposomal cisplatin" or "mpi 5010" or "mpi5010" or "neoplatin" or "niyaplat" or "nk 801" or "noveldexis" or "nsc 119875" or "platamine" or "platamine rtu" or "platiblastin" or "platidiam" or "platimine" or "platinex" or "platinil" or "platinol" or "platinol aq" or "platinoxan" or "platinum diamine dichloride" or "platinum diaminedichloride" or "platinum diaminodichloride" or "platinum diamminedichloride" or "platiran" or "platistil" or "platistin" or "platosin" or "randa" or "romcis" or "sicatem" or "spi 077" or "tecnoplatin")<br>Indexes=SCI-EXPANDED Timespan=All years
# 6	TS=("oxaliplatin"

© Mayo Clinic Libraries, Mayo Foundation for Medical Education and Research

	or "axiplatin" or "crisapla" or "croloxat" or "dacotin" or "dacplat" or "ebeoxal" or "elatofen" or "eloxatin" or "eloxatine" or "elplat" or "geneplatin" or "heloxatin" or "lipoxal" or "mbp 426" or "mbp426" or "medoxa" or "oplat" or "oxalatoplatinum" or "oxalatplatin" or "oxali" or "oxalip" or "oxaliplan" or "oxaliplatina" or "oxaliprol" or "oxaliquid" or "oxalisan" or "oxalisin" or "oxalizo r" or "oxaltic" or "oxaltina" or "oxaplamyl" or "oxaviatin" or "platox" or "plaxitin" or "rectoxal" or "riboxatin" or "rp 54780" or "rp54780" or "sinoxal" or "sr 96669" or "sr96669" or "transplast in" or "velminox" or "xaliplat" or "xoplan")<br>Indexes=SCI-EXPANDED Timespan=All years
# 5	TS=("docetaxel" or "daxotel" or "dexotel" or "docefrez" or "docetaxel accord" or "lit 976" or "lit976" or "n debe nzoyl n tert butoxycarbonyl 10 deacetyltaxol" or "n tert butoxycarbonyl 10 deacetyl n debenzoylt axol" or "nsc 628503" or "nsc628503" or "oncodocel" or "rp 56976" or "rp56976" or "taxespira" or "taxoter" or "taxotere" or "texot")<br>Indexes=SCI-EXPANDED Timespan=All years
# 4	TS=("paclitaxel" or "abi 007" or "abi007" or "abraxane" or "albumin bound paclitaxel" or "anzatax" or "apealea" or "asotax" or "biotax" or "bms 181339" or "bms181339" or "bmy 45622" or "bmy45622" or "b ristaxol" or "britaxol" or "coroxane" or "dts 301" or "dts301" or "endotag-1" or "formoxol" or "genexol" or "genexol pm" or "hunxol" or "ifaxol" or "infinnium" or "intax el" or "mbt 0206" or "mbt0206" or "medixel" or "mitotax" or "nab paclitaxel" or "nanoparticle a lbumin bound paclitaxel" or "nsc 125973" or "nsc 673089" or "nsc125973" or "nsc673089" or "o as pac 100" or "oaspac100" or "oncogel" or "onxol" or "pacitaxel" or "paclitaxel nab" or "pacxe l" or "padexol" or "parexel" or "paxceed" or "paxene" or "paxus" or "praxel" or "taxocris" or "t axol" or "taycovit" or "yewtaxan")<br>Indexes=SCI-EXPANDED Timespan=All years
# 3	#2 OR #1<br>Indexes=SCI-EXPANDED Timespan=All years
# 2	TS=(PNS NEAR/2 (disease* or disorder*))<br>Indexes=SCI-EXPANDED Timespan=All years
# 1	TS=((peripheral NEAR/2 (nerve* or nervous) NEAR/2 (disease* or disorder*)) or "peripheral neuropath*")<br>Indexes=SCI-EXPANDED Timespan=All years

Update: **Web of Science Classic** Core Collection: **Science Citation Index Expanded** (SCI-EXPANDED) – 1975 to present.
Timespan: 2020-01-01 to 2030-12-31. Limited results to 2020 to present (searched on December 08, 2021). https://www.webofscience.com/wos/woscc/summary/fd7782bf-5730-4b8a-9ab4-18ded8223ad3-17f74533/relevance/1

# 23	#21 AND #14 and 2021 or 2020 (Publication Years)<br>| Exact search
# 22	#21 AND #14<br>| Exact search
# 21	#20 OR #19 OR #18 OR #17<br>| Exact search
# 20	TS=(beriberi or pellagra or scurvy or rickets or osteomalacia or hypocalcem* or hypo-calcem* or hypercalcem* or hyper-calcem* or hypocalcaem* or hypo-

© Mayo Clinic Libraries, Mayo Foundation for Medical Education and Research

	calcaem* or hypercalcaem* or hyper-calcaem* or hypocalciur* or hypo-calciur* or hypercalciur* or hyper-calciur* or hypomagnesem* or hypo-magnesem* or hypermagnesem* or hyper-magnesem* or hypomagnesaem* or hypo-magnesaem* or hypermagnesaem* or hyper-magnesaem* or hypoalbuminem* or hypo-albuminem* or hyperalbuminem* or hyper-albuminem* or hypoalbuminaem* or hypo-albuminaem* or hyperalbuminaem* or hyper-albuminaem* or hypokalem* or hypo-kalem* or hyperkalem* or hyper-kalem* or hypokalaem* or hypo-kalaem* or hyperkalaem* or hyper-kalaem* or hypohomocysteinem* or hypo-homocysteinem* or hyperhomocysteinem* or hyper-homocysteinem*) | Exact search
# 19	TS=(undernutrition* or malnutrition* or avitaminosis or ((nutrition* or mineral* or metabolic or nutrient* or micronutrient* or micro-nutrient*) NEAR/2 (deficien* or diseases or disorder*))) | Exact search
# 18	TS=((("complete blood" or "red blood cell" or erythrocyte or RBC or iron or reticulocyte) NEAR/1 (count or indices)) or "CBC" or "hemogram" or (("coronary risk" or lipid or cholesterol or prothrombin or prothrombine or protrombin) NEAR/1 (panel or test)) or Thrombotest or "Fe Tests") | Exact search
# 17	#16 AND #15 | Exact search
# 16	TS=(hemoglobin or hemoglobins or haemoglobin or hemoglobine or haemoglobine or haemoglobins or hemoglobulin or hematocrit or ferrohaemoglobin or ferrohemoglobin or erythrocytes or eryhem or albumin or albumen or prealbumin or pre-albumin or total-protein or leptin or globulin or globulins or polyglobulin or poly-globulin or choline or bursine or fagine or vidine or thiamin or thiamine or anemi* or anaemi* or aneurin or riboflavin or riboflavine or "flavin mononucleotide" or "flavin-adenine dinucleotide" or niacin or niacinamide or enduramide or nicobion or nicotinamide or nicotinsaureamid or papulex or "pantothenic acid" or "calcium pantothenate" or dexol or "zinc pantothenate" or "pyridoxal phosphate" or "pyridoxal 5-phosphate" or "pyridoxal-P" or biotin or folate or "folic acid" or folacin or folvite or folvit or "pteroylglutamic acid" or cobalamin or cobalamins or cyanocobalamin or eritron or "B1" or "B2" or "B3" or "B5" or "B6" or "B7" or "B9" or "B12" or (B NEAR/1 ("1" or "2" or "3" or "5" or "6" or "7" or "9" or "12")) or "methylmalonic acid" or "methyl malonate" or "methyl malonic acid" or methylmalonate or retinol or carotene or "Aquasol A" or phytomenadione or menaquinone or menadione or calcium or chloride or magnesium or phosphorus or romag or potassium or kalium or sodium or chromium or copper or fluoride or iodine or iron or "Fe" or "Fe+++" or ferro or ferrum or manganese or molybdenum or selenium or zinc) | Exact search
# 15	TS=((laboratory NEAR/2 (marker or markers or values or test* or technique* or service or services or diagnos* or examin* or evaluat*)) or ("lab test" or "lab tests" or "lab result" or "lab results" or "lab value" or "lab values" or deficien*)) | Exact search
# 14	#13 OR #12 | Exact search
# 13	TS=((chemo* or antineoplas* or anti-neoplas* or anticancer* or anti-cancer* or anticarcino* or anti-carcino* or antitumor or anti-tumor or antitumour or anti-tumour or carcinochemo* or carcinostatic or "tumor inhibitor" or "tumour inhibitor") NEAR/3 (neuropath* or neuralgia or neurotox* or mononeuropath* or mono-neuropath* or polyneuropath* or poly-neuropath*)) | Exact search
# 12	#11 AND #3 | Exact search
# 11	#10 OR #9 OR #8 OR #7 OR #6 OR #5 OR #4 | Exact search

© Mayo Clinic Libraries, Mayo Foundation for Medical Education and Research

# 10	TS=(antineoplastic or chemotherap* or chemo-therap* or ((anticancer* or anti-cancer* or anticarcino* or anti-carcino* or antitumor or anti-tumor or antitumour* or anti-tumour* or carcinostatic) NEAR/2 (therap* or agent or agents or treatment* or management*)) or carcinochemo* or "tumor inhibitor" or "tumour inhibitor")<br>| Exact search
# 9	TS=("thalidomide"<br>or "beta thalidomide" or "contergan" or "distaval" or "isomin" or "k 17" or "kedavon" or "kevad on" or "neurosedin" or "neurosedyne" or "nsc 66847" or "sedalis" or "shin naito" or "softenon" o r "synovir" or "talimol" or "talizer" or "telagan" or "telargan" or "thado" or "thalidomid" or "thal idomide celgene" or "thalimodide" or "thalix" or "thalomid")<br>| Exact search
# 8	TS=("bortezomib"<br>or "ldp 341" or "ldp341" or "mg 341" or "mg341" or "mln 341" or "mln341" or "ps 341" or "p s341" or "velcade" or "vincristine" or "l 37231" or "l37231" or "vin cristine" or "vincristin" or " vincrisul" or "vinblastine" or "le 29060" or "le29060" or "leukoblastin" or "rozevin" or "vin blast ine" or "vinblastin" or "vincaleucoblastin" or "vincaleucoblastine" or "vincaleukoblastine" or "vinc oblastin" or "vincoblastine" or "vincoleucoblastine" or "vincoleukoblastin" or "vinleucoblastine")<br>| Exact search
# 7	TS=("cisplatin"<br>or "abiplatin" or "biocisplatinum" or "biocysplatinum" or "blastolem" or "briplatin" or "cddp ti" o r "cis ddp" or "cis diamine dichloroplatinum" or "cis diaminechloroplatinum" or "cis diaminedichl oroplatinum" or "cis diammine dichloroplatinum" or "cis diamminedichloroplatinum" or "cis dichl oridiammineplatinum" or "cis dichloroadiamine platinum" or "cis dichlorodiamine platinum" or "ci s dichlorodiammineplatinum" or "cis platinous diamino dichloride" or "cis platinum" or "cis platin um diamine dichloride" or "cis platinum diaminedichloride" or "cis platinum diamino dichloride" or "cis platinum diaminochloride" or "cis platinum diaminodichloride" or "cis platinum diammine dichloride" or "cis platinum diamminedichloride" or "cisplatin liposomal" or "cisplatin therapeutic implant" or "cisplatin-<br>ebewe" or "cisplatine" or "cisplatino" or "cisplatinum" or "cisplatyl" or "citoplatino" or "cytoplati n" or "cytosplat" or "diamine dichloroplatinum" or "diaminodichloroplatinum" or "diamminedichlor oplatinum" or "dichlorodiamine platinum" or "dichlorodiammineplatinum" or "docistin" or "elvecis " or "kemoplat" or "lederplatin" or "lipoplatin" or "liposomal cisplatin" or "mpi 5010" or "mpi50 10" or "neoplatin" or "niyaplat" or "nk 801" or "noveldexis" or "nsc 119875" or "platamine" or "platamine rtu" or "platiblastin" or "platidiam" or "platimine" or "platinex" or "platinil" or "platin ol" or "platinol aq" or "platinoxan" or "platinum diamine dichloride" or "platinum diaminedichlori de" or "platinum diaminodichloride" or "platinum diamminedichloride" or "platiran" or "platistil" or "platistin" or "platosin" or "randa" or "romcis" or "sicatem" or "spi 077" or "tecnoplatin")<br>| Exact search
# 6	TS=("oxaliplatin"<br>or "axiplatin" or "crisapla" or "croloxat" or "dacotin" or "dacplat" or "ebeoxal" or "elatofen" or " eloxatin" or "eloxatine" or "elplat" or "geneplatin" or "heloxatin" or "lipoxal" or "mbp 426" or " mbp426" or "medoxa" or "oplat" or "oxalatoplatinum" or "oxalatplatin" or "oxali" or "oxalip" or "oxaliplan" or "oxaliplatina" or "oxaliprol" or "oxaliquid" or "oxalisan" or "oxalisin" or "oxalizor" or "oxaltic" or "oxaltina" or "oxaplamyl" or "oxaviatin" or "platox" or "plaxitin" or "rectoxal" o r "riboxatin" or "rp 54780" or "rp54780" or "sinoxal" or "sr 96669" or "sr96669" or "transplastin " or "velminox" or "xaliplat" or "xoplan")<br>| Exact search
# 5	TS=("docetaxel"<br>or "daxotel" or "dexotel" or "docefrez" or "docetaxel accord" or "lit 976" or "lit976" or "n debe nzoyl n tert butoxycarbonyl 10 deacetyltaxol" or "n tert butoxycarbonyl 10 deacetyl n debenzoylt axol" or "nsc 628503" or "nsc628503" or "oncodocel" or "rp 56976" or "rp56976" or "taxespira" or "taxoter" or "taxotere" or "texot")<br>| Exact search
# 4	TS=("paclitaxel"<br>or "abi 007" or "abi007" or "abraxane" or "albumin bound paclitaxel" or "anzatax" or "apealea" or "asotax" or "biotax" or "bms 181339" or "bms181339" or "bmy 45622" or "bmy45622" or "br

© Mayo Clinic Libraries, Mayo Foundation for Medical Education and Research

	istaxol" or "britaxol" or "coroxane" or "dts 301" or "dts301" or "endotag-1" or "formoxol" or "genexol" or "genexol pm" or "hunxol" or "ifaxol" or "infinnium" or "intaxel" or "mbt 0206" or "mbt0206" or "medixel" or "mitotax" or "nab paclitaxel" or "nanoparticle albumin bound paclitaxel" or "nsc 125973" or "nsc 673089" or "nsc125973" or "nsc673089" or "oas pac 100" or "oaspac100" or "oncogel" or "onxol" or "pacitaxel" or "paclitaxel nab" or "pacxel" or "padexol" or "parexel" or "paxceed" or "paxene" or "paxus" or "praxel" or "taxocris" or "taxol" or "taycovit" or "yewtaxan") | Exact search
3	#2 OR #1 | Exact search
2	TS=(PNS NEAR/2 (disease* or disorder*)) | Exact search
1	TS=((peripheral NEAR/2 (nerve* or nervous) NEAR/2 (disease* or disorder*)) or "peripheral neuropath*") | Exact search

7. **Web of Science** Core Collection: Citation Indexes

- **Emerging Sources Citation Index (ESCI)** --2015-present

Data last updated: 2020-06-03 – searched June 4, 2020.

# 27	#26 OR #24<br>Indexes=ESCI Timespan=All years
# 26	#22 AND #25<br>Indexes=ESCI Timespan=All years
# 25	TS=(adult or adults or aged or elderly)<br>Indexes=ESCI Timespan=All years
# 24	#22 NOT #23<br>Indexes=ESCI Timespan=All years
# 23	TS=(infan* OR newborn* OR new-born* OR perinat* OR neonat* OR baby OR baby* OR babies OR toddler* OR minors OR minors* OR boy OR boys OR boyfriend OR boyhood OR girl* OR kid OR kids OR child OR child* OR children* OR schoolchild* OR schoolchild OR adolescen* OR juvenil* OR youth* OR teen* OR under*age* OR pubescen* OR pediatric* OR paediatric* OR peadiatric* OR prematur* OR preterm*OR school child OR "school child*" OR school OR school*)<br>Indexes=ESCI Timespan=All years
# 22	#21 AND #14<br>Indexes=ESCI Timespan=All years
# 21	#20 OR #19 OR #18 OR #17<br>Indexes=ESCI Timespan=All years
# 20	TS=(beriberi or pellagra or scurvy or rickets or osteomalacia or hypocalcem* or hypo-calcem* or hypercalcem* or hyper-calcem* or hypocalcaem* or hypo-calcaem* or hypercalcaem* or hyper-calcaem* or hypocalciur* or hypo-calciur* or hypercalciur* or hyper-calciur* or hypomagnesem* or hypo-magnesem* or hypermagnesem* or hyper-magnesem* or hypomagnesaem* or hypo-

© Mayo Clinic Libraries, Mayo Foundation for Medical Education and Research

	magnesaem* or hypermagnesaem* or hyper-magnesaem* or hypoalbuminem* or hypo-albuminem* or hyperalbuminem* or hyper-albuminem* or hypoalbuminaem* or hypo-albuminaem* or hyperalbuminaem* or hyper-albuminaem* or hypokalem* or hypo-kalem* or hyperkalem* or hyper-kalem* or hypokalaem* or hypo-kalaem* or hyperkalaem* or hyper-kalaem* or hypohomocysteinem* or hypo-homocysteinem* or hyperhomocysteinem* or hyper-homocysteinem*)<br>Indexes=ESCI Timespan=All years
# 19	TS=(undernutrition* or malnutrition* or avitaminosis or ((nutrition* or mineral* or metabolic or nutrient* or micronutrient* or micro-nutrient*) NEAR/2 (deficien* or diseases or disorder*)))<br>Indexes=ESCI Timespan=All years
# 18	TS=((("complete blood" or "red blood cell" or erythrocyte or RBC or iron or reticulocyte) NEAR/1 (count or indices)) or "CBC" or "hemogram" or (("coronary risk" or lipid or cholesterol or prothrombin or prothrombine or protrombin) NEAR/1 (panel or test)) or Thrombotest or "Fe Tests")<br>Indexes=ESCI Timespan=All years
# 17	#16 AND #15<br>Indexes=ESCI Timespan=All years
# 16	TS=(hemoglobin or hemoglobins or haemoglobin or hemoglobine or haemoglobine or haemoglobins or hemoglobulin or hematocrit or ferrohaemoglobin or ferrohemoglobin or erythrocytes or eryhem or albumin or albumen or prealbumin or pre-albumin or total-protein or leptin or globulin or globulins or polyglobulin or poly-globulin or choline or bursine or fagine or vidine or thiamin or thiamine or anemi* or anaemi* or aneurin or riboflavin or riboflavine or "flavin mononucleotide" or "flavin-adenine dinucleotide" or niacin or niacinamide or enduramide or nicobion or nicotinamide or nicotinsaureamid or papulex or "pantothenic acid" or "calcium pantothenate" or dexol or "zinc pantothenate" or "pyridoxal phosphate" or "pyridoxal 5-phosphate" or "pyridoxal-P" or biotin or folate or "folic acid" or folacin or folvite or folvit or "pteroylglutamic acid" or cobalamin or cobalamins or cyanocobalamin or eritron or "B1" or "B2" or "B3" or "B5" or "B6" or "B7" or "B9" or "B12" or (B NEAR/1 ("1" or "2" or "3" or "5" or "6" or "7" or "9" or "12")) or "methylmalonic acid" or "methyl malonate" or "methyl malonic acid" or methylmalonate or retinol or carotene or "Aquasol A" or phytomenadione or menaquinone or menadione or calcium or chloride or magnesium or phosphorus or romag or potassium or kalium or sodium or chromium or copper or fluoride or iodine or iron or "Fe" or "Fe+++" or ferro or ferrum or manganese or molybdenum or selenium or zinc)<br>Indexes=ESCI Timespan=All years
# 15	TS=((laboratory NEAR/2 (marker or markers or values or test* or technique* or service or services or diagnos* or examin* or evaluat*)) or ("lab test" or "lab tests" or "lab result" or "lab results" or "lab value" or "lab values" or deficien*))<br>Indexes=ESCI Timespan=All years
# 14	#13 OR #12<br>Indexes=ESCI Timespan=All years
# 13	TS=((chemo* or antineoplas* or anti-neoplas* or anticancer* or anti-cancer* or anticarcino* or anti-carcino* or antitumor or anti-tumor or antitumour or anti-tumour or carcinochemo* or carcinostatic or "tumor inhibitor" or "tumour inhibitor") NEAR/3 (neuropath* or neuralgia or neurotox* or mononeuropath* or mono-neuropath* or polyneuropath* or poly-neuropath*))<br>Indexes=ESCI Timespan=All years
# 12	#11 AND #3

© Mayo Clinic Libraries, Mayo Foundation for Medical Education and Research

	Indexes=ESCI Timespan=All years
# 11	#10 OR #9 OR #8 OR #7 OR #6 OR #5 OR #4<br>Indexes=ESCI Timespan=All years
# 10	TS=(antineoplastic or chemotherap* or chemo-therap* or ((anticancer* or anti-cancer* or anticarcino* or anti-carcino* or antitumor or anti-tumor or antitumour* or anti-tumour* or carcinostatic) NEAR/2 (therap* or agent or agents or treatment* or management*)) or carcinochemo* or "tumor inhibitor" or "tumour inhibitor")<br>Indexes=ESCI Timespan=All years
# 9	TS=("thalidomide"<br>or "beta thalidomide" or "contergan" or "distaval" or "isomin" or "k 17" or "kedavon" or "kevad on" or "neurosedin" or "neurosedyne" or "nsc 66847" or "sedalis" or "shin naito" or "softenon" or "synovir" or "talimol" or "talizer" or "telagan" or "telargan" or "thado" or "thalidomid" or "th alidomide celgene" or "thalimodide" or "thalix" or "thalomid")<br>Indexes=ESCI Timespan=All years
# 8	TS=("bortezomib"<br>or "ldp 341" or "ldp341" or "mg 341" or "mg341" or "mln 341" or "mln341" or "ps 341" or " ps341" or "velcade" or "vincristine" or "l 37231" or "l37231" or "vin cristine" or "vincristin" or<br><br>"vincrisul" or "vinblastine" or "le 29060" or "le29060" or "leukoblastin" or "rozevin" or "vin bla stine" or "vinblastin" or "vincaleucoblastin" or "vincaleucoblastine" or "vincaleukoblastine" or "vin coblastin" or "vincoblastine" or "vincoleucoblastine" or "vincoleukoblastin" or "vinleucoblastine")<br>Indexes=ESCI Timespan=All years
# 7	TS=("cisplatin"<br>or "abiplatin" or "biocisplatinum" or "biocysplatinum" or "blastolem" or "briplatin" or "cddp ti" o r "cis ddp" or "cis diamine dichloroplatinum" or "cis diaminechloroplatinum" or "cis diaminedichl oroplatinum" or "cis diammine dichloroplatinum" or "cis diamminedichloroplatinum" or "cis dichl oridiammineplatinum" or "cis dichloroadiamine platinum" or "cis dichlorodiamine platinum" or "ci s dichlorodiammineplatinum" or "cis platinous diamino dichloride" or "cis platinum" or "cis plati num diamine dichloride" or "cis platinum diaminedichloride" or "cis platinum diamino dichloride" or "cis platinum diaminochloride" or "cis platinum diaminodichloride" or "cis platinum diammin e dichloride" or "cis platinum diamminedichloride" or "cisplatin liposomal" or "cisplatin therapeut ic implant" or "cisplatin-<br>ebewe" or "cisplatine" or "cisplatino" or "cisplatinum" or "cisplatyl" or "citoplatino" or "cytoplati n" or "cytosplat" or "diamine dichloroplatinum" or "diaminodichloroplatinum" or "diamminedichlor oplatinum" or "dichlorodiamine platinum" or "dichlorodiammineplatinum" or "docistin" or "elvecis " or "kemoplat" or "lederplatin" or "lipoplatin" or "liposomal cisplatin" or "mpi 5010" or "mpi50 10" or "neoplatin" or "niyaplat" or "nk 801" or "noveldexis" or "nsc 119875" or "platamine" or "platamine rtu" or "platiblastin" or "platidiam" or "platimine" or "platinex" or "platinil" or "platin ol" or "platinol aq" or "platinoxan" or "platinum diamine dichloride" or "platinum diaminedichlori de" or "platinum diaminodichloride" or "platinum diamminedichloride" or "platiran" or "platistil" or "platistin" or "platosin" or "randa" or "romcis" or "sicatem" or "spi 077" or "tecnoplatin")<br>Indexes=ESCI Timespan=All years
# 6	TS=("oxaliplatin"<br>or "axiplatin" or "crisapla" or "croloxat" or "dacotin" or "dacplat" or "ebeoxal" or "elatofen" or "eloxatin" or "eloxatine" or "elplat" or "geneplatin" or "heloxatin" or "lipoxal" or "mbp 426" or "mbp426" or "medoxa" or "oplat" or "oxalatoplatinum" or "oxalatplatin" or "oxali" or "oxalip" or "oxaliplan" or "oxaliplatina" or "oxaliprol" or "oxaliquid" or "oxalisan" or "oxalisin" or "oxalizo

© Mayo Clinic Libraries, Mayo Foundation for Medical Education and Research

	r" or "oxaltic" or "oxaltina" or "oxaplamyl" or "oxaviatin" or "platox" or "plaxitin" or "rectoxal" or "riboxatin" or "rp 54780" or "rp54780" or "sinoxal" or "sr 96669" or "sr96669" or "transplastin" or "velminox" or "xaliplat" or "xoplan")<br>Indexes=ESCI Timespan=All years
# 5	TS=("docetaxel"<br>or "daxotel" or "dexotel" or "docefrez" or "docetaxel accord" or "lit 976" or "lit976" or "n debenzoyl n tert butoxycarbonyl 10 deacetyltaxol" or "n tert butoxycarbonyl 10 deacetyl n debenzoyltaxol" or "nsc 628503" or "nsc628503" or "oncodocel" or "rp 56976" or "rp56976" or "taxespira" or "taxoter" or "taxotere" or "texot")<br>Indexes=ESCI Timespan=All years
# 4	TS=("paclitaxel"<br>or "abi 007" or "abi007" or "abraxane" or "albumin bound paclitaxel" or "anzatax" or "apealea" or "asotax" or "biotax" or "bms 181339" or "bms181339" or "bmy 45622" or "bmy45622" or "bristaxol" or "britaxol" or "coroxane" or "dts 301" or "dts301" or "endotag-1" or "formoxol" or "genexol" or "genexol pm" or "hunxol" or "ifaxol" or "infinnium" or "intaxel" or "mbt 0206" or "mbt0206" or "medixel" or "mitotax" or "nab paclitaxel" or "nanoparticle albumin bound paclitaxel" or "nsc 125973" or "nsc 673089" or "nsc125973" or "nsc673089" or "oas pac 100" or "oaspac100" or "oncogel" or "onxol" or "pacitaxel" or "paclitaxel nab" or "pacxel" or "padexol" or "parexel" or "paxceed" or "paxene" or "paxus" or "praxel" or "taxocris" or "taxol" or "taycovit" or "yewtaxan")<br>Indexes=ESCI Timespan=All years
# 3	#2 OR #1<br>Indexes=ESCI Timespan=All years
# 2	TS=(PNS NEAR/2 (disease* or disorder*))<br>Indexes=ESCI Timespan=All years
# 1	TS=((peripheral NEAR/2 (nerve* or nervous) NEAR/2 (disease* or disorder*)) or "peripheral neuropath*")<br>Indexes=ESCI Timespan=All years

Update: **Web of Science** Core Collection: **Emerging Sources Citation Index** (ESCI) --2015-present
Timespan: 2020-01-01 to 2030-12-31. Limited results to 2020 to present (searched onDecember 08, 2021).https://www.webofscience.com/wos/woscc/summary/2b719d27-efc4-49c1-9faf-1ecdf01bdbca-17f74876/relevance/1

# 23	#21 AND #14 and 2021 or 2020 (Publication Years)<br>| Exact search
# 22	#21 AND #14<br>| Exact search
# 21	#20 OR #19 OR #18 OR #17<br>| Exact search
# 20	TS=(beriberi or pellagra or scurvy or rickets or osteomalacia or hypocalcem* or hypo-calcem* or hypercalcem* or hyper-calcem* or hypocalcaem* or hypo-

© Mayo Clinic Libraries, Mayo Foundation for Medical Education and Research

	calcaem* or hypercalcaem* or hyper-calcaem* or hypocalciur* or hypo-calciur* or hypercalciur* or hyper-calciur* or hypomagnesem* or hypo-magnesem* or hypermagnesem* or hyper-magnesem* or hypomagnesaem* or hypo-magnesaem* or hypermagnesaem* or hyper-magnesaem* or hypoalbuminem* or hypo-albuminem* or hyperalbuminem* or hyper-albuminem* or hypoalbuminaem* or hypo-albuminaem* or hyperalbuminaem* or hyper-albuminaem* or hypokalem* or hypo-kalem* or hyperkalem* or hyper-kalem* or hypokalaem* or hypo-kalaem* or hyperkalaem* or hyper-kalaem* or hypohomocysteinem* or hypo-homocysteinem* or hyperhomocysteinem* or hyper-homocysteinem*) | Exact search
# 19	TS=(undernutrition* or malnutrition* or avitaminosis or ((nutrition* or mineral* or metabolic or nutrient* or micronutrient* or micro-nutrient*) NEAR/2 (deficien* or diseases or disorder*))) | Exact search
# 18	TS=((("complete blood" or "red blood cell" or erythrocyte or RBC or iron or reticulocyte) NEAR/1 (count or indices)) or "CBC" or "hemogram" or (("coronary risk" or lipid or cholesterol or prothrombin or prothrombine or protrombin) NEAR/1 (panel or test)) or Thrombotest or "Fe Tests") | Exact search
# 17	#16 AND #15 | Exact search
# 16	TS=(hemoglobin or hemoglobins or haemoglobin or hemoglobine or haemoglobine or haemoglobins or hemoglobulin or hematocrit or ferrohaemoglobin or ferrohemoglobin or erythrocytes or eryhem or albumin or albumen or prealbumin or pre-albumin or total-protein or leptin or globulin or globulins or polyglobulin or poly-globulin or choline or bursine or fagine or vidine or thiamin or thiamine or anemi* or anaemi* or aneurin or riboflavin or riboflavine or "flavin mononucleotide" or "flavin-adenine dinucleotide" or niacin or niacinamide or enduramide or nicobion or nicotinamide or nicotinsaureamid or papulex or "pantothenic acid" or "calcium pantothenate" or dexol or "zinc pantothenate" or "pyridoxal phosphate" or "pyridoxal 5-phosphate" or "pyridoxal-P" or biotin or folate or "folic acid" or folacin or folvite or folvit or "pteroylglutamic acid" or cobalamin or cobalamins or cyanocobalamin or eritron or "B1" or "B2" or "B3" or "B5" or "B6" or "B7" or "B9" or "B12" or (B NEAR/1 ("1" or "2" or "3" or "5" or "6" or "7" or "9" or "12")) or "methylmalonic acid" or "methyl malonate" or "methyl malonic acid" or methylmalonate or retinol or carotene or "Aquasol A" or phytomenadione or menaquinone or menadione or calcium or chloride or magnesium or phosphorus or romag or potassium or kalium or sodium or chromium or copper or fluoride or iodine or iron or "Fe" or "Fe+++" or ferro or ferrum or manganese or molybdenum or selenium or zinc) | Exact search
# 15	TS=((laboratory NEAR/2 (marker or markers or values or test* or technique* or service or services or diagnos* or examin* or evaluat*)) or ("lab test" or "lab tests" or "lab result" or "lab results" or "lab value" or "lab values" or deficien*)) | Exact search
# 14	#13 OR #12 | Exact search
# 13	TS=((chemo* or antineoplas* or anti-neoplas* or anticancer* or anti-cancer* or anticarcino* or anti-carcino* or antitumor or anti-tumor or antitumour or anti-tumour or carcinochemo* or carcinostatic or "tumor inhibitor" or "tumour inhibitor") NEAR/3 (neuropath* or neuralgia or neurotox* or mononeuropath* or mono-neuropath* or polyneuropath* or poly-neuropath*)) | Exact search
# 12	#11 AND #3 | Exact search
# 11	#10 OR #9 OR #8 OR #7 OR #6 OR #5 OR #4 | Exact search

© Mayo Clinic Libraries, Mayo Foundation for Medical Education and Research

# 10	TS=(antineoplastic or chemotherap* or chemo-therap* or ((anticancer* or anti-cancer* or anticarcino* or anti-carcino* or antitumor or anti-tumor or antitumour* or anti-tumour* or carcinostatic) NEAR/2 (therap* or agent or agents or treatment* or management*)) or carcinochemo* or "tumor inhibitor" or "tumour inhibitor") | Exact search
# 9	TS=("thalidomide" or "beta thalidomide" or "contergan" or "distaval" or "isomin" or "k 17" or "kedavon" or "kevad on" or "neurosedin" or "neurosedyne" or "nsc 66847" or "sedalis" or "shin naito" or "softenon" o r "synovir" or "talimol" or "talizer" or "telagan" or "telargan" or "thado" or "thalidomid" or "thal idomide celgene" or "thalimodide" or "thalix" or "thalomid") | Exact search
# 8	TS=("bortezomib" or "ldp 341" or "ldp341" or "mg 341" or "mg341" or "mln 341" or "mln341" or "ps 341" or "p s341" or "velcade" or "vincristine" or "l 37231" or "l37231" or "vin cristine" or "vincristin" or " vincrisul" or "vinblastine" or "le 29060" or "le29060" or "leukoblastin" or "rozevin" or "vin blast ine" or "vinblastin" or "vincaleucoblastin" or "vincaleucoblastine" or "vincaleukoblastine" or "vinc oblastin" or "vincoblastine" or "vincoleucoblastine" or "vincoleukoblastin" or "vinleucoblastine") | Exact search
# 7	TS=("cisplatin" or "abiplatin" or "biocisplatinum" or "biocysplatinum" or "blastolem" or "briplatin" or "cddp ti" o r "cis ddp" or "cis diamine dichloroplatinum" or "cis diaminechloroplatinum" or "cis diaminedichl oroplatinum" or "cis diammine dichloroplatinum" or "cis diamminedichloroplatinum" or "cis dichl oridiammineplatinum" or "cis dichloroadiamine platinum" or "cis dichlorodiamine platinum" or "ci s dichlorodiammineplatinum" or "cis platinous diamino dichloride" or "cis platinum" or "cis platin um diamine dichloride" or "cis platinum diaminedichloride" or "cis platinum diamino dichloride" or "cis platinum diaminochloride" or "cis platinum diaminodichloride" or "cis platinum diammine dichloride" or "cis platinum diamminedichloride" or "cisplatin liposomal" or "cisplatin therapeutic implant" or "cisplatin-ebewe" or "cisplatine" or "cisplatino" or "cisplatinum" or "cisplatyl" or "citoplatino" or "cytoplati n" or "cytosplat" or "diamine dichloroplatinum" or "diaminodichloroplatinum" or "diamminedichlor oplatinum" or "dichlorodiamine platinum" or "dichlorodiammineplatinum" or "docistin" or "elvecis " or "kemoplat" or "lederplatin" or "lipoplatin" or "liposomal cisplatin" or "mpi 5010" or "mpi50 10" or "neoplatin" or "niyaplat" or "nk 801" or "noveldexis" or "nsc 119875" or "platamine" or "platamine rtu" or "platiblastin" or "platidiam" or "platimine" or "platinex" or "platinil" or "platin ol" or "platinol aq" or "platinoxan" or "platinum diamine dichloride" or "platinum diaminedichlori de" or "platinum diaminodichloride" or "platinum diamminedichloride" or "platiran" or "platistil" or "platistin" or "platosin" or "randa" or "romcis" or "sicatem" or "spi 077" or "tecnoplatin") | Exact search
# 6	TS=("oxaliplatin" or "axiplatin" or "crisapla" or "croloxat" or "dacotin" or "dacplat" or "ebeoxal" or "elatofen" or " eloxatin" or "eloxatine" or "elplat" or "geneplatin" or "heloxatin" or "lipoxal" or "mbp 426" or " mbp426" or "medoxa" or "oplat" or "oxalatoplatinum" or "oxalatplatin" or "oxali" or "oxalip" or "oxaliplan" or "oxaliplatina" or "oxaliprol" or "oxaliquid" or "oxalisan" or "oxalisin" or "oxalizor" or "oxaltic" or "oxaltina" or "oxaplamyl" or "oxaviatin" or "platox" or "plaxitin" or "rectoxal" o r "riboxatin" or "rp 54780" or "rp54780" or "sinoxal" or "sr 96669" or "sr96669" or "transplastin " or "velminox" or "xaliplat" or "xoplan") | Exact search
# 5	TS=("docetaxel" or "daxotel" or "dexotel" or "docefrez" or "docetaxel accord" or "lit 976" or "lit976" or "n debe nzoyl n tert butoxycarbonyl 10 deacetyltaxol" or "n tert butoxycarbonyl 10 deacetyl n debenzoylt axol" or "nsc 628503" or "nsc628503" or "oncodocel" or "rp 56976" or "rp56976" or "taxespira" or "taxoter" or "taxotere" or "texot") | Exact search
# 4	TS=("paclitaxel" or "abi 007" or "abi007" or "abraxane" or "albumin bound paclitaxel" or "anzatax" or "apealea" or "asotax" or "biotax" or "bms 181339" or "bms181339" or "bmy 45622" or "bmy45622" or "br

© Mayo Clinic Libraries, Mayo Foundation for Medical Education and Research

	istaxol" or "britaxol" or "coroxane" or "dts 301" or "dts301" or "endotag-1" or "formoxol" or "genexol" or "genexol pm" or "hunxol" or "ifaxol" or "infinnium" or "intaxel" or "mbt 0206" or "mbt0206" or "medixel" or "mitotax" or "nab paclitaxel" or "nanoparticle albumin bound paclitaxel" or "nsc 125973" or "nsc 673089" or "nsc125973" or "nsc673089" or "oas pac 100" or "oaspac100" or "oncogel" or "onxol" or "pacitaxel" or "paclitaxel nab" or "pacxel" or "padexol" or "parexel" or "paxceed" or "paxene" or "paxus" or "praxel" or "taxocris" or "taxol" or "taycovit" or "yewtaxan")<br>| Exact search
3	#2 OR #1<br>| Exact search
2	TS=(PNS NEAR/2 (disease* or disorder*))<br>| Exact search
1	TS=((peripheral NEAR/2 (nerve* or nervous) NEAR/2 (disease* or disorder*)) or "peripheral neuropath*")<br>| Exact search

8. EBSCO*host* Research Databases - Cumulative Index to Nursing and Allied Health Literature (CINAHL with Full Text) 1963 – searched June0 4, 2020

Thursday, June 04, 2020 6:07:34 PM

#	Query	Limiters/Expanders	Last Run Via
S33	S30 OR S32	Expanders - Apply equivalent subjects<br>Search modes - Boolean/Phrase	Interface - EBSCOhost Research Databases<br>Search Screen - Advanced Search<br>Database - CINAHL with Full Text
S32	S28 AND S31	Expanders - Apply equivalent subjects<br>Search modes - Boolean/Phrase	Interface - EBSCOhost Research Databases<br>Search Screen - Advanced Search<br>Database - CINAHL with Full Text
S31	TI (adult or adults or aged or elderly) or AB (adult or adults or aged or elderly)	Expanders - Apply equivalent subjects<br>Search modes - Boolean/Phrase	Interface - EBSCOhost Research Databases<br>Search Screen - Advanced Search<br>Database - CINAHL with Full Text
S30	S28 NOT S29	Expanders - Apply equivalent subjects<br>Search modes - Boolean/Phrase	Interface - EBSCOhost Research Databases<br>Search Screen - Advanced Search<br>Database - CINAHL with Full Text
S29	TI (infan* OR newborn* OR new-born* OR	Expanders - Apply	Interface - EBSCOhost

© Mayo Clinic Libraries, Mayo Foundation for Medical Education and Research

	perinat* OR neonat* OR baby OR baby* OR babies OR toddler* OR minors OR minors* OR boy OR boys OR boyfriend OR boyhood OR girl* OR kid OR kids OR child OR child* OR children* OR schoolchild* OR schoolchild OR adolescen* OR juvenil* OR youth* OR teen* OR under*age* OR pubescen* OR pediatric* OR paediatric* OR peadiatric* OR prematur* OR preterm*OR school child OR "school child*" OR school OR school*) or AB (infan* OR newborn* OR new-born* OR perinat* OR neonat* OR baby OR baby* OR babies OR toddler* OR minors OR minors* OR boy OR boys OR boyfriend OR boyhood OR girl* OR kid OR kids OR child OR child* OR children* OR schoolchild* OR schoolchild OR adolescen* OR juvenil* OR youth* OR teen* OR under*age* OR pubescen* OR pediatric* OR paediatric* OR peadiatric* OR prematur* OR preterm*OR school child OR "school child*" OR school OR school*)	equivalent subjects<br>Search modes - Boolean/Phrase	Research Databases<br>Search Screen - Advanced Search<br>Database - CINAHL with Full Text
S28	S26 NOT S27	Expanders - Apply equivalent subjects<br>Search modes - Boolean/Phrase	Interface - EBSCOhost Research Databases<br>Search Screen - Advanced Search<br>Database - CINAHL with Full Text
S27	TI (rat or rats or mouse or mice) or AB (rat or rats or mouse or mice)	Expanders - Apply equivalent subjects<br>Search modes - Boolean/Phrase	Interface - EBSCOhost Research Databases<br>Search Screen - Advanced Search<br>Database - CINAHL with Full Text
S26	S24 NOT S25	Expanders - Apply equivalent subjects<br>Search modes - Boolean/Phrase	Interface - EBSCOhost Research Databases<br>Search Screen - Advanced Search<br>Database - CINAHL with Full Text
S25	TI (case N1 (report* or series)) or AB (case N1 (report* or series))	Expanders - Apply equivalent subjects<br>Search modes - Boolean/Phrase	Interface - EBSCOhost Research Databases<br>Search Screen - Advanced Search<br>Database - CINAHL with Full Text
S24	S11 AND S23	Expanders - Apply equivalent subjects<br>Search modes - Boolean/Phrase	Interface - EBSCOhost Research Databases<br>Search Screen - Advanced Search<br>Database - CINAHL with Full Text

© Mayo Clinic Libraries, Mayo Foundation for Medical Education and Research

S23	S18 OR S19 OR S20 OR S21 OR S22	Expanders - Apply equivalent subjects<br>Search modes - Boolean/Phrase	Interface - EBSCOhost Research Databases<br>Search Screen - Advanced Search<br>Database - CINAHL with Full Text
S22	TI (beriberi or pellagra or scurvy or rickets or osteomalacia or hypocalcem* or hypo-calcem* or hypercalcem* or hyper-calcem* or hypocalcaem* or hypo-calcaem* or hypercalcaem* or hyper-calcaem* or hypocalciur* or hypo-calciur* or hypercalciur* or hyper-calciur* or hypomagnesem* or hypo-magnesem* or hypermagnesem* or hyper-magnesem* or hypomagnesaem* or hypo-magnesaem* or hypermagnesaem* or hyper-magnesaem* or hypoalbuminem* or hypo-albuminem* or hyperalbuminem* or hyper-albuminem* or hypoalbuminaem* or hypo-albuminaem* or hyperalbuminaem* or hyper-albuminaem* or hypokalem* or hypo-kalem* or hyperkalem* or hyper-kalem* or hypokalaem* or hypo-kalaem* or hyperkalaem* or hyper-kalaem* or hyperhomocysteinemia or hyper-homocysteinemia) or AB (beriberi or pellagra or scurvy or rickets or osteomalacia or hypocalcem* or hypo-calcem* or hypercalcem* or hyper-calcem* or hypocalcaem* or hypo-calcaem* or hypercalcaem* or hyper-calcaem* or hypocalciur* or hypo-calciur* or hypercalciur* or hyper-calciur* or hypomagnesem* or hypo-magnesem* or hypermagnesem* or hyper-magnesem* or hypomagnesaem* or hypo-magnesaem* or hypermagnesaem* or hyper-magnesaem* or hypoalbuminem* or hypo-albuminem* or hyperalbuminem* or hyper-albuminem* or hypoalbuminaem* or hypo-albuminaem* or hyperalbuminaem* or hyper-albuminaem* or hypokalem* or hypo-kalem* or hyperkalem* or hyper-kalem* or hypokalaem* or hypo-kalaem* or hyperkalaem* or hyper-kalaem* or hyperhomocysteinemia or hyper-homocysteinemia)	Expanders - Apply equivalent subjects<br>Search modes - Boolean/Phrase	Interface - EBSCOhost Research Databases<br>Search Screen - Advanced Search<br>Database - CINAHL with Full Text
S21	TI (undernutrition* or malnutrition* or avitaminosis or ((nutrition* or mineral* or metabolic or nutrient* or micronutrient* or micro-nutrient*) N2 (deficien* or diseases or disorder*))) or AB (undernutrition* or malnutrition* or avitaminosis or ((nutrition* or mineral* or metabolic or nutrient* or micronutrient* or micro-nutrient*) N2 (deficien* or diseases or disorder*))) or AB	Expanders - Apply equivalent subjects<br>Search modes - Boolean/Phrase	Interface - EBSCOhost Research Databases<br>Search Screen - Advanced Search<br>Database - CINAHL with Full Text

© Mayo Clinic Libraries, Mayo Foundation for Medical Education and Research

	(undernutrition* or malnutrition* or avitaminosis or ((nutrition* or mineral* or metabolic or nutrient* or micronutrient* or micro-nutrient*) N2 (deficien* or diseases or disorder*)))		
S20	(MH "Nutritional and Metabolic Diseases+") NOT (MH "Obesity+")	Expanders - Apply equivalent subjects<br>Search modes - Boolean/Phrase	Interface - EBSCOhost Research Databases<br>Search Screen - Advanced Search<br>Database - CINAHL with Full Text
S19	TI ((("complete blood" or "red blood cell" or erythrocyte or RBC or iron or reticulocyte) N1 (count or indices)) or "CBC" or "hemogram" or (("coronary risk" or lipid or cholesterol or prothrombin or prothrombine or protrombin) N1 (panel or test)) or Thrombotest or "Fe Tests") or AB ((("complete blood" or "red blood cell" or erythrocyte or RBC or iron or reticulocyte) N1 (count or indices)) or "CBC" or "hemogram" or (("coronary risk" or lipid or cholesterol or prothrombin or prothrombine or protrombin) N1 (panel or test)) or Thrombotest or "Fe Tests")	Expanders - Apply equivalent subjects<br>Search modes - Boolean/Phrase	Interface - EBSCOhost Research Databases<br>Search Screen - Advanced Search<br>Database - CINAHL with Full Text
S18	S14 AND S17	Expanders - Apply equivalent subjects<br>Search modes - Boolean/Phrase	Interface - EBSCOhost Research Databases<br>Search Screen - Advanced Search<br>Database - CINAHL with Full Text
S17	S15 OR S16	Expanders - Apply equivalent subjects<br>Search modes - Boolean/Phrase	Interface - EBSCOhost Research Databases<br>Search Screen - Advanced Search<br>Database - CINAHL with Full Text
S16	TI (hemoglobin or hemoglobins or haemoglobin or hemoglobine or haemoglobine or haemoglobins or hemoglobulin or hematocrit or ferrohaemoglobin or ferrohemoglobin or erythrocytes or eryhem or albumin or albumen or prealbumin or pre-albumin or total-protein or leptin or globulin or globulins or polyglobulin or poly-globulin or choline or bursine or fagine or vidine or thiamin or thiamine or anemi* or anaemi* or aneurin or riboflavin or riboflavine or "flavin mononucleotide" or "flavin-adenine dinucleotide" or niacin or niacinamide or enduramide or nicobion or nicotinamide or nicotinsaureamid or papulex or "pantothenic acid" or "calcium pantothenate" or dexol or "zinc pantothenate" or "pyridoxal phosphate" or	Expanders - Apply equivalent subjects<br>Search modes - Boolean/Phrase	Interface - EBSCOhost Research Databases<br>Search Screen - Advanced Search<br>Database - CINAHL with Full Text

© Mayo Clinic Libraries, Mayo Foundation for Medical Education and Research

	"pyridoxal 5-phosphate" or "pyridoxal-P" or biotin or folate or "folic acid" or folacin or folvite or folvit or "pteroylglutamic acid" or cobalamin or cobalamins or cyanocobalamin or eritron or "B1" or "B2" or "B3" or "B5" or "B6" or "B7" or "B9" or "B12" or (B N ("1" or "2" or "3" or "5" or "6" or "7" or "9" or "12")) or "methylmalonic acid" or "methyl malonate" or "methyl malonic acid" or methylmalonate or retinol or carotene or "Aquasol A" or phytomenadione or menaquinone or menadione or calcium or chloride or magnesium or phosphorus or romag or potassium or kalium or sodium or chromium or copper or fluoride or iodine or iron or "Fe" or "Fe+++" or ferro or ferrum or manganese or molybdenum or selenium or zinc) or AB (hemoglobin or hemoglobins or haemoglobin or hemoglobine or haemoglobine or haemoglobins or hemoglobulin or hematocrit or ferrohaemoglobin or ferrohemoglobin or erythrocytes or eryhem or albumin or albumen or prealbumin or pre-albumin or total-protein or leptin or globulin or globulins or polyglobulin or poly-globulin or choline or bursine or fagine or vidine or thiamin or thiamine or anemi* or anaemi* or aneurin or riboflavin or riboflavine or "flavin mononucleotide" or "flavin-adenine dinucleotide" or niacin or niacinamide or enduramide or nicobion or nicotinamide or nicotinsaureamid or papulex or "pantothenic acid" or "calcium pantothenate" or dexol or "zinc pantothenate" or "pyridoxal phosphate" or "pyridoxal 5-phosphate" or "pyridoxal-P" or biotin or folate or "folic acid" or folacin or folvite or folvit or "pteroylglutamic acid" or cobalamin or cobalamins or cyanocobalamin or eritron or "B1" or "B2" or "B3" or "B5" or "B6" or "B7" or "B9" or "B12" or (B N ("1" or "2" or "3" or "5" or "6" or "7" or "9" or "12")) or "methylmalonic acid" or "methyl malonate" or "methyl malonic acid" or methylmalonate or retinol or carotene or "Aquasol A" or phytomenadione or menaquinone or menadione or calcium or chloride or magnesium or phosphorus or romag or potassium or kalium or sodium or chromium or copper or fluoride or iodine or iron or "Fe" or "Fe+++" or ferro or ferrum or manganese or molybdenum or selenium or zinc)		
S15	(MH "Hemoglobins") or (MH "Anemia") or (MH "Homocysteine") or (MH "Nutrients+") or (MH "Minerals+") or (MH "Vitamin A+") or (MH "Riboflavin") or (MH "Niacin") or (MH	Expanders - Apply equivalent subjects Search modes - Boolean/Phrase	Interface - EBSCOhost Research Databases Search Screen - Advanced Search

© Mayo Clinic Libraries, Mayo Foundation for Medical Education and Research

	"Vitamin B Complex+") or (MH "Ascorbic Acid") or (MH "Vitamin D") or (MH "Vitamin E") or (MH "Vitamin K") or (MH "Prothrombin Time") or (MH "Calcium") or (MH "Magnesium") or (MH "Potassium") or (MH "Sodium, Dietary+") or (MH "Sodium") or (MH "Chromium") or (MH "Fluorides") or (MH "Iodine") or (MH "Iron") or (MH "Manganese") or (MH "Molybdenum") or (MH "Selenium") or (MH "Zinc")		Database - CINAHL with Full Text
S14	S12 OR S13	Expanders - Apply equivalent subjects<br>Search modes - Boolean/Phrase	Interface - EBSCOhost Research Databases<br>Search Screen - Advanced Search<br>Database - CINAHL with Full Text
S13	TI ((laboratory N2 (marker or markers or values or test* or technique* or service or services or diagnos* or examin* or evaluat*)) or ("lab test" or "lab tests" or "lab result" or "lab results" or "lab value" or "lab values" or deficien*)) or AB ((laboratory N2 (marker or markers or values or test* or technique* or service or services or diagnos* or examin* or evaluat*)) or ("lab test" or "lab tests" or "lab result" or "lab results" or "lab value" or "lab values" or deficien*))	Expanders - Apply equivalent subjects<br>Search modes - Boolean/Phrase	Interface - EBSCOhost Research Databases<br>Search Screen - Advanced Search<br>Database - CINAHL with Full Text
S12	(MH "Clinical Laboratories+")	Expanders - Apply equivalent subjects<br>Search modes - Boolean/Phrase	Interface - EBSCOhost Research Databases<br>Search Screen - Advanced Search<br>Database - CINAHL with Full Text
S11	S3 OR S9 OR S10	Expanders - Apply equivalent subjects<br>Search modes - Boolean/Phrase	Interface - EBSCOhost Research Databases<br>Search Screen - Advanced Search<br>Database - CINAHL with Full Text
S10	TI ((chemo* or antineoplas* or anti-neoplas* or anticancer* or anti-cancer* or anticarcino* or anti-carcino* or antitumor or anti-tumor or antitumour or anti-tumour or carcinochemo* or carcinostatic or "tumor inhibitor" or "tumour inhibitor") N3 (neuropath* or neuralgia or neurotox* or mononeuropath* or mono-neuropath* or polyneuropath* or poly-neuropath*)) or AB ((chemo* or antineoplas* or anti-neoplas* or anticancer* or anti-cancer* or anticarcino* or anti-carcino* or antitumor or anti-tumor or antitumour or anti-tumour or carcinochemo* or carcinostatic or "tumor	Expanders - Apply equivalent subjects<br>Search modes - Boolean/Phrase	Interface - EBSCOhost Research Databases<br>Search Screen - Advanced Search<br>Database - CINAHL with Full Text

© Mayo Clinic Libraries, Mayo Foundation for Medical Education and Research

	inhibitor" or "tumour inhibitor") N3 (neuropath* or neuralgia or neurotox* or mononeuropath* or mono-neuropath* or polyneuropath* or poly-neuropath*))		
S9	S7 AND S8	Expanders - Apply equivalent subjects<br>Search modes - Boolean/Phrase	Interface - EBSCOhost Research Databases<br>Search Screen - Advanced Search<br>Database - CINAHL with Full Text
S8	(MH "Antineoplastic Agents+") or (MH "Chemotherapy, Adjuvant+") or TI ((anticancer* or anti-cancer* or anticarcino* or anti-carcino* or antitumor or anti-tumor or antitumour* or anti-tumour* or carcinostatic) N2 (therap* or agent or agents or treatment* or management*)) or carcinochemo* or "tumor inhibitor" or "tumour inhibitor") or AB ((anticancer* or anti-cancer* or anticarcino* or anti-carcino* or antitumor or anti-tumor or antitumour* or anti-tumour* or carcinostatic) N2 (therap* or agent or agents or treatment* or management*)) or carcinochemo* or "tumor inhibitor" or "tumour inhibitor")	Expanders - Apply equivalent subjects<br>Search modes - Boolean/Phrase	Interface - EBSCOhost Research Databases<br>Search Screen - Advanced Search<br>Database - CINAHL with Full Text
S7	S4 OR S5 OR S6	Expanders - Apply equivalent subjects<br>Search modes - Boolean/Phrase	Interface - EBSCOhost Research Databases<br>Search Screen - Advanced Search<br>Database - CINAHL with Full Text
S6	TI (PNS N2 (disease* or disorder*)) or AB (PNS N2 (disease* or disorder*))	Expanders - Apply equivalent subjects<br>Search modes - Boolean/Phrase	Interface - EBSCOhost Research Databases<br>Search Screen - Advanced Search<br>Database - CINAHL with Full Text
S5	TI ((peripheral N2 (nerve* or nervous) N2 (disease* or disorder*)) or "peripheral neuropath*") or AB ((peripheral N2 (nerve* or nervous) N2 (disease* or disorder*)) or "peripheral neuropath*")	Expanders - Apply equivalent subjects<br>Search modes - Boolean/Phrase	Interface - EBSCOhost Research Databases<br>Search Screen - Advanced Search<br>Database - CINAHL with Full Text
S4	(MH "Peripheral Nervous System Diseases+")	Expanders - Apply equivalent subjects<br>Search modes - Boolean/Phrase	Interface - EBSCOhost Research Databases<br>Search Screen - Advanced Search<br>Database - CINAHL with Full Text
S3	S1 OR S2	Expanders - Apply	Interface - EBSCOhost

© Mayo Clinic Libraries, Mayo Foundation for Medical Education and Research

		equivalent subjects<br>Search modes - Boolean/Phrase	Research Databases<br>Search Screen - Advanced Search<br>Database - CINAHL with Full Text
S2	(MH "Neuralgia+/CI")	Expanders - Apply equivalent subjects<br>Search modes - Boolean/Phrase	Interface - EBSCOhost Research Databases<br>Search Screen - Advanced Search<br>Database - CINAHL with Full Text
S1	(MH "Peripheral Nervous System Diseases+/CI")	Expanders - Apply equivalent subjects<br>Search modes - Boolean/Phrase	Interface - EBSCOhost Research Databases<br>Search Screen - Advanced Search<br>Database - CINAHL with Full Text

Update: **EBSCO*host* Research Databases - Cumulative Index to Nursing and Allied Health Literature (CINAHL with Full Text)** 1963 – searched December 08, 2021

Wednesday, December 08, 2021 10:56:20 PM

#	Query	Limiters/Expanders	Last Run Via
S25	S11 AND S23	Limiters - Published Date: 20200101-20301231<br>Expanders - Apply equivalent subjects<br>Search modes - Boolean/Phrase	Interface - EBSCOhost Research Databases<br>Search Screen - Basic Search<br>Database - CINAHL with Full Text
S24	S11 AND S23	Expanders - Apply equivalent subjects<br>Search modes - Boolean/Phrase	Interface - EBSCOhost Research Databases<br>Search Screen - Advanced Search<br>Database - CINAHL with Full Text
S23	S18 OR S19 OR S20 OR S21 OR S22	Expanders - Apply equivalent subjects<br>Search modes - Boolean/Phrase	Interface - EBSCOhost Research Databases<br>Search Screen - Advanced Search<br>Database - CINAHL with Full Text
S22	TI (beriberi or pellagra or scurvy or rickets or osteomalacia or hypocalcem* or hypo-calcem* or hypercalcem* or hyper-calcem* or hypocalcaem* or hypo-calcaem* or hypercalcaem* or hyper-calcaem* or hypocalciur* or hypo-calciur* or hypercalciur* or hyper-calciur* or hypomagnesem* or hypo-magnesem* or hypermagnesem* or hyper-magnesem* or hypomagnesaem* or hypo-	Expanders - Apply equivalent subjects<br>Search modes - Boolean/Phrase	Interface - EBSCOhost Research Databases<br>Search Screen - Advanced Search<br>Database - CINAHL with Full Text

© Mayo Clinic Libraries, Mayo Foundation for Medical Education and Research

	magnesaem* or hypermagnesaem* or hyper-magnesaem* or hypoalbuminem* or hypo-albuminem* or hyperalbuminem* or hyper-albuminem* or hypoalbuminaem* or hypo-albuminaem* or hyperalbuminaem* or hyper-albuminaem* or hypokalem* or hypo-kalem* or hyperkalem* or hyper-kalem* or hypokalaem* or hypo-kalaem* or hyperkalaem* or hyper-kalaem* or hyperhomocysteinemia or hyper-homocysteinemia) or AB (beriberi or pellagra or scurvy or rickets or osteomalacia or hypocalcem* or hypo-calcem* or hypercalcem* or hyper-calcem* or hypocalcaem* or hypo-calcaem* or hypercalcaem* or hyper-calcaem* or hypocalciur* or hypo-calciur* or hypercalciur* or hyper-calciur* or hypomagnesem* or hypo-magnesem* or hypermagnesem* or hyper-magnesem* or hypomagnesaem* or hypo-magnesaem* or hypermagnesaem* or hyper-magnesaem* or hypoalbuminem* or hypo-albuminem* or hyperalbuminem* or hyper-albuminem* or hypoalbuminaem* or hypo-albuminaem* or hyperalbuminaem* or hyper-albuminaem* or hypokalem* or hypo-kalem* or hyperkalem* or hyper-kalem* or hypokalaem* or hypo-kalaem* or hyperkalaem* or hyper-kalaem* or hyperhomocysteinemia or hyper-homocysteinemia)		
S21	TI (undernutrition* or malnutrition* or avitaminosis or ((nutrition* or mineral* or metabolic or nutrient* or micronutrient* or micro-nutrient*) N2 (deficien* or diseases or disorder*))) or AB (undernutrition* or malnutrition* or avitaminosis or ((nutrition* or mineral* or metabolic or nutrient* or micronutrient* or micro-nutrient*) N2 (deficien* or diseases or disorder*))) or AB (undernutrition* or malnutrition* or avitaminosis or ((nutrition* or mineral* or metabolic or nutrient* or micronutrient* or micro-nutrient*) N2 (deficien* or diseases or disorder*)))	Expanders - Apply equivalent subjects<br>Search modes - Boolean/Phrase	Interface - EBSCOhost Research Databases<br>Search Screen - Advanced Search<br>Database - CINAHL with Full Text
S20	(MH "Nutritional and Metabolic Diseases+") NOT (MH "Obesity+")	Expanders - Apply equivalent subjects<br>Search modes - Boolean/Phrase	Interface - EBSCOhost Research Databases<br>Search Screen - Advanced Search<br>Database - CINAHL with Full Text
S19	TI ((("complete blood" or "red blood cell" or erythrocyte or RBC or iron or reticulocyte) N1 (count or indices)) or "CBC" or "hemogram" or (("coronary risk" or lipid or cholesterol or prothrombin or prothrombine or protrombin)	Expanders - Apply equivalent subjects<br>Search modes - Boolean/Phrase	Interface - EBSCOhost Research Databases<br>Search Screen - Advanced Search<br>Database - CINAHL with Full

© Mayo Clinic Libraries, Mayo Foundation for Medical Education and Research

	N1 (panel or test)) or Thrombotest or "Fe Tests") or AB ((("complete blood" or "red blood cell" or erythrocyte or RBC or iron or reticulocyte) N1 (count or indices)) or "CBC" or "hemogram" or (("coronary risk" or lipid or cholesterol or prothrombin or prothrombine or protrombin) N1 (panel or test)) or Thrombotest or "Fe Tests")		Text
S18	S14 AND S17	Expanders - Apply equivalent subjects<br>Search modes - Boolean/Phrase	Interface - EBSCOhost Research Databases<br>Search Screen - Advanced Search<br>Database - CINAHL with Full Text
S17	S15 OR S16	Expanders - Apply equivalent subjects<br>Search modes - Boolean/Phrase	Interface - EBSCOhost Research Databases<br>Search Screen - Advanced Search<br>Database - CINAHL with Full Text
S16	TI (hemoglobin or hemoglobins or haemoglobin or hemoglobine or haemoglobine or haemoglobins or hemoglobulin or hematocrit or ferrohaemoglobin or ferrohemoglobin or erythrocytes or eryhem or albumin or albumen or prealbumin or pre-albumin or total-protein or leptin or globulin or globulins or polyglobulin or poly-globulin or choline or bursine or fagine or vidine or thiamin or thiamine or anemi* or anaemi* or aneurin or riboflavin or riboflavine or "flavin mononucleotide" or "flavin-adenine dinucleotide" or niacin or niacinamide or enduramide or nicobion or nicotinamide or nicotinsaureamid or papulex or "pantothenic acid" or "calcium pantothenate" or dexol or "zinc pantothenate" or "pyridoxal phosphate" or "pyridoxal 5-phosphate" or "pyridoxal-P" or biotin or folate or "folic acid" or folacin or folvite or folvit or "pteroylglutamic acid" or cobalamin or cobalamins or cyanocobalamin or eritron or "B1" or "B2" or "B3" or "B5" or "B6" or "B7" or "B9" or "B12" or (B N ("1" or "2" or "3" or "5" or "6" or "7" or "9" or "12")) or "methylmalonic acid" or "methyl malonate" or "methyl malonic acid" or methylmalonate or retinol or carotene or "Aquasol A" or phytomenadione or menaquinone or menadione or calcium or chloride or magnesium or phosphorus or romag or potassium or kalium or sodium or chromium or copper or fluoride or iodine or iron or "Fe" or "Fe+++" or ferro or ferrum or manganese or molybdenum or selenium or zinc) or AB (hemoglobin or hemoglobins or haemoglobin or hemoglobine or haemoglobine or haemoglobins or hemoglobulin or hematocrit or	Expanders - Apply equivalent subjects<br>Search modes - Boolean/Phrase	Interface - EBSCOhost Research Databases<br>Search Screen - Advanced Search<br>Database - CINAHL with Full Text

© Mayo Clinic Libraries, Mayo Foundation for Medical Education and Research

	ferrohaemoglobin or ferrohemoglobin or erythrocytes or eryhem or albumin or albumen or prealbumin or pre-albumin or total-protein or leptin or globulin or globulins or polyglobulin or poly-globulin or choline or bursine or fagine or vidine or thiamin or thiamine or anemi* or anaemi* or aneurin or riboflavin or riboflavine or "flavin mononucleotide" or "flavin-adenine dinucleotide" or niacin or niacinamide or enduramide or nicobion or nicotinamide or nicotinsaureamid or papulex or "pantothenic acid" or "calcium pantothenate" or dexol or "zinc pantothenate" or "pyridoxal phosphate" or "pyridoxal 5-phosphate" or "pyridoxal-P" or biotin or folate or "folic acid" or folacin or folvite or folvit or "pteroylglutamic acid" or cobalamin or cobalamins or cyanocobalamin or eritron or "B1" or "B2" or "B3" or "B5" or "B6" or "B7" or "B9" or "B12" or (B N ("1" or "2" or "3" or "5" or "6" or "7" or "9" or "12")) or "methylmalonic acid" or "methyl malonate" or "methyl malonic acid" or methylmalonate or retinol or carotene or "Aquasol A" or phytomenadione or menaquinone or menadione or calcium or chloride or magnesium or phosphorus or romag or potassium or kalium or sodium or chromium or copper or fluoride or iodine or iron or "Fe" or "Fe+++" or ferro or ferrum or manganese or molybdenum or selenium or zinc)		
S15	(MH "Hemoglobins") or (MH "Anemia") or (MH "Homocysteine") or (MH "Nutrients+") or (MH "Minerals+") or (MH "Vitamin A+") or (MH "Riboflavin") or (MH "Niacin") or (MH "Vitamin B Complex+") or (MH "Ascorbic Acid") or (MH "Vitamin D") or (MH "Vitamin E") or (MH "Vitamin K") or (MH "Prothrombin Time") or (MH "Calcium") or (MH "Magnesium") or (MH "Potassium") or (MH "Sodium, Dietary+") or (MH "Sodium") or (MH "Chromium") or (MH "Fluorides") or (MH "Iodine") or (MH "Iron") or (MH "Manganese") or (MH "Molybdenum") or (MH "Selenium") or (MH "Zinc")	Expanders - Apply equivalent subjects<br>Search modes - Boolean/Phrase	Interface - EBSCOhost Research Databases<br>Search Screen - Advanced Search<br>Database - CINAHL with Full Text
S14	S12 OR S13	Expanders - Apply equivalent subjects<br>Search modes - Boolean/Phrase	Interface - EBSCOhost Research Databases<br>Search Screen - Advanced Search<br>Database - CINAHL with Full Text
S13	TI ((laboratory N2 (marker or markers or values or test* or technique* or service or services or diagnos* or examin* or evaluat*)) or ("lab test" or "lab tests" or "lab result" or "lab results" or "lab value" or "lab values" or deficien*)) or AB ((laboratory N2 (marker or	Expanders - Apply equivalent subjects<br>Search modes - Boolean/Phrase	Interface - EBSCOhost Research Databases<br>Search Screen - Advanced Search<br>Database - CINAHL with Full Text

© Mayo Clinic Libraries, Mayo Foundation for Medical Education and Research

	markers or values or test* or technique* or service or services or diagnos* or examin* or evaluat*)) or ("lab test" or "lab tests" or "lab result" or "lab results" or "lab value" or "lab values" or deficien*))		
S12	(MH "Clinical Laboratories+")	Expanders - Apply equivalent subjects<br>Search modes - Boolean/Phrase	Interface - EBSCOhost Research Databases<br>Search Screen - Advanced Search<br>Database - CINAHL with Full Text
S11	S3 OR S9 OR S10	Expanders - Apply equivalent subjects<br>Search modes - Boolean/Phrase	Interface - EBSCOhost Research Databases<br>Search Screen - Advanced Search<br>Database - CINAHL with Full Text
S10	TI ((chemo* or antineoplas* or anti-neoplas* or anticancer* or anti-cancer* or anticarcino* or anti-carcino* or antitumor or anti-tumor or antitumour or anti-tumour or carcinochemo* or carcinostatic or "tumor inhibitor" or "tumour inhibitor") N3 (neuropath* or neuralgia or neurotox* or mononeuropath* or mono-neuropath* or polyneuropath* or poly-neuropath*)) or AB ((chemo* or antineoplas* or anti-neoplas* or anticancer* or anti-cancer* or anticarcino* or anti-carcino* or antitumor or anti-tumor or antitumour or anti-tumour or carcinochemo* or carcinostatic or "tumor inhibitor" or "tumour inhibitor") N3 (neuropath* or neuralgia or neurotox* or mononeuropath* or mono-neuropath* or polyneuropath* or poly-neuropath*))	Expanders - Apply equivalent subjects<br>Search modes - Boolean/Phrase	Interface - EBSCOhost Research Databases<br>Search Screen - Advanced Search<br>Database - CINAHL with Full Text
S9	S7 AND S8	Expanders - Apply equivalent subjects<br>Search modes - Boolean/Phrase	Interface - EBSCOhost Research Databases<br>Search Screen - Advanced Search<br>Database - CINAHL with Full Text
S8	(MH "Antineoplastic Agents+") or (MH "Chemotherapy, Adjuvant+") or TI ((anticancer* or anti-cancer* or anticarcino* or anti-carcino* or antitumor or anti-tumor or antitumour* or anti-tumour* or carcinostatic) N2 (therap* or agent or agents or treatment* or management*)) or carcinochemo* or "tumor inhibitor" or "tumour inhibitor") or AB ((anticancer* or anti-cancer* or anticarcino* or anti-carcino* or antitumor or anti-tumor or antitumour* or anti-tumour* or carcinostatic) N2 (therap* or agent or agents or treatment* or management*)) or carcinochemo* or "tumor inhibitor" or "tumour inhibitor")	Expanders - Apply equivalent subjects<br>Search modes - Boolean/Phrase	Interface - EBSCOhost Research Databases<br>Search Screen - Advanced Search<br>Database - CINAHL with Full Text
S7	S4 OR S5 OR S6	Expanders - Apply equivalent subjects	Interface - EBSCOhost Research Databases

© Mayo Clinic Libraries, Mayo Foundation for Medical Education and Research

		Search modes - Boolean/Phrase	Search Screen - Advanced Search<br>Database - CINAHL with Full Text
S6	TI (PNS N2 (disease* or disorder*)) or AB (PNS N2 (disease* or disorder*))	Expanders - Apply equivalent subjects<br>Search modes - Boolean/Phrase	Interface - EBSCOhost Research Databases<br>Search Screen - Advanced Search<br>Database - CINAHL with Full Text
S5	TI ((peripheral N2 (nerve* or nervous) N2 (disease* or disorder*)) or "peripheral neuropath*") or AB ((peripheral N2 (nerve* or nervous) N2 (disease* or disorder*)) or "peripheral neuropath*")	Expanders - Apply equivalent subjects<br>Search modes - Boolean/Phrase	Interface - EBSCOhost Research Databases<br>Search Screen - Advanced Search<br>Database - CINAHL with Full Text
S4	(MH "Peripheral Nervous System Diseases+")	Expanders - Apply equivalent subjects<br>Search modes - Boolean/Phrase	Interface - EBSCOhost Research Databases<br>Search Screen - Advanced Search<br>Database - CINAHL with Full Text
S3	S1 OR S2	Expanders - Apply equivalent subjects<br>Search modes - Boolean/Phrase	Interface - EBSCOhost Research Databases<br>Search Screen - Advanced Search<br>Database - CINAHL with Full Text
S2	(MH "Neuralgia+/CI")	Expanders - Apply equivalent subjects<br>Search modes - Boolean/Phrase	Interface - EBSCOhost Research Databases<br>Search Screen - Advanced Search<br>Database - CINAHL with Full Text
S1	(MH "Peripheral Nervous System Diseases+/CI")	Expanders - Apply equivalent subjects<br>Search modes - Boolean/Phrase	Interface - EBSCOhost Research Databases<br>Search Screen - Advanced Search<br>Database - CINAHL with Full Text

9. <u>Epistemonikos</u>

Cochrane Database of Systematic Reviews (CDSR) Last searched: Jun 3, 2020
Pubmed Last searched: Jun 3, 2020
EMBASE Last searched: May 29, 2020
CINAHL (The Cumulative Index to Nursing and Allied Health Literature) Last searched: May 29, 2020
PsycINFO Last searched: May 29, 2020
LILACS (Literatura Latinoamericana y del Caribe en Ciencias de la Salud) Last searched: May 29, 2020

© Mayo Clinic Libraries, Mayo Foundation for Medical Education and Research

Database of Abstracts of Reviews of Effects (DARE) Last searched: Aug 24, 2017 - No new records have been added to DARE after 2015
The Campbell Collaboration online library Last searched: May 30, 2020
JBI Database of Systematic Reviews and Implementation Reports Last searched: Jun 1, 2020
EPPI-Centre Evidence Library Last searched: Mar 9, 2020

Databases regularly updated:
Cochrane Database of Systematic Reviews (CDSR) Last searched: Dec 8, 2021
Pubmed Last searched: Dec 8, 2021
EMBASE Last searched: Dec 7, 2021
CINAHL (The Cumulative Index to Nursing and Allied Health Literature) Last searched: Dec 7, 2021
PsycINFO Last searched: Dec 7, 2021
LILACS (Literatura Latinoamericana y del Caribe en Ciencias de la Salud) Last searched: Dec 7, 2021
Database of Abstracts of Reviews of Effects (DARE) Last searched: Aug 24, 2017 - No new records have been added to DARE after 2015
The Campbell Collaboration online library Last searched: Nov 23, 2020
JBI Database of Systematic Reviews and Implementation Reports Last searched: May 1, 2021
EPPI-Centre Evidence Library Last searched: Mar 9, 2020
Other search sources
All of the systematic reviews being summarized in structured summaries, broad syntheses, and methodological studies (which is an exclusion criteria from Epistemonikos) are also included in the database.

Search #1
(title:(Peripheral Neuropathy) OR abstract:(Peripheral Neuropathy)) AND (title:(hemoglobin OR hemoglobins OR haemoglobin OR hemoglobine OR haemoglobine OR haemoglobins OR hemoglobulin OR hematocrit OR ferrohaemoglobin OR ferrohemoglobin OR erythrocytes OR eryhem OR albumin OR albumen OR prealbumin OR pre-albumin OR total-protein OR leptin OR globulin OR globulins OR polyglobulin OR poly-globulin OR choline OR bursine OR fagine OR vidine OR thiamin OR thiamine OR anemi* OR anaemi* OR aneurin OR riboflavin OR riboflavine OR "flavin mononucleotide" OR "flavin-adenine dinucleotide" OR niacin OR niacinamide OR enduramide OR nicobion OR nicotinamide OR nicotinsaureamid OR papulex OR "pantothenic acid" OR "calcium pantothenate" OR dexol OR "zinc pantothenate" OR "pyridoxal phosphate" OR "pyridoxal 5-phosphate" OR "pyridoxal-P" OR biotin OR folate OR "folic acid" OR folacin OR folvite OR folvit OR "pteroylglutamic acid" OR cobalamin OR cobalamins OR cyanocobalamin OR eritron OR "B1" OR "B2" OR "B3" OR "B5" OR "B6" OR "B7" OR "B9" OR "B12" OR "methylmalonic acid" OR "methyl malonate" OR "methyl malonic acid" OR methylmalonate OR retinol OR carotene OR "Aquasol A" OR phytomenadione OR menaquinone OR menadione OR calcium OR chloride OR magnesium OR phosphorus OR romag OR potassium OR kalium OR sodium OR chromium OR copper OR fluoride OR iodine OR iron OR "Fe" OR "Fe+++" OR ferro OR ferrum OR manganese OR molybdenum OR selenium OR zinc) OR abstract:(hemoglobin OR hemoglobins OR haemoglobin OR hemoglobine OR haemoglobine OR haemoglobins OR hemoglobulin OR hematocrit OR ferrohaemoglobin OR ferrohemoglobin OR erythrocytes OR eryhem OR albumin OR albumen OR prealbumin OR pre-albumin OR total-protein OR leptin OR globulin OR globulins OR polyglobulin OR poly-globulin OR choline OR bursine OR fagine OR vidine OR thiamin OR thiamine OR anemi* OR anaemi* OR aneurin OR riboflavin OR riboflavine OR "flavin mononucleotide" OR "flavin-adenine dinucleotide" OR niacin OR niacinamide OR enduramide OR nicobion OR nicotinamide OR nicotinsaureamid OR papulex OR "pantothenic acid" OR "calcium pantothenate" OR dexol OR "zinc pantothenate" OR "pyridoxal phosphate" OR "pyridoxal 5-phosphate" OR "pyridoxal-P" OR biotin OR

© Mayo Clinic Libraries, Mayo Foundation for Medical Education and Research

folate OR "folic acid" OR folacin OR folvite OR folvit OR "pteroylglutamic acid" OR cobalamin OR cobalamins OR cyanocobalamin OR eritron OR "B1" OR "B2" OR "B3" OR "B5" OR "B6" OR "B7" OR "B9" OR "B12" OR "methylmalonic acid" OR "methyl malonate" OR "methyl malonic acid" OR methylmalonate OR retinol OR carotene OR "Aquasol A" OR phytomenadione OR menaquinone OR menadione OR calcium OR chloride OR magnesium OR phosphorus OR romag OR potassium OR kalium OR sodium OR chromium OR copper OR fluoride OR iodine OR iron OR "Fe" OR "Fe+++" OR ferro OR ferrum OR manganese OR molybdenum OR selenium OR zinc)) AND (title:(Chemotherapy-Induced OR "Chemotherapy Induced" OR "chemically induced") OR abstract:(Chemotherapy-Induced OR "Chemotherapy Induced" OR "chemically induced"))
Results: 6
Results: 8

Search #2
(title:((title:(nutrition* OR nutrient* OR vitamin* OR mineral* OR anemi*) OR abstract:(nutrition* OR nutrient* OR vitamin* OR mineral* OR anemi*)) AND (title:(Chemotherapy-Induced Peripheral Neuropathy) OR abstract:(Chemotherapy-Induced Peripheral Neuropathy))) OR abstract:((title:(nutrition* OR nutrient* OR vitamin* OR mineral* OR anemi*) OR abstract:(nutrition* OR nutrient* OR vitamin* OR mineral* OR anemi*)) AND (title:(Chemotherapy-Induced Peripheral Neuropathy) OR abstract:(Chemotherapy-Induced Peripheral Neuropathy))))
Results: 16
Unique results: 11
Total unique results from Epistemonikos: 17

Results: 21
Unique results: 16
Total unique results from Epistemonikos in updated search: 24

Grey Literature – all resources searched June 05, 2020. All updated searches were completed on December 08, 2021.

10. ClinicalTrials.gov http://www.clinicaltrials.gov/

Searched June 05, 2020	**Search terms**	**# total results**	**# relevant/ non-duplicate results**
	Condition or disease: Chemotherapy-induced Peripheral Neuropathy<br>Other terms: laboratory or tests	65	1
	Condition or disease: Chemotherapy-induced Peripheral Neuropathy<br>Other terms: nutrition	2	0
	Condition or disease: Chemotherapy-induced Peripheral Neuropathy<br>Other terms: nutrient	8	8

© Mayo Clinic Libraries, Mayo Foundation for Medical Education and Research

	Condition or disease: Chemotherapy-induced Peripheral Neuropathy<br>Other terms: vitamin	9	1
	Condition or disease: Chemotherapy-induced Peripheral Neuropathy<br>Other terms: mineral	0	0
	Condition or disease: Chemotherapy-induced Peripheral Neuropathy<br>Other terms: micronutrient	8	0
Total			10

Searched December 08, 2021	**Search terms**	**# total results**	**# relevant/ non-duplicate results**
	Condition or disease: Chemotherapy-induced Peripheral Neuropathy<br>Other terms: laboratory or tests	5	5
	Condition or disease: Chemotherapy-induced Peripheral Neuropathy<br>Other terms: nutrition	3	1
	Condition or disease: Chemotherapy-induced Peripheral Neuropathy<br>Other terms: nutrient	1	0
	Condition or disease: Chemotherapy-induced Peripheral Neuropathy<br>Other terms: vitamin	9	0
	Condition or disease: Chemotherapy-induced Peripheral Neuropathy<br>Other terms: mineral	0	0
	Condition or disease: Chemotherapy-induced Peripheral Neuropathy<br>Other terms: micronutrient	8	0
Total			6

11. medRxiv https://www.medrxiv.org/

Searched June 05, 2020	**Search terms**	**# total results**	**# relevant/ non-duplicate results**
	Chemotherapy-induced Peripheral Neuropathy	3	0
	Peripheral Neuropathy and nutrition	17	0
	Peripheral Neuropathy and vitamin	14	0

© Mayo Clinic Libraries, Mayo Foundation for Medical Education and Research

	Peripheral Neuropathy and mineral	5	0
	Peripheral Neuropathy and nutrient	7	0
Total			0

Searched December 08, 2021	**Search terms**	**# total results**	**# relevant/ non-duplicate results**
	Chemotherapy-induced Peripheral Neuropathy	15	2
	Peripheral Neuropathy and nutrition	77	0
	Peripheral Neuropathy and vitamin	46	0
	Peripheral Neuropathy and mineral	19	0
	Peripheral Neuropathy and nutrient	25	0
Total			2

12. NICE Evidence https://www.evidence.nhs.uk/

Searched June 05, 2020	**Search terms**	**# total results**	**# relevant/ non-duplicate results**
	Chemotherapy-induced Peripheral Neuropathy nutrition	93	0
	Chemotherapy-induced Peripheral Neuropathy nutrient	15	0
	Chemotherapy-induced Peripheral Neuropathy vitamin	69	3
	Chemotherapy-induced Peripheral Neuropathy mineral	31	0
Total			3

Searched December 08, 2021	**Search terms**	**# total results**	**# relevant/ non-duplicate results**
	Chemotherapy-induced Peripheral Neuropathy nutrition<br>*Date filter*<br>From: 01/01/2020 [MM/DD/YYYY]<br>To: 12/08/2021 [MM/DD/YYYY]	6	0
	Chemotherapy-induced Peripheral Neuropathy nutrient<br>*Date filter*	1	0

© Mayo Clinic Libraries, Mayo Foundation for Medical Education and Research

	From: 01/01/2020 [MM/DD/YYYY]<br>To: 12/08/2021 [MM/DD/YYYY]		
	Chemotherapy-induced Peripheral Neuropathy vitamin<br>*Date filter*<br>From: 01/01/2020 [MM/DD/YYYY]<br>To: 12/08/2021 [MM/DD/YYYY]	5	1
	Chemotherapy-induced Peripheral Neuropathy mineral<br>*Date filter*<br>From: 01/01/2020 [MM/DD/YYYY]<br>To: 12/08/2021 [MM/DD/YYYY]	4	0
Total			1

13. OpenGrey http://www.opengrey.eu/

Searched June 05, 2020	**Search terms**	**# total results**	**# relevant/ non-duplicate results**
	Chemotherapy-induced Peripheral Neuropathy	1	0
	Peripheral Neuropathy nutrition	0	0
	Peripheral Neuropathy nutrient	0	0
	Peripheral Neuropathy vitamin	1	0
	Peripheral Neuropathy mineral	1	0
Total			0

OpenGrey is now archived at DANS EASY Archive https://doi.org/10.17026/dans-xtf-47w5**.**

Searched December 08, 2021	**Search terms**	**# total results**	**# relevant/ non-duplicate results**
	Chemotherapy-induced Peripheral Neuropathy	2	0
	Peripheral Neuropathy nutrition	0	0
	Peripheral Neuropathy nutrient	0	0
	Peripheral Neuropathy vitamin	0	0
	Peripheral Neuropathy mineral	0	0
Total			0

14. PROSPERO https://www.crd.york.ac.uk/prospero/

© Mayo Clinic Libraries, Mayo Foundation for Medical Education and Research

Searched June 05, 2020	**Search terms**	**# total results**	**# relevant/ non-duplicate results**
	Chemotherapy-induced Peripheral Neuropathy	26	0
	Peripheral Neuropathy laboratory	0	0
	Peripheral Neuropathy "lab tests"	0	0
	Peripheral Neuropathy "lab values"	0	0
Total			0

Searched December 08, 2021	**Search terms**	**# total results**	**# relevant/ non-duplicate results**
	Chemotherapy-induced Peripheral Neuropathy *Only considered records registered after January 01, 2020.*	55	1
	Peripheral Neuropathy laboratory *Only considered records registered after January 01, 2020.*	0	0
	Peripheral Neuropathy "lab tests" *Only considered records registered after January 01, 2020.*	0	0
	Peripheral Neuropathy "lab values" *Only considered records registered after January 01, 2020.*	0	0
Total			1

© Mayo Clinic Libraries, Mayo Foundation for Medical Education and Research
